# Probing regulon of ArcA in *Shewanella oneidensis *MR-1 by integrated genomic analyses

**DOI:** 10.1186/1471-2164-9-42

**Published:** 2008-01-25

**Authors:** Haichun Gao, Xiaohu Wang, Zamin K Yang, Timothy Palzkill, Jizhong Zhou

**Affiliations:** 1 Institute for Environmental Genomics and Department of Botany and Microbiology, University of Oklahoma, Norman, Oklahoma 73019, USA; 2 Environmental Sciences Division, Oak Ridge National Laboratory, Oak Ridge, Tennessee 37831, USA; 3 Center for Microbial Ecology, Michigan State University, East Lansing, Michigan 48824, USA; 4 Department of Molecular Virology and Microbiology, Baylor College of Medicine, Houston, Texas 77030, USA

## Abstract

**Background:**

The Arc two-component system is a global regulator controlling many genes involved in aerobic/anaerobic respiration and fermentative metabolism in *Escherichia coli*. *Shewanella oneidensis *MR-1 contains a gene encoding a putative ArcA homolog with ~81% amino acid sequence identity to the *E. coli *ArcA protein but not a full-length *arcB *gene.

**Results:**

To understand the role of ArcA in *S. oneidensis*, an *arcA *deletion strain was constructed and subjected to both physiological characterization and microarray analysis. Compared to the wild-type MR-1, the mutant exhibited impaired aerobic growth and a defect in utilizing DMSO in the absence of O_2_. Microarray analyses on cells grown aerobically and anaerobically on fumarate revealed that expression of 1009 genes was significantly affected (*p *< 0.05) by the mutation. In contrast to *E. coli *ArcA, the protein appears to be dispensable in regulation of the TCA cycle in *S. oneidensis*. To further determine genes regulated by the Arc system, an ArcA recognition weight matrix from DNA-binding data and bioinformatics analysis was generated and used to produce an ArcA sequence affinity map. By combining both techniques, we identified an ArcA regulon of at least 50 operons, of which only 6 were found to be directly controlled by ArcA in *E. coli*.

**Conclusion:**

These results indicate that the Arc system in *S. oneidensis *differs from that in *E. coli *substantially in terms of its physiological function and regulon while their binding motif are strikingly similar.

## Background

*Shewanella oneidensis *MR-1 is a facultative gram-negative anaerobe with remarkable anaerobic respiration abilities that allow the use of a diverse array of terminal electron acceptors. These acceptors include fumarate, nitrate, nitrite, thiosulfate, elemental sulfur, trimethylamine *N*-oxide (TMAO), dimethyl sulfoxide (DMSO), Fe(III), Mn(III) and (IV), Cr(VI), and U(VI) [1]. Because of this exceptional metabolic flexibility and the potential use of this organism for the bioremediation of metal/radionuclide contaminants in the environment, *S. oneidensis *MR-1 has been extensively studied and its genome has been sequenced [2]. However, little is known on how this bacterium adopts different metabolic modes in response to the availability of oxygen. In *Escherichia coli*, the global regulator Fnr (fumarate nitrate regulator) plays a major role in altering gene expression between aerobic and anaerobic conditions. In contrast, *S. oneidensis *MR-1 appears to employ Crp (cyclic-AMP receptor protein) rather than EtrA (electron transport regulator, *S. oneidensis *analog to *E. coli *Fnr) and possibly other unidentified proteins in regulating anaerobic respiration [3-5].

Arc (aerobic respiration control) is another system playing a role in oxygen-sensing and regulating anaerobic respiration in *E. coli *[6]. As a classical two-component system, Arc consists of the transmembrane sensor kinase ArcB and the DNA binding response regulator ArcA [7]. Under anaerobic or microaerobic respiratory conditions, ArcB undergoes autophosphorylation by sensing the redox state of quinone pool [8,9]. The phosphorylated ArcB then transfers a phosphate group to ArcA through a phospho-relay mechanism, resulting in phosphorylated ArcA (ArcA-P) [7,10,11]. ArcA-P functions as either an activator or repressor in mediating downstream genes by binding to DNA in the promoter regions of the target genes [11]. Gene expression profiling has revealed that more than a thousand genes in the *E. coli *genome are regulated either directly or indirectly by the ArcA protein [12,13].

*S. oneidensis *MR-1 ArcA shared more than 80% in amino acid sequence identity to its homologs in a number of bacterial species in *Escherichia*, *Salmonella*, *Yersinia*, *Erwinia*, *Photorhabdus*, *Vibrio*, and *Shigella *[6,8,14-16]. In addition, the Asp^54 ^residue in the N-terminal receiver domain and the helix-turn-helix (HTH) DNA-binding motif in the carboxy-terminal domain are structurally conserved. However, the genome lacks a definitive full-length *arcB *gene. ArcA of *S. oneidensis *MR-1 has been proven functional and involved in the oxygen response as well as in respiration of DMSO and in the detachment of cells from biofilms [14,17,18]. SO1327 (HptA) of *S. oneidensis*, exhibiting a significant degree of similarity to the Hpt domain of the *E. coli *ArcB, has been proposed to function to transfer phosphate groups to ArcA [14]. However, whether HptA is able to phosphorylate ArcA either *in vitro *or *in vivo *remains unanswered except that an *hptA *deletion strain was only slightly deficient in utilizing DMSO [14]. Furthermore, proteins equivalent to the sensor and/or the additional phosphotransfer domains remain unidentified.

The purpose of this genome-based study is to understand how the *S. oneidensis *MR-1 Arc system affects expression of genes under aerobic and anaerobic conditions. To this end, an *arc*A knockout mutational strain was constructed and subjected to physiological characterization and transcriptomic analysis. Results revealed that the mutation in *arcA *has a profound effect on the bacterial physiology and transcriptome. Meanwhile, an ArcA recognition weight matrix was generated using promoter regions of the core members of the Arc system to estimate the operons directly controlled by ArcA. Comparatively, this atypical Arc system differs from the *E. coli *Arc system substantially in terms of both functionality and regulon.

## Results

### Generation and verification of an *arcA *deletion strain

A mutagenesis system for constructing deletion mutants in *S. oneidensis *MR-1 has previously been developed and successfully utilized [19,20]. The *arcA *deletion mutant, designated as JZ3988K (*ΔarcA*), was constructed using the plasmid pDS3.1 following our established procedure as described in Methods. The deletion was confirmed by PCR, DNA sequencing and phenotype complementation. The complementation plasmid pBBR-ARCA was constructed and introduced into the *arcA *deletion strain as described in Methods. Two consistent phenotypes were identified: aerobic growth defect and anaerobic growth defect on DMSO as presented in the next section and reported previously, respectively [14]. The wild-type and the mutant strains containing empty plasmid pBBR1MCS-5 were included as controls. In all cases, physiological differences were insignificant between the *arcA *mutation strain containing plasmid pBBR-ARCA and the wild-type. These results verified that the phenotype of the *arcA *mutant is specific to the mutation in the *arcA *gene.

### Physiological characterization of the Δ*arcA *strain

**Figure 1:**
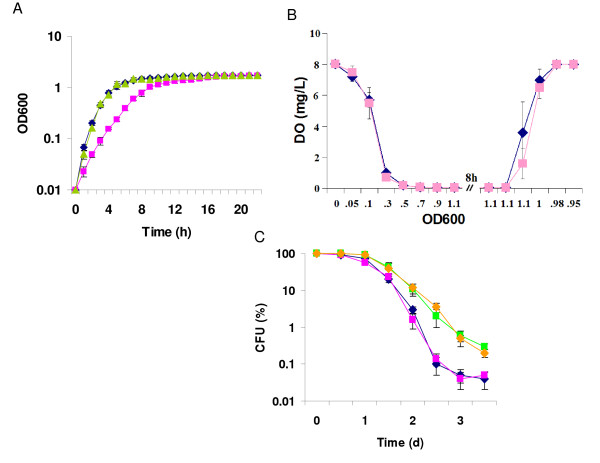
**Characteristics of *S. oneidensis *MR-1 and mutant strains under various conditions**. MR-1 (blue Diamond), JZ3988K (purple Square). (A) Growth under aerobic conditions, Complementation of JZ3988K with pBBR-ARCA (light green triangle) was also shown. (B) Dissolved oxygen concentration in MR-1 and JZ3988K cultures was plotted against OD_600 _values. (C) Survival rates during the stationary phase under aerobic conditions in shaken (blue diamond and purple Square) and still cultures (yellow diamond and green square) were shown.

Under aerobic conditions, growth of the *ΔarcA *strain was substantially slower than that of its parental strain MR-1 (Figure 1A). Although oxygen *per se *is not the direct signal for activation of the Arc system, the system functions to respond to redox conditions of growth [10]. Therefore, the observed growth difference may result from variation of culture oxygen levels in MR-1 and the mutant cultures. To rule out this possibility, levels of dissolved oxygen (DO) in both cultures were measured. As shown in Figure 1B, DO decreased quickly when cells grew up at the early stage. When cells entered the mid-log phase, DO reached the lowest point (0.06 mg/L) and remained at the level until the late stationary phase. Through the entire process, DO appeared to be irrespective of different strains but a function of cell density. All these results suggest that the Arc system of *S. oneidensis *has a role in the bacterial aerobiosis. Complementation of the *ΔarcA *strain with the pBBR-ARCA plasmid restored a growth rate similar to that of the wild-type aerobically. Under anaerobic conditions, differences in growth rate and maximum cell density between the mutant and wild-type strains were statistically insignificant when one of the following electron acceptors was used; fumarate (20 mM), nitrite (1 mM), thiosulfate (3 mM), TMAO (20 mM), MnO_2 _(5 mM), ferric citrate (10 mM), and cobalt(III)-EDTA (200 μM) (data not shown). In agreement with a previous report, the mutant was severely defective in utilizing DMSO (20 mM) [14].

To determine possible involvement of the Arc system in cell viability during the stationary phase in *S. oneidensis*, survival of the *ΔarcA *strain during this phase was examined in both still and shaking cultures as described in Materials and Methods. While cells of the wild-type and mutant strains died more quickly in the shaking cultures, little difference in survival rates between the wild-type and *ΔarcA *strains was observed under either condition. These results implicate that ArcA is dispensable in maintaining the viability of *S. oneidensis *cells during stasis (Figure 1C).

### Global transcriptomic analysis of the Δ*arcA *strain

Microarray analysis was employed to dissect the transcriptomic differences elicited by the mutation in *arcA *during aerobiosis and anaerobiosis. For aerobiosis, the wild-type and mutant cells at exponential phase were used because the phenotypic difference was most significant during this period. Although little difference in physiology was observed between the wild-type and *ΔarcA *strains during anaerobiosis with fumarate as the sole electron acceptor, the exponential stage cells were collected for this study to serve two purposes. First, this may facilitate our understanding of ArcA's role during anaerobiosis. Second, this enables us to compare transcriptomes of *E. coli arcA *mutant to *S. oneidensis arcA *mutant because microarray analyses on *E. coli arcA *muant have been conducted under the similar conditions [12,13]. The quality of microarray data was assessed with two approaches used as a standard in our laboratory. First, the high quality of the expression data was validated with a statistical analysis as previously described [21]. Second, 8 ORFs were selected for real-time quantitative reverse transcription-PCR (qRT-PCR) analysis with the same RNA samples used in the array hybridizations based on the level and reproducibility of changes observed in the microarray experiments. A high level of concordance (R^2 ^= 0.96) was observed between microarray and real-time qRT-PCR data despite quantitative differences in the level of change, suggesting that the microarray results are an accurate reflection of the gene expression profile (Figure 1S in additional file 1).

**Figure 2:**
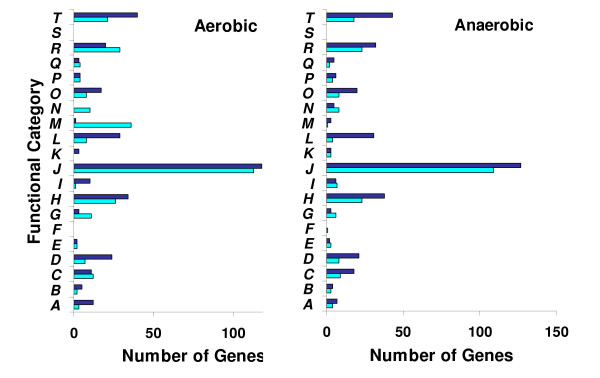
**Differentially expressed genes grouped by functional classification according to the TIGR *S. oneidensis *genome database**. A, Amino acid biosynthesis; B, Biosynthesis of cofactors, prosthetic groups, and carriers; C, Cell envelope; D. Cellular processes; E, Central intermediary metabolism; F, Disrupted reading frame; G, DNA metabolism; H, Energy metabolism; I, Fatty acid and phospholipid metabolism; J, Hypothetical proteins; K, Mobile and extrachromosomal element functions; L, Protein fate; M, Protein synthesis; N, Purines, pyrimidines, nucleosides, and nucleotides; O, Regulatory functions; P, Signal transduction; Q, Transcription; R, Transport and binding proteins; S, Unclassified; T, Unknown function. Bars in black are the genes that showed decreased expression in the presence of ArcA; bars in gray are the genes that showed increased expression in the presence of ArcA.

In total, 1009 genes passed ANOVA statistical analysis (p < 0.05) with Benjamini and Hochberg False Discovery Rate multiple testing correction in at least one of two hybridizations between JZ3988K and MR-1, representing approximately 21.7% of the 4,648 ORFs spotted on the array (Table S1 in additional file 2). Interestingly, only 12 genes responded oppositely under aerobic and anaerobic conditions while the majority of 1009 genes responded to the *arcA *mutation are irrespective to the availability of oxygen. The functional class distribution of these 1009 genes is shown in Fig. 2. Genes displaying significant differences in expression due to an *arcA *mutation under either aerobic or anaerobic conditions were observed in almost every category. The wide distribution of putative functional roles attributed to the differentially expressed genes indicates that ArcA has a global effect on gene expression in *S. oneidensis*. While up to 54% of the genes showed increased expression in the absence of ArcA under aerobic conditions, the percentage of this type of genes under anaerobic conditions increased to 60%. The most noticeable differences in gene numbers between tested conditions were observed in the categories of protein synthesis (M) and nucleotide synthesis (N). Under aerobic conditions, nearly all of genes in these two categories showed reduced expression in the absence of ArcA but very few genes in these categories were affected under anaerobic conditions.

**Figure 3:**
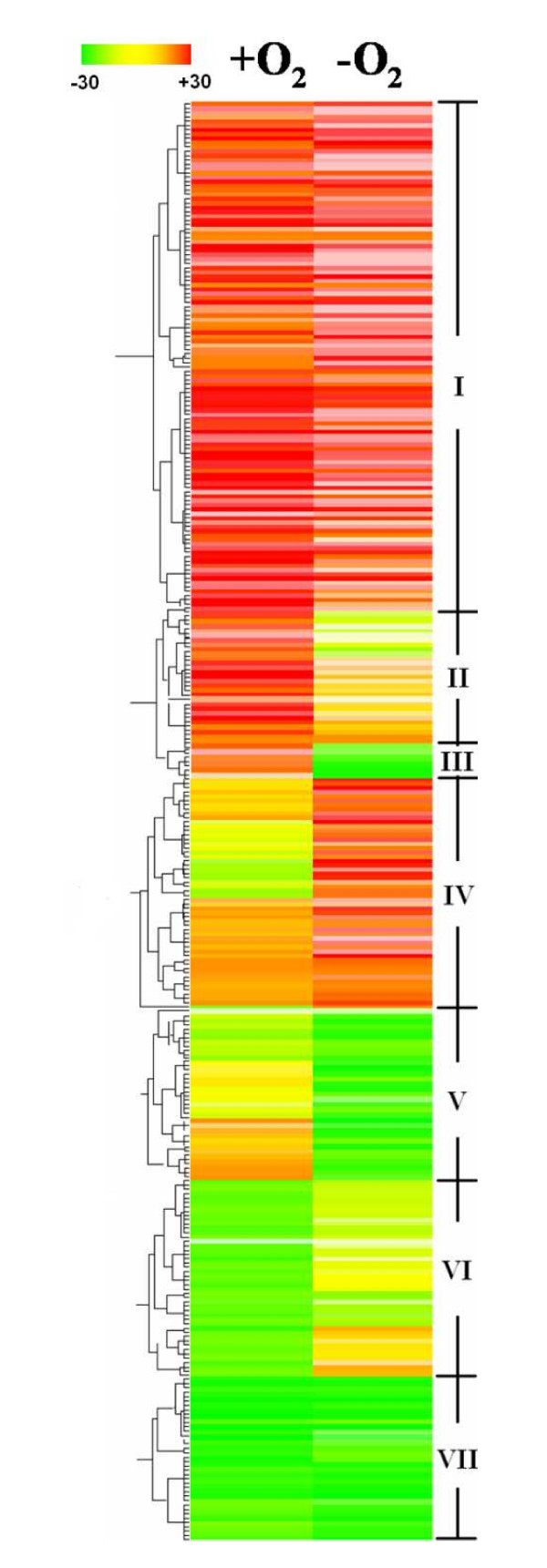
**Hierarchical clustering of selected genes**. All these genes are listed in Table S2 (in additional file 3). Expression differences (*ΔarcA*/MR-1) were represented by colors: red, induced, yellow, insignificant, and green, repressed. Each pattern is identified by different colors on the dendrogram and by numbers that correspond to the gene expression patterns. +O_2_, aerobic conditions; -O_2_, anaerobic conditions.

While 1009 genes significantly affected in terms of their level of expression by the *arcA *mutation provide a large amount of information, it is less practical to discuss all of them in detail. Thus we generated a high-confidence list of 317 genes with at least a 2-fold change in expression and an ANOVA *P *value of < 0.01 (Table S2 in additional file 3). To identify co-regulated patterns of gene expression, we classified these 317 differentially expressed genes into 7 hierarchical clusters based on their log ratio of expression (Fig. 3).

#### Expression Pattern I: Induced in the ΔarcA strain during either aerobiosis or anaerobiosis

Transcription of the 118 genes in this cluster was up-regulated in the *ΔarcA *strain under either aerobic or anaerobic conditions (Table S2 in additional file 3). 61 of these genes encode hypothetical proteins or proteins whose functions are presently unknown.

Genes encoding proteins in cellular processes include *so0866 *(putative minor curlin subunit CsgB), *acc *(aculeacin A acylase), *pilU *(twitching motility protein PilU), *so3685 *(putative curli production assembly/transport component CsgG), *so3686 *(putative curli production assembly/transport component CsgF), *so3687 *(putative curli production assembly/transport component CsgE), *so4149 *(putative RTX toxin), and *aggA *(agglutination protein). All but *pilU *or *so4149 *were reportedly involved in biofilm formation. This is not surprising because the involvement of the ArcA regulon in the development of biofilms has been firmly established in both *E. coli *and *S. oneidensis *[17,22].

Several genes for energy metabolism were also in this cluster. Operon *hoxK-hyaB-hydC *encodes three subunits of the quinone-reactive Ni/Fe hydrogenase which catalyzes the reversible oxidation of molecular hydrogen and plays a central role in microbial energy metabolism [23]. The operon has been proven to be directly under the control of ArcA [14]. Correspondingly, four genes of the operon *hypFBCDEA*, which encode proteins required for Ni/Fe hydrogenase (encoded by operon *hoxK-hyaB-hydC*) maturation, were found in this cluster [24].

ArcA appears to repress expression of a number of genes encoding regulatory proteins. These included *so0864 *(transcriptional regulator, LuxR family), *so0916 *(transcriptional regulator, MarR family), *rseA *(sigma-E factor negative regulatory protein), *so1661 *(transcriptional regulator, LysR family), *so1699 *(transcriptional regulator), *pspF *(*psp *operon transcriptional activator), *so3516 *(transcriptional regulator, LacI family), *so4542 *(transcriptional regulator, LacI family), and *rpoS *(alternative sigma factor σ^S^). Among these genes, only three have been well defined in *E. coli*. RseA is an anti-sigma factor that inhibits sigma E which transcribes genes that encode protein folding factors in response to extracytoplasmic stress stimuli [25]. The PspF protein belongs to the enhancer-binding protein family of sigma54-dependent activators and participates in controlling several genes involved in phage-shock, such as *pspABC *operon [26,27]. In *E. coli*, the stationary phase alternative sigma factor σ^s^, controls the expression of the genes involved in cell survival in response to cessation of growth (stationary phase) and provides cross-protection to various stresses [28]. Involvement of ArcA in *E. coli *stationary phase via catabolic control has also been established [29].

#### Expression Pattern II: Induced during aerobiosis but unaffected during anaerobiosis in the ΔarcA strain

Among the 317 genes, 29 showed increased expression under aerobic conditions but unchanged expression under anaerobic conditions in the mutant (Table S2 in additional file 3). It is reasonable to assume that these genes have a role during aerobiosis only.

**Figure 4:**
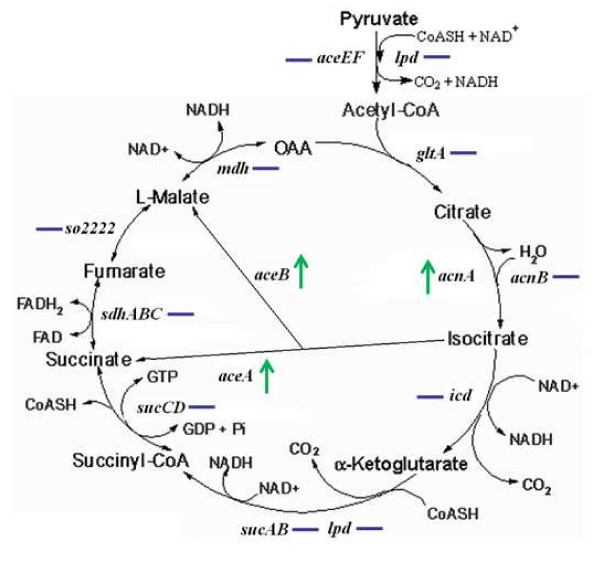
**Expression changes of genes in TCA cycle and glyoxylate pathway under aerobic conditions**. Changes were recorded as the ratio of expression in *ΔarcA *to that in MR-1, "--" represents unaffected by the mutation, "↑" represents up-regulated in the *arcA*^- ^strain.

Of the genes encoding proteins in this cluster, *acnA *(aconitate dydratase), *aceB *(malate synthetase A), and *aceA *(isocitrate lyase) were particularly worth noting. The *acnA *gene was the only one in TCA repressed by ArcA (Fig. 4). In contrast, ArcA represses transcription of the genes involved in entire TCA cycle except for *acnB *in *E. coli *[30]. Like AceB and AceA, AcnA is also a component in the glyoxylate pathway. The pathway short-circuits the TCA cycle and therefore rendering most of the TCA components unnecessary.

#### Expression Pattern III: Induced during aerobiosis but repressed during anaerobiosis in the ΔarcA strain

Among the 317 genes, 6 showed increased expression under aerobic conditions but reduced expression under anaerobic conditions in the mutant compared to the wild-type (Table S2 in additional file 3). Most of these genes encode proteins with unknown function.

#### Expression Pattern IV: Unaffected during aerobiosis but induced during anaerobiosis in the ΔarcA strain

A total of 54 genes were clustered into this pattern (Table S2 in additional file 3). Among these 54 genes, 22 encode proteins in energy metabolism. The most notable observation was that all members except *nuoK *of the *nuoA-N *operon, encoding NADH dehydrogenase I, were mildly induced in the mutant only under anaerobic conditions. Studies in *E. coli *revealed that expression of the *nuoA-N *operon is repressed by ArcA-P under anaerobic conditions [31]. NADH dehydrogenase I primarily functions to couple the transfer of electrons from NADH to ubiquinone with the translocation of protons across the membrane in *E. coli *and *Klebsiella pneumoniae *[32].

The remaining 14 genes included *astD *(succinylglutamic semialdehyde dehydrogenase), *deoC *(deoxyribose-phosphate aldolase), *torD *(TorA specific chaperone), *torA *(TMAO reductase), *torC *(tetraheme cytochrome c), *so1659 *(tetraheme cytochrome c), *ccoP *(cytochrome c oxidase, cbb3-type, subunit III), *ccoQ *(cytochrome c oxidase, cbb3-type, CcoQ subunit), *ccoO *(cytochrome c oxidase, cbb3-type, subunit II), *ccoN *(cytochrome c oxidase, cbb3-type, subunit I), *pflB *(formate acetyltransferase), *pflA *(pyruvate formate-lyase 1 activating enzyme), *ackA *(acetate kinase), and *pta *(phosphate acetyltransferase). Except for *so1659 *and *astD *whose product is involved in arginine degradation, all other genes were co-regulated with the majority of their operon members [33]. The *torCAD *genes encode an inducible TMAO respiratory system as observed in *E. coli *[34,35]. In addition to *deoC*, operon *deoC-B-A *contains other two genes *deoA *(thymidine phosphorylase) and *deoB *(phosphopentomutase) which have been classified into the functional category of purines, pyrimidines, nucleosides, and nucleotides by TIGR. The products of this operon are involved in (deoxy)ribose phosphate degradation in *E. coli *[36]. Cytochrome c oxidase (CcoN-O-Q-P), whose counterpart is not found in *E. coli *genome, has been well studied as a vital complex in oxidative phosphorylation [37]. Further exploration is needed to clarify why the operon is affected under anaerobic conditions. PflB is an enzyme catalysing the reversible reaction of pyruvate and coenzyme A into acetyl-CoA and formate after being activated by PflA under anaerobic conditions [38] while the versatile AckA-Pta have been reported to function in threonine degradation, acetate utilization, pyruvate oxidation, and mixed acid fermentation pathways under both aerobic and anaerobic conditions. AckA has been well studied as a member of ArcA regulon in *E. coli *[39].

Genes that belong to the category of transport and binding proteins also are enriched in this cluster. These included *so1033 *(putative iron-compound ABC transporter, ATP-binding protein), *so1034 *(iron-compound ABC transporter, permease protein), *so1882 *(AcrB/AcrD/AcrF family protein), *modA *(molybdenum ABC transporter, periplasmic molybdenum-binding protein), *modC *(molybdenum ABC transporter, ATP-binding protein), *so4281 *(putative potassium uptake protein KtrA), and *ktrB *(potassium uptake protein KtrB). Iron is an essential minor element for most organisms, playing vital roles in many important biological processes [40]. Although iron metabolism in *E. coli *is well studied, the iron-compound ABC transporter proteins in *E. coli *are still poorly defined. The possible *E. coli *genes corresponding to *so1033 *and *so1034 *are *fhuA *and *fecC*, respectively, which belong to two operons. It is not surprising that *modA *and *modC *fall into this category because the transition metal molybdenum is required for the enzymatic activities of most bacterial molybdoenzymes during anaerobiosis, including sulfite oxidase, nitrate reductase, DMSO reductase, and formate dehydrogenase [41]. KtrA and KtrB, two members of a new type of bacterial K^+^-uptake system, are peripheral and integral membrane proteins cooperating in K^+ ^translocation [42]. The system appears to be widespread and functions in the adaptation of cells to hyperosmotic conditions [43,44].

#### Expression Pattern V: Unaffected during aerobiosis but repressed during anaerobiosis in the ΔarcA strain

A total of 33 genes shared this expression pattern (Table S2 in additional file 3). Five genes (*cymA*, *omcA*, *omcB, napB, so3980*) encoding cytochrome *c *proteins, along with *dmaA-1 *and *dmsB-1 *encoding DMSO reductase, belong to this cluster. CymA, one of the most versatile cytochrome *c *proteins, supplies electrons to at least five different terminal reductases for utilizing fumarate, DMSO, nitrate, nitrite, and Fe(III) [45,46]. OmcA and OmcB have been reported to be involved in anaerobiosis, especially in Mn(IV) reduction [47]. Genes *dmaA-1 *and *dmsB-1 *are from operon *so1427-30 *which is directly controlled by ArcA-P and the rest two genes encode a cytochrome *c *protein (SO1427) included in the cluster V and an outer membrane protein (SO1428) in this cluster [14]. While DmaA-1 and DmsB-1 are functional subunits of DMSO reductase, SO1427 and SO1428 remain uncharacterized. Two genes (*so1431-2*) in this cluster encoding hypothetical proteins, locating immediately after the *so1427-30 *operon, have been listed as members of the *so1427-30 *operon [14]. However, an individual operon for these two genes has been predicted by two independent studies [48,49].

One of the most unexpected findings in this study was that three members (*napD*, *napA*, and *napB*) of the *nap *operon for nitrate reduction and *so3980 *(*nrfA*) for nitrite reduction were strongly repressed in the *ΔarcA *strain. In *S. oneidensis*, it has been demonstrated that the *nap *operon is essential for reduction of nitrate to nitrite in *S. oneidensis *[50]. Meanwhile, *so3980 *(*nrfA*) is essential for reduction of nitrite to ammonium (unpublished results). To verify this observation, expression of *napA *was measured by real-time qRT-PCR. The qRT-PCR results correlated well with those obtained from the microarrays as shown in Fig. 1S.

The *fadA *and *fadB *genes, consisting of an operon and encoding subunits of the fatty acid oxidation complex, belong to this cluster too. In *E. coli*, two fatty acid oxidation pathways (aerobic and anaerobic) have been characterized [51]. The FadAB complex functions in the aerobic fatty acid oxidation pathway only. However, it is arguable because the *fadBA *operon has been shown previously to be anaerobically repressed by the ArcA protein [12]. Our findings further indicated that ArcA has an effect on the *fadBA *operon under anaerobic conditions.

#### Expression Pattern VI: repressed during aerobiosis but unaffected during anaerobiosis in the ΔarcA strain

A total of 46 genes were clustered into this pattern. Most of the genes encoding ribosomal proteins and ATP synthase were found in this cluster (Table S2 in additional file 3). 20 out of 52 ribosomal structural genes (within *rpl*, *rpm*, and *rps *operons), along with genes (*fusA-1 *(translation elongation factor G), *secY *(SecY subunit of preprotein translocase), *rimM *(16S rRNA processing protein), *tsf *(translation elongation factor Ts), *frr *(ribosome recycling factor), *pyrH *(uridylate kinase), and *radC *(DNA repair protein RadC)) encoding ribosome related proteins within these three operons. 5 (*atpA*, *atpB*, *atpE*, *atpF*, and *atpH*) out of 9 members (*atpA-I*) of ATP synthase F1 and F0 were down-regulated during aerobiosis in the mutant strain. Proteins from both these subgroups belong to this macromolecule synthesis class. The ATP synthase of *E. coli *functions to synthesize ATP either by electron transport-link phosphorylation under aerobic conditions or by generation of an electrochemical proton gradient under anaerobic conditions and its regulation is largely under the control of the cell growth rate [52]. It is likely that the repression of these operons may result from the slower growth of mutant strain.

The remaining 13 genes included *petAB *(iron-sulfur & cytochrome b subunits of ubiquinol-cytochrome c reductase), *lrp *(leucine-responsive regulatory protein), *hugA *(heme transport protein), *purA *(adenylosuccinate synthetase) *so1770-1 *(glycerate kinase & GntP family permease), *so3300-1 *(cytochrome c proteins), and four encoding hypothetical proteins or protein with unknown functions. Cytochrome *bc*_1 _complex encoded by *petA *and *petB *contributes to the formation of membrane potential and proton gradient, which are coupled to ATP synthesis [37]. It is not surprising that expression of these two genes was consistent with expression of operon *atpA-H*. Lrp is a major regulatory protein involved in the expression of more than 30 operons largely in response to leucine in *E. coli *[53]. None of these operons have been reported to be regulated by ArcA. HugA, a predicted ATP transporter of protoheme, has been reported to be functionally related to the TonB energy transducing system [54]. Another member of the *hugA *operon, *so3667*, encoding a hypothetical protein, was also in this cluster.

#### Expression Pattern VII: Repressed during aerobiosis or anaerobiosis in the ΔarcA strain

This cluster contains 31 genes, 16 of which encode hypothetical proteins (Table S2 in additional file 3).

Genes in this cluster encoding transport and binding proteins include *so0919 *(putative serine transporter), *so1821 *(putative outer membrane porin), *emrD *(multidrug resistance protein D), *so2427 *(putative TonB-dependent receptor), *so2865 *(putative L-lysine exporter), *so3099 *(putative long-chain fatty acid transport protein), *so3706 *(NupC family protein), and *so4014 *(AcrB/AcrD/AcrF family protein). Genes encoding proteins in metabolic pathways include *speF *(inducible ornithine decarboxylase), *so1427 *(decaheme cytochrome c), and *so3705 *(putative 5-methylthioadenosine nucleosidase/S-adenosylhomocysteine nucleosidase). *SpeF *is the ornithine decarboxylase which helps cells against low environmental pH [55]. As the first gene of the operon *so1427-30 *which contains genes encoding DMSO reductase, *so1427 *differed in expression pattern from other members of the operon as discussed in pattern V. Also, genes *so1418 *(ApbE family protein), *so3969 *(OmpA family protein) and *so4681 *(glycosyl transferase, group 1 family protein) encode proteins in the functional category of cell envelope.

### Screening for target operons of ArcA(-P) by EMSA

The analyses presented thus far clearly showed that *S. oneidensis *ArcA differs substantially from its *E. coli *counterpart in its physiological role and regulates a large number of genes. Although the majority of these genes may be affected indirectly, those possessing an ArcA-binding site in their upstream region are likely to be controlled directly by ArcA. In *E. coli*, the consensus ArcA-P binding sequence has been concluded computationally on the basis of DNA footprinting data [12]. Given the high degree of conservation in sequence and structure between ArcA proteins of *E. coli *and of *S. oneidensis*, it is reasonable to assume that ArcA of *S. oneidensis *binds to a sequence similar to the *E. coli *consensus 15-bp stretch. To verify this assumption and to facilitate the determination of the consensus ArcA(-P) binding sequence in *S. oneidensis*, a electrophoretic motility shift assay (EMSA) was used to examine the ArcA(-P) binding activity of upstream sequences of selected operons with purified ArcA proteins.

**Figure 5:**
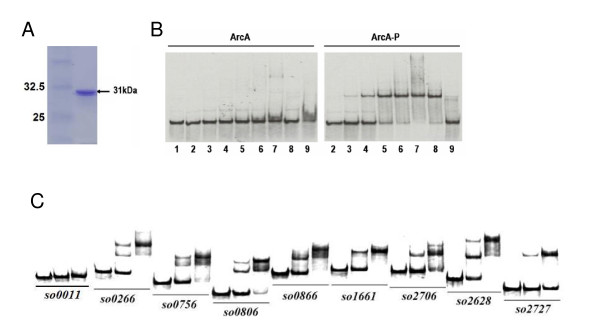
**ArcA(-P) Binding to selected promoters by EMSA**. (A). Overproduced and purified recombinant *S. oneidensis *His_6_-ArcA from *E. coli *BL21 cells. (B). Interaction of *so1661 *promoter DNA with *S. oneidensis *His_6_-ArcA. The probe was prepared by PCR with SO1661-EMSA-F (^33^P end-labeled) and SO1661-EMSA-R primers (Table S4 in additional file 5). The EMS assay was performed with 2 nM ^33^P end-labeled probes and various amounts of ArcA (left panel) or ArcA-P proteins (right panel). The protein concentrations for lanes 1–9 are 0, 0.125, 0.25, 0.5, 1.0, 2.0, 4.0, 4.0, 4.0 μM, respectively. Non-specific competitor DNA, (2 μg poly dI·dC), was added (lane 8) and specific competitor (10 μM unlabeled SO1661 probe) was added (lane 9). (C). The binding assay was performed in the presence of 0, 1, or 2 μM ArcA-P and 2–5 nM radiolabeled promoter DNA 0.2 μg/μl poly(dI·dC) was used in all these binding reactions to block non-specific interactions. Promoter region of *so0011 *(*gyrB*) was included as negative control. The phosphorylation of the ArcA protein was done with carbamoyl phosphate.

Expression of the *S. oneidensis *ArcA protein was initiated by insertion of the *arc*A gene into the Gateway entry vector pDONR221 using a lambda recombinase cloning strategy [56]. The entry vector containing the *arcA *gene was then converted to a protein expression system by recombination with the Gateway destination vector pDEST17 which resulted in the attachment of an N-terminal His-tag for protein purification. The His-tagged ArcA protein was expressed in *E. coli *and purified from inclusion bodies (Fig. 5A).

In total, PCR products containing upstream intergenic regions of 30 individual operons were generated and examined for their ArcA(-P) binding activities (Table 1). Among them, *gltA-sdhCAB*, *sucAB*, *icd*, *fdrCAB, acnB*, and *frdC *encoding enzymes involved in the TCA cycle were chosen to validate the observation from the microarray analysis that *S. oneidensis *ArcA appears to be dispensable in the process [7,57-59]. The rest of the operons selected met both or either of two criteria: a significant expression difference elicited by the *arcA *mutation and a 15-bp sequence in the upstream region similar to the *E. coli *consensus ArcA-P binding motif. In a preliminary experiment, the ArcA(-P) binding activity of the *so1661 *promoter region was tested with the purified unphosphorylated His-tagged *S. oneidensis *ArcA protein as well as the protein phosphorylated with carbamoyl phosphate in EMSA. Significant binding to the DNA probe occurred at a protein concentration of less than 0.25 μM for ArcA-P (Figure 5B), which is comparable to that from *E. coli *ArcA-P [7]. In contrast, the non-phoshorylated ArcA did not bind even when the protein concentration was increased to 4 μM (Figure 5B). The binding of ArcA-P to the target promoter was not reduced by addition of the nonspecific competitor poly(dI·dC) DNA, but was outcompeted by adding excess unlabeled probe (Figure 5B). These results demonstrate that phosphorylated ArcA binds the *so1661 *promoter in a sequence specific manner.

**Table 1:** Operons tested for their ArcA(-P) binding activity by EMSA

Operon	Fold change^a^				
	+O_2_	-O_2_	Position^b^	Putative motif^c^	Feature^d^
*so0266-9 (ccmF-1)*	1.07	0.94	-71	GTGAACAGAATGTTA	B & E
*so0314 (speF)*	-3.03	-2.79			E
*so0396 (frdC)*	-0.07	-0.27			TCA
*so0397-9 (fdrC)*	0.39	0.25			TCA
*so0432 (acnB)*	-0.54	-0.21			TCA
*so0756 (aroG)*	-0.84	-2.18	-164	TTTAAAAATATGTTA	B & E
*so0806*	3.22	3.64	-45	GAAAATTTTTTGTTA	B & E
*so0866*	7.00	8.08	-136	GTAATTTAAATGTTA	B & E
*so1307 (aqpZ)*	1.42	0.87	-197	GTTAACAAAGCGATA	B & E
*so1427-30*	-1.78	-4.38	-246	GTTAATAAAATGTTT	B & E
*so1623 (ptsG)*	2.00	3.26	-472	GTTACTTTATTGTTA	B & E
*so1661*	2.47	3.11	-219	GTTAAATAATTGTTA	B & E
*so1806 (pspF)*	2.63	2.42	-90	GTTAATAAAATGTTT	B & E
*so1821*	-2.25	-3.18	-230	GTTAATTTGATGTTA	B & E
*so1926 (gltA)*	-0.58	-0.32			TCA
*so1930-3 (sucA)*	0.18	0.66			TCA
*so1944*	1.12	1.80	-65	GTTAAGTAATTGTAA	B & E
*so2099-5 (hoxK)*	4.08	2.40	-147	GTTAATTAAATGTCA	B & E
*so2389 (emrD)*	-4.44	-3.18			E
*so2460*	5.73	6.83			E
*so2629 (icd)*	-0.07	-0.49			TCA
*so2706 (astB)*	0.96	1.95	-209	GTTGAAAAAATGTAA	B & E
*so2727*	-0.97	-1.47	-220	GTTATTCAAATGTAA	B & E
*so3099*	-5.64	-4.64	-281	GTTAATTAAATTATA	B & E
*so3106 (aprE)*	5.44	5.94	-127	GTAAATTAATTGTTA	B & E
*so3507*	-0.56	0.70	-90	GTTAACTCAATGTTA	B
*so3565 (cpdB)*	-1.89	2.10	-257	GTTAATTAATTGTTG	B & E
*so3659*	2.83	3.53	-205	*GTAAAATCGATGTTA*^e^	B & E
*so3855 (sfcA)*	0.36	0.49	-177	GTTAATTGATTGTAA	B
*so4245 (argA)*	0.19	0.41	-178	GTTAAAAAAATGTGA	B

^a^Log_2 _value. For multiple-gene Operons, only expression of the first gene was listed

^b^Referring to the translation start site

^c^Putative binding motifs were given with *E. coli *consensus sequence as the reference

^d^Operon features. B: binding motif; E: Expression difference statistically significant; TCA: Operon functions in TCA

^e^ArcA(-P) binding activity was not observed in EMSA and sequence was not used for constructing the weight matrix (Figure 6)

Among the tested PCR sequences in EMSA with phosphorylated ArcA, 20 showed ArcA(-P) binding activity (Figure 5C, only 8 were shown). Interestingly, all of these 20 sequences contained a 15 bp stretch sharing a high level similarity with *E. coli *ArcA-P binding consensus motif. Only one (*so3659*) such a sequence was not found to be capable of binding. In contrast, the retardation of the sequences without putative binding motif was not observed, including those (*so0314*, *so2389*, *so2460*) exhibiting an extremely strong expression difference between the mutant and the wild-type (data not shown). The promoter sequences of operons encoding enzymes in the TCA cycle, as expected, did not appear to interact with ArcA(-P) at all (data not shown). This result, along with our microarray data, ruled out the possible involvement of the Arc in TCA of *S. oneidensis*. Overall, the EMSA assays suggest that the binding motif is the most important factor determining the binding activity of promoters.

### Determination of genes directly under the control of ArcA(-P) in *S. oneidensis*

**Figure 6:**
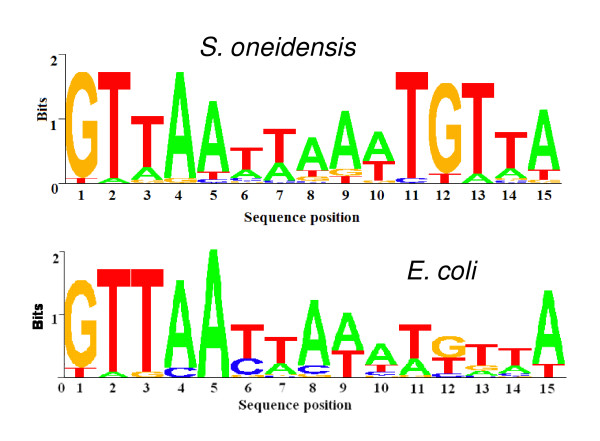
**Sequence logo for the ArcA-P recognition matrix in *S. oneidensis***. The sequences used were listed in Table 1. The sequence conservation, measured in bits, is shown as the height of a stack of letters at each base position. Sequence logo for the ArcA-P recognition matrix in *E. coli *was also shown as comparison. The *E. coli *sequences used were from the previous report by Liu and De Wulf [12].

Two recent studies suggest that more than a hundred operons are directly controlled by ArcA in *E. coli *[12,13]. While our physiological and microarray analyses demonstrated that ArcA of *S. oneidensis *may differ substantially from the canonical Arc system in terms of both functions and regulatees, the consensus ArcA-P binding sequences from these two microorganism are alike. In other words, the binding site is more deterministic than expression changes observed in microarray analysis. To screen for operons whose upstream region contains a binding site, an ArcA-binding weight matrix was constructed with 20 PCR sequences bound by ArcA-P revealed in the EMSA (Table 1). Highly conserved stretches of 15 base pairs were found in the upstream regions of all input genes using AlignACE and a weight matrix was generated from these sequences. A sequence logo was deduced to show the frequencies scaled relative to the information content of at each position. (Figure 6) [60,61]. Compared to the one in *E. coli*, subtle changes at most bases were noticeable although a high level of similarity remained. Especially, nucleotides at both ends of the binding motif are much less conserved in *S. oneidensis *while the 12^th ^nucleotide (G) appears to be important for binding.

The *S. oneidensis *genome was then scanned on either strand and scores of all successive 15-bp stretches were given using the log transformation method of Berg and von Hippel [62]. The average of total scores was assigned a Z score of 0 and sites with a Z score of 2.3 or greater and within 700 bp of an ORF origin were regarded as the potential ArcA-P binding sequences in *S. oneidensis*. The cutoff value was chosen based on an assessment that 372 genes are potential members of the ArcA regulon and the approximately 100–150 operons that may be under the transcriptional control of ArcA in *E. coli *[12]. In total, 209 operons containing 313 protein-encoding genes were predicted to contain potential ArcA-binding sites in their upstream regions (Table S3 in additional file 4).

By combining both microarray and weight matrix profilings (Table S1 and S3 in additional file 2 and 4, respectively), we identified at least 50 operons which could be under the direct control of ArcA in *S. oneidensis *(Table 2). In addition, operons next to a putative ArcA-binding site with a Z score 3.0 or above are automatically included as the candidates to be directly controlled by ArcA based on the criteria adopted in similar studies [12]. The majority of these operons encode proteins whose functions have not been determined yet, implicating a significant function shift. These newly identified operons (*i.e. so1427-30*, *so1661*, *so1821*) may represent lineage-specific ArcA regulon members.

**Table 2:** Operons that are most likely under the direct transcriptional control of ArcA

Operon^a^	Ratio (log2)^b^							
	+O_2_	-O_2_	FC^c^	Site^d^	S^e^	*Z*^f^	Binding sequence	Function of Operon
***so0021-0 (fadBA)***	0.67	-1.81	I	400	+	2.45	GTTAATAATAAATAT	Fatty oxidation complex
*so0266-9 (ccmF-1)*	1.07	0.94	H	71	+	2.63	GTGAACAGAATGTTA	Cytochrome c-type biogenesis protein CcmF
***so0343 (acnA)***	2.35	0.08	H	53	+	2.31	CTTAACTCAATGTGC	Aconitate hydratase 1
*so0383-2 (hsdSM)*	-0.64	-0.59	J	276	+	3.04	GTTAATAAAATGTTT	Type I restriction-modification system
*so0756 (aroG)*	-0.84	-2.18	A	164	+	2.74	TTTAAAAATATGTTA	Phospho-2-dehydro-3-deoxyheptonate aldolase, phe-sensitive
*so0806*	3.22	3.64	E	45	-	2.49	GAAAATTTTTTGTTA	Alkaline phosphatase, putative
*so0866*	7.00	8.08	L	136	+	2.94	GTAATTTAAATGTTA	Serine protease, subtilase family
*so0916*	2.19	1.10	O	150	+	2.35	GTTAATAAAATATTG	Transcriptional regulator, marr family
*so1004-3*	-1.31	-2.39	J	199	-	2.65	GTTATTGAAATGTAA	Hypothetical protein
*so1035-9 (cobTSUQO)*	0.36	1.50	B	83	+	2.56	GTTAATTTAATGCTT	Nicotinate-nucleotide-dimethylbenzimidazole phosphoribosyltransferase
*so1307 (aqpZ)*	1.42	0.87	R	197	-	3.01	GTTAACAAAGCGATA	Aquaporin Z
*so1309*	-0.09	1.20	J	161	+	3.17	GTTACTTAAATGTTA	Conserved hypothetical protein
*so1427-30*	-1.77	-4.37	C	246	-	3.13	GTTAATAAAATGTTT	Decaheme cytochrome c
***so1483 (aceBA)***	3.90	0.15	H	291	+	2.34	TTTCACTAGATGTTA	Malate synthase A
***so1623 (ptsG)***	2.00	3.26	P	506	-	2.76	GTTACTTTATTGTTA	PTS system, glucose-specific IIBC component
*so1659*	0.83	1.54	H	260	-	2.49	ATTAATTTAATGATA	Decaheme cytochrome c
*so1661*	2.47	3.11	O	219	+	3.12	GTTAAATAATTGTTA	Transcriptional regulator, lysr family
*so1673*	0.64	3.69	C	288	+	2.66	GTAAATGAAATGTAA	Outer membrane protein ompw, putative
***so1806 (pspF)***	2.63	2.42	O	90	-	3.13	GTTAATAAAATGTTT	Psp operon transcriptional activator
*so1807-9 (pspABC)*	-1.49	-0.85	L	102	+	3.13	GTTAATAAAATGTTT	Phage shock protein A
*so1812 (mdeA)*	3.10	3.46	H	84	+	2.45	GTTATTTAAAAGATA	Methionine gamma-lyase
*so1821*	-2.29	-3.22	R	230	+	3.05	GTTAATTTGATGTTA	Outer membrane porin, putative
*so1822*	3.35	4.99	R	133	+	2.41	GTTGATTAAAGGTTT	Tonb-dependent receptor, putative
*so1944*	1.12	1.80	J	65	-	2.96	GTTAAGTAATTGTAA	Hypothetical protein
*so1961 (maa)*	2.96	-1.57	D	114	+	2.63	GTTAGCTAAATGGTA	Maltose O-acetyltransferase
*so2099-7*	4.09	2.40	H	147	+	3.29	GTTAATTAAATGTCA	Quinone-reactive Ni/Fe hydrogenase, small subunit precursor
*so2100*	1.18	0.63	J	216	-	3.29	GTTAATTAAATGTCA	Thioredoxin family protein
*so2199*	1.48	3.72	J	101	-	2.76	GTGAATAAAATGTTT	Hypothetical protein
*so2305 (lrp)*	-1.40	-0.02	O	507	+	2.91	TTTAATTAAATCTTC	Leucine-responsive regulatory protein
*so2402 (rpsA)*	-1.03	-0.20	M	21	-	2.56	GTTACTTTAATGTAA	Ribosomal protein S1
*so2483*	-2.10	-3.82	A	143	-	2.34	TTTAACTAAGTGTTA	Aspartate aminotransferase, putative
*so2706 (astB)*	0.96	1.95	H	209	+	2.87	GTTGAAAAAATGTAA	Succinylarginine dihydrolase
*so2727*	-0.97	-1.47	H	220	-	2.79	GTTATTCAAATGTAA	Cytochrome c3
*so2858-7*	1.04	-2.18	J	153	+	3.07	GTAAATTCAATGTTA	Conserved hypothetical protein
*so2907*	-2.41	0.53	T	179	-	3.13	GTTAATAAAATGTTT	Tonb-dependent receptor domain protein
*so3099*	-5.82	-4.70	R	281	-	2.96	GTTAATTAAATTATA	Long-chain fatty acid transport protein, putative
*so3106 (aprE)*	5.45	5.94	L	127	-	3.12	GTAAATTAATTGTTA	Cold-active serine alkaline protease
*so3278-9*	2.39	2.87	J	41	-	2.59	GTTAATTTTATGTAA	Conserved hypothetical protein
*so3395-4*	2.15	4.06	J	14	+	2.41	GTGAGTTAAAGGTTA	Hypothetical protein
*so3480*	1.90	2.92	J	310	+	2.84	TTTAATTAAAATTTA	Conserved hypothetical protein
*so3489*	2.42	2.56	T	41	+	2.92	ATCAATTAAATGTTA	GGDEF domain protein
*so3507*	-0.57	0.70	J	90	-	3.02	GTTAACTCAATGTTA	Conserved hypothetical protein
*so3508*	-2.79	0.05	J	46	+	3.02	GTTAACTCAATGTTA	Hypothetical protein
*so3564 (dcp-2)*	0.02	0.67	O	441	+	3.04	GTTAATTAATTGTTG	Peptidyl-dipeptidase Dcp
*so3565 (cpdB)*	-1.87	2.10	H	257	-	3.04	GTTAATTAATTGTTG	2,3-cyclic-nucleotide 2-phosphodiesterase
*so3855 (sfcA)*	0.36	0.49	H	177	+	2.93	GTTAATTGATTGTAA	Malate oxidoreductase
*so3863-5 (modABC)*	0.53	2.49	R	206	+	2.75	CTTGAGTAAATGTTA	Molybdenum ABC transporter, periplasmic molybdenum-binding protein
*so4245 (argA)*	0.19	0.41	A	178	-	2.65	GTTAAAAAAATGTGA	Amino-acid acetyltransferase
*so4273*	-0.85	0.39	L	9	+	3.08	GGTAATTAATTGTTA	Hypothetical protein
*so4457*	1.69	2.66	T	63	-	2.76	GTTGCCTAAATGTTA	GGDEF domain protein
***so4480 (aldA)***	0.87	-0.47	H	562	+	2.73	GTTAAATAAAGGTAA	Aldehyde dehydrogenase
*so4570*	-0.33	-3.76	J	147	-	3.13	GTTAATAAAATGTTT	Conserved domain protein
*so4591 (cymA)*	-0.40	-1.65	H	112	-	2.64	ATTAATTAAAACTTA	Tetraheme cytochrome c
*so4592*	2.45	1.40	J	312	+	2.64	ATTAATTAAAACTTA	Hypothetical protein

*a *In bold, common in ArcA regulons of *E. coli *and *S. oneidensis*.

^b ^Ratio of expression: *arcA*^-^/MR-1.

^c ^Functional category, referring to Figure 2.

^d ^location of the binding sequence is in bp upstream of the start codon.

^e ^strand.

^f ^z score.

## Discussion

The canonical Arc signal transduction system in γ-proteobacterial species typified by *E. coli *is recognized as a second global regulator that, like Fnr, mediates gene expression in response to respiratory condition changes [7,8,13]. In *S. oneidensis*, EtrA, an analog of *E. coli *Fnr, appears to play a negligible role in this regulatory process, leaving the Arc system the best currently recognized candidate for the role [3,4]. In addition to high similarities in protein sequence and structure, the *S. oneidensis arcA *gene has been shown to be able to complement an *E. coli arcA *deletion mutant [14]. These findings strongly suggest a functional similarity between ArcA proteins in *S. oneidensis *and in other organisms and that the Asp^54 ^residue is the phosphorylation site if required. A homolog to the *arcB *gene in *S. oneidensis *MR-1 has yet to be identified in the genome. Further comparative analyses of multiple Alteromonadaceae genomes indicate that this type of Arc system is in fact common among the Alteromonadaceae. Unfortunately, the 'atyptical' Arc systems have been largely overlooked possibly because of the sequence and structure conservation of ArcAs.

In this study, we have attempted to understand the major physiological changes mediated by ArcA and define its regulon with a comparison with ArcA of the canonical Arc system in *E. coli*. The two systems differ significantly from each other in several key aspects. Firstly, physiologically, one of major questions about the Arc system is whether it regulates any aspect of aerobic respiration. Unexpectedly, *S. oneidensis *ArcA is directly involved in aerobic metabolism. In *E. coli*, it is believed that the Arc system regulates gene expression in response to anaerobic conditions under which ArcB phosphorylates ArcA [8]. Correspondingly, the maximum growth rate of an *E. coli arcA *mutant was not significantly different from that of the wild-type when grown on a variety of media if oxygen deprivation is excluded [22,30,63-65]. Similarly, the absence of ArcA did not show any effect on cell morphology and growth characteristics of *Salmonella enterica *serovar Enteritidis under aerobic conditions [15]. All these results indicate that ArcA has a very limited role, if any, in aerobic respiration in these bacteria. Secondly, ArcA of *S. oneidensis *appears to be irrelevant to survival during stationary phase. The *E. coli *ArcA is heavily involved in starvation-induced modulations of gene expression and therefore plays a key role in the bacterial stasis survival [29,66,67]. Thirdly, *E. coli *ArcA proteins directly control the TCA cycle while the *S. oneidensis *ArcA controls DMSO reduction directly [14]. All these differences suggest that the *S. oneidensis *ArcA functionally deviates from the canonical one considerably.

**Figure 7:**
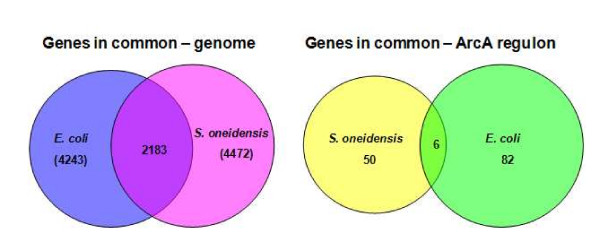
**Common genes in *S. oneidensis *and *E. coli *genomes and in ArcA regulons of these two bacteria**. Common genes in *S. oneidensis *and *E. coli *genomes are obtained from [68] using default similarity cutoffs (maximum E-value, 1e-5; minimum percent identity, 30).

In spite of significant difference in their physiological roles, the activation mechanism by phosphorylation and the target sequences of ArcA proteins of *E. coli *and *S. oneidensis *share a high level of similarity. In this study, the EMSA results reinstated that a binding motif in the promoter region rather than expression differences of target genes appears to be more crucial for binding. Nevertheless, a combination of binding motif and expression difference promotes more accurate prediction. With the combination, up to 50 *S. oneidensis *operons are identified while at least 82 operons are reportedly under direct control of ArcA in *E. coli *[12]. Given that up to 2183 genes are predicted to be in common in *E. coli *(~48.8%) and *S. oneidensis *(~51.4%) genomes [68], the number of overlapping operons (6) is surprisingly small (Figure 7). The majority of members in the *E. coli *ArcA regulon involved in metabolism are not identified in the *S. oneidensis *ArcA regulon, implicating that a significant difference in ArcA regulons of these two organisms has evolved. It is worth noting that none of the operons encoding TCA enzymes were located next to these high-confident ArcA-binding sites, consistent with our observation that these TCA operons were not affected by the *arcA *mutation. In *E. coli*, on the contrary, the promoter region of most TCA genes contains an ArcA-binding site with *z *score above 3.0 [12]. It is possible that ArcA-independent expression of *S. oneidensis *TCA genes may be largely due to the loss of ArcA-binding sites in their promoter regions.

Similarly, *S. oneidensis *ArcA may acquire controls over new genes once an ArcA-binding site emerged in their promoter regions through evolution. Genes (*so1427-30*) for DMSO reductase and related proteins serve as a good example. DMSO reduction pathway shares most of components for anaerobic respiration on all other electron acceptors except TMAO [46]. In *E. coli*, the Arc system functions as a global regulator of respiratory gene expression under microaerobic and anaerobic growth conditions [8-10]. As a result, utilization of many compounds anaerobically is found to be affected simultaneously. In contrast, the *arcA *mutant is defective in DMSO respiration only, making it hard to accept that *S. oneidensis *ArcA functions in a similar way. Given that a predicted ArcA-binding site is found within *so1427 *promoter region, we propose that this operon become a new number of ArcA regulon only because the binding site shows up, presumably by chance.

## Conclusion

This study provides the first comprehensive profile to elucidate the functions of the atypical Arc system in *S. oneidensis*, compared to the canonical one in *E. coli*. Our findings demonstrated that two Arc systems are significantly different from each other with respect to the physiological functions and the regulons although the sequences and binding motif are highly similar. *S. oneidensis *ArcA does not appear to be involved in regulation of TCA cycle while ArcA in *E. coli *repress the genes involved in the TCA cycle under anaerobic condition. More than 50 operons were confidently identified as members of the *Shewanella *ArcA regulon, but a much larger number of members are expected. However, only a very limited number of the regulon members are shared by the *E. coli *ArcA regulon. The significant differences in both physiology of *arcA *mutants and regulon of ArcA proteins of these two microorganisms may simply be due to the differences in lifestyle, metabolism, and gene content between them. Further molecular characterization of the lineage-specific ArcA regulon members identified in this study is needed to dissect the functional diversity and ultimately the evolution of the Arc system in γ-proteobacteria.

## Methods

### Bacterial strains, plasmids, and culture conditions

A list of all bacterial strains and plasmids used in this study is given in Table 3. *E. coli *and *S. oneidensis *strains under aerobic conditions were grown in Luria-Bertani (LB, Difco, Detroit, MI) medium at 37°C and room temperature for genetic manipulation, respectively. When needed, the growth medium was supplemented with antibiotics at the following concentrations: ampicillin at 50 μg/ml and gentamycin at 15 μg/ml. The suicide vector pDS3.1 has been described elsewhere [20].

**Table 3:** Strains and plasmids used in this study

Strain or plasmid	Description	Reference or source
Strain or plasmid	Description	Reference or source
BL21	F^- ^*omp*T *hsd*S_B_(r_B_^-^m_B_^-^) *gal dcm *(DE3)	Invitrogen
WM3064	Donor strain for conjugation; Δ*dapA*	68
		
*S. oneidensis *strains		
MR-1	Wild-type	Lab stock
JZ3988K	*arcA *deletion mutant derived from MR-1; Δ*arcA*	This study
JZ3988K-CEM	JZ3988K with pBBR1MCS-5	This study
JZ3988K-COM	JZ3988K with pBBR-ARCA	This study
		
Plasmids		
pDS3.1	Ap^r^, Gm^r^, derivative from suicide vector pCVD442	20
pDS-ARCAK	pDS3.1 containing the PCR fragment for deleting *arcA*	This study
pBBR1MCS-5	Gm^r ^vector used for complementation,	69
pBBR-ARCA	pBBR1MCS-5 containing *arcA *and upstream promoter region from MR-1	This study
pDONR221	Entry vector of the Gateway system	Invitrogen
pDEST17	Destination vector of the Gateway system (His-tag)	Invitrogen

### Disruption of *arcA *and complementation of the resulting *arcA *mutant

An *arcA *deletion strain was constructed. Primers used for generating PCR products for mutagenesis are listed in Table S4 (Table S4 in additional file 5). In brief, two fragments flanking *arcA *were amplified by PCR with primers SO3988-5-F and SO3988-5-R, primers SO3988-3-F and SO3988-3-R, respectively, and purified using the QIAquick PCR purification kit (Qiagen, Chatsworth, CA). Fusion PCR products generated using the amplified fragments as templates with primers SO3988-5-F and SO3988-3-R as described elsewhere [69]. The resulting fusion fragment was ligated into the *Xcm*I site of plasmid pDS3.1 and the resulting mutagenesis vectors (pDS-ARCAK) were transformed into the plasmid donor strain, *E. coli *WM3064 [70]. Plasmids pDS-ARCAK in WM3064, grown on LB supplemented with 0.3 mM diaminopimelic acid (DAP), were further transferred to MR-1 by conjugation [20]. Integration of the mutagenesis construct into the chromosome was selected by gentamycin resistance and confirmed by PCR amplification. Verified transconjugants were grown in LB broth in the absence of NaCl and plated on LB supplemented with 10% of sucrose. Gentamycin-sensitive and sucrose-resistant colonies were screened by PCR for the deletion of *arcA*. The deletion mutation was then verified by sequencing of the mutated region, and the deletion strain was designated as JZ3988K (*ΔarcA*).

For complementation, a 1.4-kb DNA fragment containing *arcA *and its native promoter was generated by PCR amplification with MR-1 genomic DNA as the template using primers SO3988-COM-F and SO3988-COM-R as listed in Table S4 (Table S4 in additional file 5). This fragment was digested with *Sac*I (underlined) and ligated to *Sac*I-digested pBBR1MCS-5 to form pBBR-ARCA [71], which was electroporated into WM3064. Introduction of pBBR-ARCA into JZ3988K was done by mating with WM3064 hosting pBBR-ARCA, and gentamycin-resistant colonies were selected. The presence of pBBR-ARCA in JZ3988K was confirmed by plasmid purification and restriction enzyme digestion.

### Physiological characterization of the mutation strain under various conditions

M1 defined medium containing 0.02% (w/v) of vitamin-free Casamino Acids and 15 mM lactate was used in all physiological experiments [72]. Growth of the deletion strain under aerobic or anaerobic conditions was determined by recording growth curves in triplicate with a Bioscreen C microbiology reader (Labsystems Oy, Helsinki, Finland) with MR-1 as the control. For aerobic growth, exponential phase cultures were diluted to approximately ~1 × 10^5 ^cells/ml in fresh medium, and 400 μl was transferred to the honeycomb plate wells of the Bioscreen C reader. The cultures were shaken at medium intensity continuously, and the turbidity was measured every 30 min at 600 nm and DO (dissolved oxygen) was recorded every hour with an Accumet XL40 meter (Fisher Scientific). For anaerobic growth, exponential phase cultures grown aerobically were centrifuged, purged in nitrogen and suspended in fresh medium to approximately ~1 × 10^5 ^cells/ml in an anaerobic glove box. Electron acceptors tested in this study included fumarate (20 mM), nitrate (2 mM), nitrite (1 mM), thiosulfate (3 mM), TMAO (20 mM), and DMSO (20 mM). For electron acceptors containing metals including MnO_2 _(5 mM), ferric citrate (10 mM), and cobalt(III)-EDTA (200 μM), growth was monitored by the color change of the cultures and cell counting under a microscope (Nikon Optiphot, Nikon, Japan).

Survival of MR-1 and the *ΔarcA *strain during the stationary phase was examined. Cultures were grown from a single colony under aerobic conditions with vigorous shaking. After the onset of stationary phase, the cultures were divided into two parts. One was kept in the incubator with vigorous shaking and the other was kept still. The cultures were serially diluted into LB and plated onto LB plates every 12 h. Plates from dilutions that gave 100 to 250 colony form units (CFU) per plate were used to minimize statistical variation due to small sample sizes. Experiments were done in triplicate.

### Microarray analysis

For each strain under aerobic conditions, 100 ml of M1 medium in a 500 ml shake flask was inoculated with fresh overnight culture to OD_600 _of 0.01 and then was divided into four aliquots (biological replicates) and shaken on a rotary platform (250 rpm) until mid-log phase (OD ≈ 0.4 at 600 nm). For anaerobic cultures, 500 ml of M1 supplemented with 10 mM fumarate as the electron acceptor was inoculated to an OD_600 _of 0.01 and then divided into four aliquots (biological replicates) and kept in an anaerobic chamber until mid-log phase (OD ≈ 0.15 at 600 nm). All cultures were centrifuged at 8000 rpm in a Sorvall RC5C plus for 3 min at the room temperature and the pellet was frozen immediately in liquid nitrogen and stored at -80°C.

DNA microarrays were constructed using PCR-amplified fragments of each annotated open reading frame from *S. oneidensis *MR-1, as previously described [21]. Total RNA extraction, cDNA labeling, hybridization, and slide scanning were conducted according to the standard procedure used in our lab [21,73,74]. LOWESS was used to normalize the data set which subsequently was subjected to statistical analysis by analysis of variance (ANOVA) with Benjamini and Hochberg False Discovery Rate as multiple testing correction. Genes with at an ANOVA *P *value of < 0.05 were considered significantly differentially expressed and were listed in supplemental materials. For discussion in text, a list of genes with at least a 2-fold change in expression and an ANOVA *P *value of < 0.01 were used. All raw data (MIAME) have been deposited to NCBI GEO (GSE7973).

### Real-time quantitative RT-PCR (qRT-PCR)

qRT-PCR was performed with primers listed in Table S4 (Table S4 in additional file 5) as described previously [21,74]. A 100-bp fragment of the *acnA *gene, which was amplified by PCR with genomic DNA as the template, was used to construct the standard curve. The expression of each gene was determined from three replicates on a single real-time qRT-PCR experiment. The expression ratio was recorded as the fold difference in quantity of real-time qRT-PCR product from samples grown at the treatment vs. control.

### Expression and purification of *S. oneidensis *ArcA protein

Plasmids pDONR221 and pDEST17 and *E. coli *BL21 (DE3) Star cells were obtained from Invitrogen. To create pDEST17-ArcA, the ArcA encoding ORF was first cloned into pDONR221 by using ArcA-up/down primers (Table S4 in additional file 5), and then transferred into pDEST17 for protein expression by Gateway recombination reactions. All of these plasmid constructs were verified by DNA sequencing. The expression of ArcA in *E. coli *BL21(DE3) Star cells was induced with 0.5 mM IPTG from mid-log phase (OD_600 _= 0.5–0.6) at 30°C. The cells were grown to saturation and then collected by centrifugation, resuspended in lysis buffer (50 mM Tris/HCl, pH 7.5, 200 mM NaCl, 1 mM MgCl_2_, 10 mM β-mercaptoethanol, 1 mM PMSF, 5 μg/mL DNaseI), and broken by passage twice through a French press (10,000 psi). The resulting inclusion body pellets were solubilized with 20 mM Tris/HCl (pH 8.0), 5 M urea and 100 mM NaCl, and the ArcA protein was further purified by using Talon resin columns (BD Biosciences^®^) under denaturing conditions according to manufacturer instructions. To renature the protein, the eluted fractions containing ArcA protein were collected, diluted into 0.8 M urea, 20 mM Tris/HCl (pH 8.0), 1 mM EDTA by sequential dilutions, and then dialyzed against 20 mM Tris/HCl (pH 7.5). Finally the ArcA protein was concentrated to ~0.6 mg/ml.

### Phosphorylation of ArcA and electrophoretic motility shift assay (EMSA)

Phosphorylation of purified ArcA protein was performed in buffer containing 100 mM Tris/HCl (pH 7.0), 10 mM MgCl_2_, 125 mM KCl, 50 mM dilithium carbamoyl phosphate for 60 minutes at room temperature as described [7]. The probes used for EMSA were prepared by PCR with ^33^P end-labeled primers (Table S4 in additional file 5). The binding reaction was performed with ~25–50 fmol (~2–5 nM) labeled probes and various amount of protein in 12 μl binding buffer containing 100 mM Tris/HCl (pH 7.4), 20 mM KCl, 10 mM MgCl_2_, 2 mM DTT, 0.2 μg/μl poly(dI·dC), and 10% glycerol at 15°C for 60 minutes and resolved on pre-run 4.8% polyacrylamide native gels [7]. The band shifts were visualized by autoradiography.

### ArcA weight matrix development and identification of putative ArcA-binding sites

AlignACE was used to screen for a common ArcA-binding motif within promoter regions of ArcA-controlled operons predicted either by DNA footprinting or microarray analysis [58]. The identified ArcA-binding motifs of 15 bp were transformed to a weight matrix using the method of Berg and von Hippel [62]. The whole genome was then scaned for putative ArcA-binding motifs with the weight matrix.

## Authors' contributions

HG carried out mutant construction and characterization, microarray analysis, weight matrix construction and screening, and drafted the manuscript. XW performed protein expression and purification, microarray analysis, and drafted the manuscript. ZKY participated in mutant construction and characterization, and microarray analysis. TP oversaw the protein work. JZ supervised all parts of the study. All authors read and approved the final manuscript.

## Supplementary Material

Additional file 1 Comparison of expression measurements by microarray and Real Time qRT-PCR assays. The data provided represent the validation of microarray data by qRT-PCR assays. Click here for file

Additional file 2 Genes that exhibit significant changes in the *ΔarcA *strain. The data provided represent all genes whose expression was altered by arcA mutation. Click here for file

Additional file 3 Genes that exhibit significant changes in the *ΔarcA *strain (for discussion). The data provided represent all genes listed in additional file 2 that were discussed in details. Click here for file

Additional file 4 Genes in operons whose upstream regions contain an ArcA binding site. The data provided represent all operons whose upstream region contains a putative ArcA binding site. Click here for file

Additional file 5 Primers used in this study. The data provided represent all primers used in this study. Click here for file
