# Supplementary material for: Probing regulon of ArcA in Shewanella oneidensis MR-1 by integrated genomic analyses
Source: BMC Genomics. 2008 Jan 25;9:42. doi: 10.1186/1471-2164-9-42 (PMC2262068; doi:10.1186/1471-2164-9-42)
Supplement: Additional file 1 — Comparison of expression measurements by microarray and Real Time qRT-PCR assays. The data provided represent the validation of microarray data by qRT-PCR assays. [file 1471-2164-9-42-S1.ppt]

## Slide 1
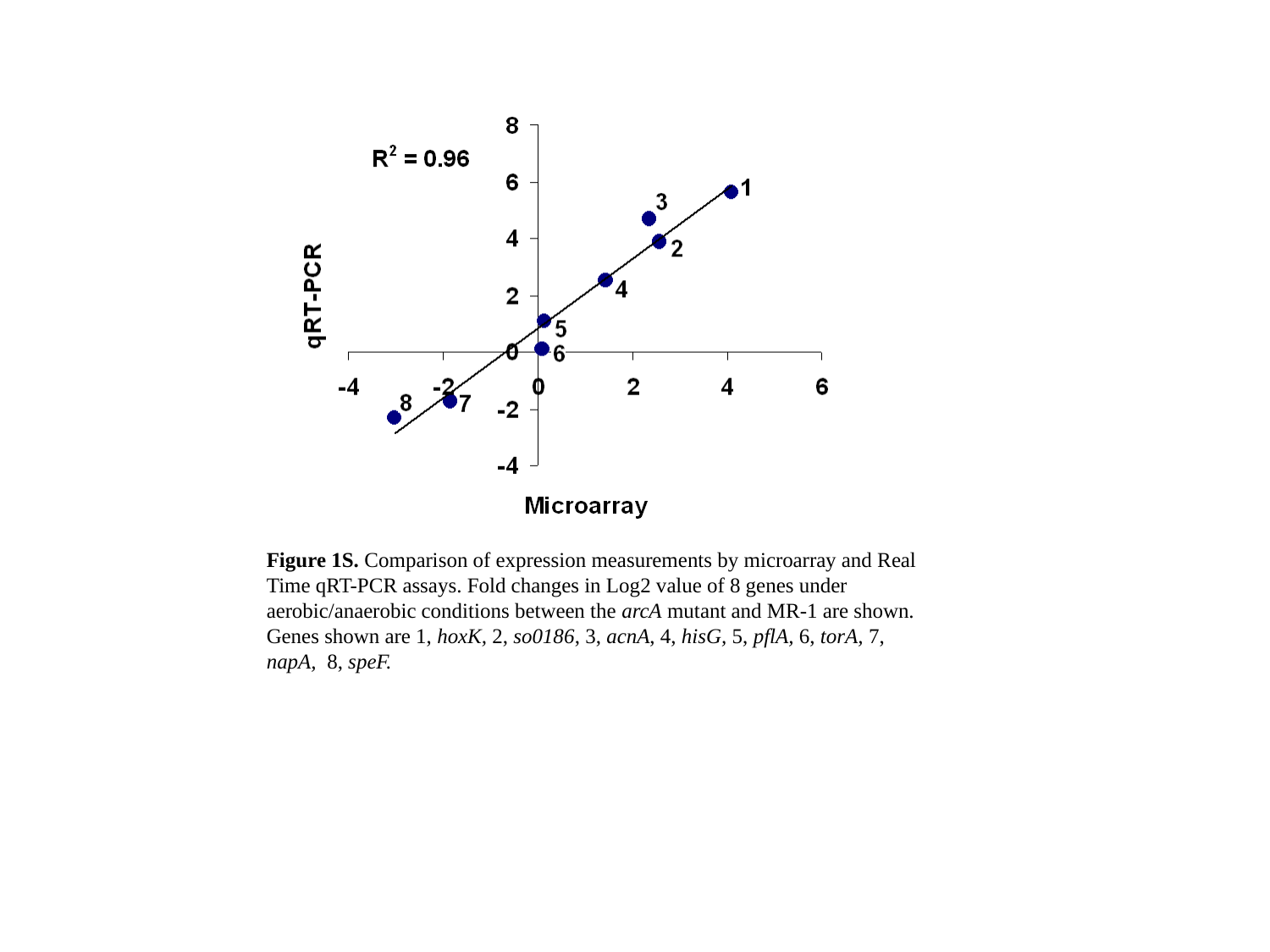

Figure 1S. Comparison of expression measurements by microarray and Real Time qRT-PCR assays. Fold changes in Log2 value of 8 genes under aerobic/anaerobic conditions between the arcA mutant and MR-1 are shown. Genes shown are 1, hoxK, 2, so0186, 3, acnA, 4, hisG, 5, pflA, 6, torA, 7, napA, 8, speF.
