# Supplementary material for: Probing regulon of ArcA in Shewanella oneidensis MR-1 by integrated genomic analyses
Source: BMC Genomics. 2008 Jan 25;9:42. doi: 10.1186/1471-2164-9-42 (PMC2262068; doi:10.1186/1471-2164-9-42)
Supplement: Additional file 2 — Genes that exhibit significant changes in the ΔarcA strain. The data provided represent all genes whose expression was altered by arcA mutation. [file 1471-2164-9-42-S2.doc]

| **TABLE S1. Genes that exhibit significant changes in the *∆arcA* strain** | | | | |
| --- | --- | --- | --- | --- |
| Gene | Ratio (log2)  +O_2_ -O_2_ | | FC^a^ | Function |
| *so0002* | -1.80 | -1.38 | R | proton/peptide symporter family protein |
| *so0004* | -1.36 | -0.60 | C | inner membrane protein, 60 kDa |
| *so0005* | -1.24 | -0.37 | J | conserved hypothetical protein TIGR00278 |
| *so0006* | -1.10 | -0.37 | Q | ribonuclease P protein component |
| *so0017* | 1.94 | 3.06 | J | conserved hypothetical protein |
| *so0018* | -1.07 | -1.46 | J | conserved hypothetical protein |
| *so0020* | 0.89 | -2.33 | I | fatty oxidation complex, beta subunit |
| *so0021* | 0.67 | -1.81 | I | fatty oxidation complex, alpha subunit |
| *so0050* | -1.24 | 0.16 | T | rhodanese domain protein |
| *so0053* | -0.38 | 0.49 | H | glycerol-3-phosphate dehydrogenase (NAD(P)+) |
| *so0075* | -0.57 | -1.43 | T | AMP-binding family protein |
| *so0076* | -1.98 | -1.89 | J | hypothetical protein |
| *so0077* | -0.93 | -1.27 | J | conserved hypothetical protein |
| *so0078* | 0.16 | -1.03 | J | hypothetical protein |
| *so0079* | -0.30 | -1.30 | J | conserved hypothetical protein |
| *so0080* | -0.24 | -1.00 | J | conserved hypothetical protein |
| *so0090* | 1.52 | 3.42 | J | hypothetical protein |
| *so0091* | -1.12 | -1.57 | J | hypothetical protein |
| *so0092* | 0.02 | -2.43 | N | purine nucleoside phosphorylase |
| *so0095* | -0.28 | 0.78 | H | imidazolonepropionase |
| *so0097* | -0.44 | 1.04 | H | urocanate hydratase |
| *so0098* | -0.55 | 0.71 | H | histidine ammonia-lyase |
| *so0100* | -0.05 | -1.15 | J | hypothetical protein |
| *so0101* | -1.26 | 0.62 | H | formate dehydrogenase, nitrate inducible, alpha subunit, selenocysteine-containing |
| *so0102* | -1.17 | 1.37 | H | formate dehydrogenase, nitrate-inducible, iron-sulfur subunit |
| *so0104* | -0.95 | 0.26 | H | fdhE protein |
| *so0106* | 0.13 | 0.85 | M | selenocysteine-specific translation elongation factor |
| *so0109* | -0.94 | -0.42 | J | conserved hypothetical protein |
| *so0112* | 0.86 | 1.87 | J | conserved hypothetical protein |
| *so0115* | -1.76 | 1.32 | J | hypothetical protein |
| *so0120* | -1.25 | -2.51 | J | hypothetical protein |
| *so0129* | 0.44 | 2.19 | J | conserved hypothetical protein TIGR00427 |
| *so0130* | -0.03 | 1.68 | L | protease, putative |
| *so0139* | -2.02 | -0.52 | R | ferritin |
| *so0142* | 0.06 | -2.58 | B | 3,4-dihydroxy-2-butanone 4-phosphate synthase |
| *so0148* | -0.69 | -1.72 | J | hypothetical protein |
| *so0149* | 0.43 | -1.44 | J | hypothetical protein |
| *so0166* | 1.40 | 0.67 | L | general secretion pathway protein D |
| *so0172* | 0.72 | 0.53 | L | general secretion pathway protein J |
| *so0173* | 0.73 | 0.53 | L | general secretion pathway protein K |
| *so0175* | 1.18 | 0.75 | L | general secretion pathway protein M |
| *so0182* | 1.48 | -0.79 | J | hypothetical protein |
| *so0183* | 0.47 | 1.42 | T | acetyltransferase, GNAT family |
| *so0184* | 2.12 | -0.46 | J | conserved hypothetical protein |
| *so0185* | 1.42 | 1.53 | J | conserved hypothetical protein |
| *so0186* | 2.57 | 0.37 | J | conserved hypothetical protein |
| *so0187* | 1.40 | 0.15 | L | serine protease, subtilase family |
| *so0188* | 1.10 | -0.22 | J | hypothetical protein |
| *so0189* | 2.33 | 0.27 | T | fibronectin type III domain protein |
| *so0206* | -1.02 | -0.13 | M | tRNA (uracil-5-)-methyltransferase |
| *so0208* | 0.23 | 1.39 | Q | RNA-binding protein |
| *so0214* | 0.35 | 0.98 | O | birA bifunctional protein |
| *so0220* | -1.36 | 0.03 | M | ribosomal protein L11 |
| *so0221* | -1.42 | -0.09 | M | ribosomal protein L1 |
| *so0222* | -1.21 | -0.26 | M | ribosomal protein L10 |
| *so0223* | -1.24 | -0.60 | M | ribosomal protein L7/L12 |
| *so0226* | -1.46 | -0.34 | M | ribosomal protein S12 |
| *so0227* | -1.22 | -0.31 | M | ribosomal protein S7 |
| *so0228* | -1.22 | -0.11 | M | translation elongation factor G |
| *so0230* | -1.26 | 0.53 | M | ribosomal protein S10 |
| *so0231* | -1.03 | 0.46 | M | ribosomal protein L3 |
| *so0232* | -0.10 | 1.18 | M | ribosomal protein L4 |
| *so0233* | -0.90 | 0.61 | M | ribosomal protein L23 |
| *so0241* | -1.19 | -0.03 | M | ribosomal protein L14 |
| *so0242* | -1.30 | -0.14 | M | ribosomal protein L24 |
| *so0243* | -1.43 | -0.33 | M | ribosomal protein L5 |
| *so0244* | -0.93 | 0.02 | M | ribosomal protein S14 |
| *so0248* | -0.99 | -0.01 | M | ribosomal protein S5 |
| *so0249* | -0.93 | -0.08 | M | ribosomal protein L30 |
| *so0251* | -1.14 | 0.15 | L | preprotein translocase, SecY subunit |
| *so0252* | -1.18 | -0.31 | M | ribosomal protein L36 |
| *so0262* | 0.88 | -0.02 | R | heme exporter protein CcmB |
| *so0264* | -1.23 | -1.31 | H | cytochrome c |
| *so0266* | 1.07 | 0.94 | H | cytochrome c-type biogenesis protein CcmF |
| *so0268* | 0.68 | 0.75 | H | cytochrome c-type biogenesis protein CcmH |
| *so0269* | 0.82 | 0.99 | H | thioredoxin, putative |
| *so0276* | 1.13 | 0.24 | A | acetylglutamate kinase |
| *so0279* | 2.16 | 1.26 | A | argininosuccinate lyase |
| *so0281* | 0.44 | 1.26 | C | type IV pilus biogenesis protein PilM |
| *so0291* | 0.32 | 1.53 | J | hypothetical protein |
| *so0306* | 3.46 | 2.54 | J | hypothetical protein |
| *so0308* | 1.53 | 2.22 | J | conserved hypothetical protein |
| *so0310* | 0.85 | 0.94 | J | hypothetical protein |
| *so0312* | -0.16 | -1.42 | R | outer membrane porin, putative |
| *so0314* | -3.03 | -2.79 | E | ornithine decarboxylase, inducible |
| *so0321* | 0.51 | 0.73 | J | hypothetical protein |
| *so0322* | 1.04 | 1.85 | J | hypothetical protein |
| *so0336* | 1.56 | 1.18 | J | conserved hypothetical protein |
| *so0342* | 1.90 | 0.20 | J | conserved hypothetical protein |
| *so0343* | 2.35 | 0.08 | H | aconitate hydratase 1 |
| *so0344* | 2.01 | 0.42 | H | methylcitrate synthase |
| *so0345* | 2.02 | 0.51 | H | methylisocitrate lyase |
| *so0346* | 1.10 | 0.21 | O | transcriptional regulator. GntR family |
| *so0354* | -1.10 | -0.92 | R | sodium/calcium exchanger |
| *so0370* | -1.94 | -2.48 | J | conserved hypothetical protein |
| *so0380* | -0.93 | -0.83 | G | type I restriction-modification system, R subunit |
| *so0381* | -0.74 | -0.83 | J | death-on-curing family protein |
| *so0382* | -1.41 | -1.09 | G | type I restriction-modification system, S subunit |
| *so0392* | -0.30 | -1.41 | C | lipoprotein, putative |
| *so0393* | -1.32 | 0.20 | O | DNA-binding protein Fis |
| *so0398* | 1.40 | 0.21 | H | fumarate reductase flavoprotein subunit |
| *so0403* | -3.01 | -4.09 | J | hypothetical protein |
| *so0404* | -4.68 | -4.89 | J | hypothetical protein |
| *so0406* | -1.22 | -0.47 | H | thioredoxin 1 |
| *so0410* | -1.25 | -0.19 | G | mutator mutT protein |
| *so0412* | 0.96 | 0.25 | J | conserved hypothetical protein |
| *so0416* | 0.52 | 1.05 | C | type IV pilus biogenesis protein PilB |
| *so0427* | 0.88 | 1.26 | O | sensory box protein |
| *so0430* | -1.16 | -1.19 | J | conserved hypothetical protein |
| *so0431* | -0.95 | -1.02 | T | HAD-superfamily hydrolase, subfamily IA, variant 3 protein family |
| *so0433* | 0.12 | -1.06 | O | regulator of sigma D |
| *so0440* | 1.27 | 0.92 | J | conserved hypothetical protein |
| *so0441* | 0.48 | -1.29 | N | phosphoribosylamine--glycine ligase |
| *so0442* | 0.12 | -1.14 | N | phosphoribosylaminoimidazolecarboxamide formyltransferase/IMP cyclohydrolase |
| *so0443* | -1.10 | -0.45 | O | transcriptional regulator, MerR family |
| *so0445* | 1.42 | 2.05 | L | hflC protein, putative |
| *so0450* | 0.18 | -1.13 | R | major facilitator family protein |
| *so0459* | 0.96 | 0.11 | J | conserved hypothetical protein |
| *so0461* | 0.09 | 1.12 | J | hypothetical protein |
| *so0463* | 1.03 | 0.92 | J | conserved hypothetical protein |
| *so0470* | -1.46 | -0.47 | J | hypothetical protein |
| *so0492* | -0.73 | -0.46 | J | conserved hypothetical protein |
| *so0493* | -0.29 | -1.09 | J | hypothetical protein |
| *so0499* | -1.00 | -0.81 | J | hypothetical protein |
| *so0500* | 1.30 | 0.06 | D | methyl-accepting chemotaxis protein |
| *so0501* | -0.66 | -0.84 | J | conserved hypothetical protein |
| *so0516* | 1.10 | 0.08 | J | hypothetical protein |
| *so0521* | -0.66 | -1.16 | J | conserved hypothetical protein |
| *so0523* | -0.91 | -0.43 | O | transcriptional regulator, LysR family |
| *so0538* | 1.22 | 0.02 | H | glyceraldehyde 3-phosphate dehydrogenase |
| *so0541* | 1.00 | 0.09 | T | metallo-beta-lactamase family protein |
| *so0549* | -1.07 | -0.24 | D | chemotaxis protein CheY/response regulator receiver domain protein |
| *so0552* | 1.16 | 2.11 | J | hypothetical protein |
| *so0553* | 1.68 | 3.42 | J | hypothetical protein |
| *so0554* | 0.30 | 1.41 | J | hypothetical protein |
| *so0556* | -1.16 | -0.56 | J | hypothetical protein |
| *so0558* | -1.04 | -0.55 | T | smtA protein |
| *so0564* | 1.07 | 1.41 | J | conserved hypothetical protein |
| *so0565* | 0.87 | 1.65 | C | adhesion protein, putative |
| *so0566* | 0.37 | 0.97 | T | ABC 3 transport family protein |
| *so0569* | 1.47 | 1.20 | O | sensory box/GGDEF family protein |
| *so0570* | 0.70 | 0.91 | P | response regulator |
| *so0572* | 1.85 | 1.99 | I | enoyl-CoA hydratase/isomerase family protein |
| *so0576* | 0.38 | -1.31 | T | PhoH family protein |
| *so0585* | -0.08 | -1.26 | T | D-isomer specific 2-hydroxyacid dehydrogenase family protein |
| *so0586* | -0.67 | -1.71 | J | conserved hypothetical protein |
| *so0587* | -0.40 | -0.94 | I | glycerophosphoryl diester phosphodiesterase, putative |
| *so0600* | 0.74 | -0.85 | C | N-acetylmuramoyl-L-alanine amidase |
| *so0608* | -1.13 | 0.09 | H | ubiquinol-cytochrome c reductase, iron-sulfur subunit |
| *so0609* | -1.10 | -0.25 | H | ubiquinol-cytochrome c reductase, cytochrome b |
| *so0611* | -0.95 | -0.51 | D | stringent starvation protein a |
| *so0614* | -0.10 | 2.18 | L | dipeptidyl peptidase IV, putative |
| *so0617* | -0.21 | 1.08 | A | acetylornithine aminotransferase |
| *so0618* | 1.05 | 2.06 | H | arginine N-succinyltransferase |
| *so0619* | 0.27 | 2.10 | H | succinylglutamic semialdehyde dehydrogenase |
| *so0624* | -0.47 | -0.76 | O | catabolite gene activator |
| *so0626* | 0.80 | -0.19 | J | hypothetical protein |
| *so0629* | -0.31 | 1.09 | J | hypothetical protein |
| *so0639* | 2.34 | -0.09 | L | Collagenase family |
| *so0643* | 0.10 | -2.44 | K | transposase, putative |
| *so0668* | 1.38 | -0.43 | K | prophage MuSo1, F protein, putative |
| *so0674* | -0.32 | -0.35 | K | prophage MuSo1, protein Gp32, putative |
| *so0675* | -0.15 | -1.95 | K | prophage MuSo1, major head subunit, putative |
| *so0679* | 0.26 | -0.56 | J | hypothetical protein |
| *so0691* | -0.96 | -0.71 | J | hypothetical protein |
| *so0696* | 0.73 | 0.72 | L | thiol:disulfide interchange protein DsbD |
| *so0698* | -1.05 | -1.09 | T | fxsA protein |
| *so0708* | 0.11 | -1.34 | K | transposase, mutator family |
| *so0709* | -0.74 | -1.20 | J | hypothetical protein |
| *so0711* | -0.85 | -1.57 | J | hypothetical protein |
| *so0715* | 3.16 | 4.43 | T | oxidoreductase, molybdopterin-binding |
| *so0717* | 0.78 | 0.78 | H | monoheme cytochrome c |
| *so0725* | 0.04 | -2.18 | D | catalase/peroxidase HPI |
| *so0730* | -0.77 | -0.85 | J | hypothetical protein |
| *so0740* | -0.36 | -0.66 | D | melanin biosynthesis protein TyrA, putative |
| *so0741* | 0.71 | 0.54 | B | gamma-glutamyltranspeptidase |
| *so0748* | 1.21 | 1.17 | J | hypothetical protein |
| *so0753* | 0.76 | 2.97 | J | hypothetical protein |
| *so0756* | -0.84 | -2.18 | A | phospho-2-dehydro-3-deoxyheptonate aldolase, phe-sensitive |
| *so0762* | 0.97 | -1.56 | T | isochorismatase family protein |
| *so0769* | -1.15 | -0.22 | O | arginine repressor |
| *so0775* | -1.04 | -0.68 | J | conserved hypothetical protein |
| *so0783* | -1.22 | -1.08 | J | hypothetical protein |
| *so0784* | -0.91 | -0.47 | J | hypothetical protein |
| *so0788* | -1.30 | 0.15 | J | conserved hypothetical protein |
| *so0806* | 3.22 | 3.64 | E | alkaline phosphatase, putative |
| *so0807* | -1.26 | -0.67 | N | hypoxanthine-guanine phosphoribosyltransferase |
| *so0808* | -1.47 | -1.33 | J | conserved hypothetical protein |
| *so0813* | -0.75 | -0.07 | J | hypothetical protein |
| *so0814* | 0.30 | -1.24 | J | hypothetical protein |
| *so0815* | -0.78 | 0.37 | T | TonB-dependent receptor C-terminal domain protein |
| *so0824* | -1.05 | -0.40 | J | conserved hypothetical protein |
| *so0826* | 0.39 | 0.70 | J | conserved hypothetical protein |
| *so0837* | 1.49 | 0.85 | D | beta-lactamase, putative |
| *so0842* | -1.21 | -0.39 | M | translation elongation factor G |
| *so0844* | 0.45 | 0.71 | J | hypothetical protein |
| *so0845* | 0.84 | -1.61 | H | cytochrome c-type protein NapB |
| *so0846* | 0.80 | -0.40 | H | iron-sulfur cluster-binding protein napH |
| *so0848* | 0.99 | -1.83 | H | periplasmic nitrate reductase |
| *so0849* | -0.07 | -1.61 | H | napD protein |
| *so0851* | -1.90 | -0.01 | J | hypothetical protein |
| *so0853* | -2.60 | -0.38 | C | pilin, putative |
| *so0854* | -1.20 | -1.00 | C | type IV pilin, putative |
| *so0858* | -0.34 | -0.60 | R | sodium:alanine symporter family protein |
| *so0864* | 2.88 | 2.56 | O | transcriptional regulator, LuxR family |
| *so0865* | 5.94 | 4.02 | J | conserved hypothetical protein |
| *so0866* | 7.00 | 8.08 | D | minor curlin subunit CsgB, putative |
| *so0867* | 5.51 | 2.30 | L | serine protease, subtilase family |
| *so0868* | 0.97 | 1.07 | J | hypothetical protein |
| *so0874* | -1.59 | -0.42 | G | DnaK suppressor protein |
| *so0881* | 0.41 | -0.62 | J | conserved hypothetical protein |
| *so0895* | 1.55 | 0.14 | T | pirin family protein |
| *so0898* | 0.65 | -0.13 | J | conserved hypothetical protein |
| *so0900* | 1.26 | 0.69 | T | oxidoreductase, aldo/keto reductase family |
| *so0902* | -0.76 | -0.06 | H | NADH:ubiquinone oxidoreductase, Na translocating, alpha subunit |
| *so0915* | -0.77 | -0.63 | T | ankyrin domain protein |
| *so0916* | 2.19 | 1.10 | O | transcriptional regulator, MarR family |
| *so0918* | 1.23 | 2.44 | D | aculeacin A acylase |
| *so0919* | -2.96 | -2.52 | R | serine transporter, putative |
| *so0920* | -1.37 | -0.75 | T | acetyltransferase, GNAT family |
| *so0923* | -0.73 | -2.42 | J | conserved hypothetical protein |
| *so0935* | 0.95 | 2.18 | R | Na+/H+ antiporter |
| *so0943* | 0.74 | 1.46 | O | sensory box protein, putative |
| *so0945* | 5.15 | 4.57 | R | AcrB/AcrD/AcrF family protein |
| *so0946* | 5.87 | 7.28 | J | conserved hypothetical protein |
| *so0949* | -0.15 | -1.33 | R | branched-chain amino acid transport system II carrier protein BrnQ |
| *so0958* | 1.37 | 0.05 | D | alkyl hydroperoxide reductase, C subunit |
| *so0962* | -0.90 | -0.92 | J | conserved hypothetical protein |
| *so0970* | -0.50 | -1.71 | H | fumarate reductase flavoprotein subunit precursor |
| *so0994* | 0.85 | 3.81 | J | conserved hypothetical protein |
| *so0996* | -0.99 | -1.27 | T | glyoxalase family protein |
| *so1002* | -0.27 | -1.76 | J | hypothetical protein |
| *so1003* | -1.36 | -2.41 | J | hypothetical protein |
| *so1004* | -1.31 | -2.39 | J | hypothetical protein |
| *so1006* | 1.40 | 0.60 | H | dienelactone hydrolase family protein |
| *so1007* | 2.47 | 0.93 | J | conserved hypothetical protein |
| *so1011* | 0.64 | 1.10 | H | NADH dehydrogenase I, L subunit |
| *so1013* | 0.57 | 1.01 | H | NADH dehydrogenase I, J subunit |
| *so1015* | 0.48 | 1.20 | H | NADH dehydrogenase I, H subunit |
| *so1016* | 0.45 | 1.16 | H | NADH dehydrogenase I, G subunit |
| *so1017* | 0.94 | 1.30 | H | NADH dehydrogenase I, F subunit |
| *so1019* | 1.00 | 1.19 | H | NADH dehydrogenase I, C/D subunits |
| *so1020* | 0.65 | 1.15 | H | NADH dehydrogenase I, B subunit |
| *so1033* | -0.05 | 1.18 | R | iron-compound ABC transporter, ATP-binding protein, putative |
| *so1034* | 0.35 | 1.10 | R | iron-compound ABC transporter, permease protein |
| *so1035* | 0.36 | 1.50 | B | nicotinate-nucleotide--dimethylbenzimidazole phosphoribosyltransferase |
| *so1048* | 0.46 | 1.58 | C | membrane protein, putative |
| *so1049* | 0.61 | 0.92 | T | acetyltransferase, GNAT family |
| *so1059* | 1.16 | 2.29 | L | aminopeptidase N |
| *so1061* | 0.64 | 1.00 | T | TPR domain protein |
| *so1064* | 0.28 | 1.85 | J | conserved hypothetical protein |
| *so1066* | 1.14 | 4.21 | Q | extracellular nuclease |
| *so1067* | 0.13 | 0.94 | J | hypothetical protein |
| *so1072* | -0.94 | 1.61 | R | chitin-binding protein, putative |
| *so1074* | -0.43 | -1.19 | R | tyrosine-specific transport protein, putative |
| *so1075* | 1.16 | 2.96 | J | conserved hypothetical protein |
| *so1087* | -0.98 | -0.69 | J | Na+/H+ antiporter family protein |
| *so1100* | 1.26 | 1.57 | R | extracellular solute-binding protein, family 7 |
| *so1102* | 4.39 | 2.87 | C | TonB-dependent receptor C-terminal region domain lipoprotein |
| *so1109* | -1.14 | -0.28 | B | thiamin biosynthesis lipoprotein ApbE |
| *so1112* | -1.16 | 0.18 | R | bacterioferritin subunit 1 |
| *so1121* | -1.49 | -0.65 | A | glutamate 5-kinase |
| *so1125* | -1.30 | -0.33 | T | integral membrane domain protein |
| *so1143* | 1.41 | 1.11 | J | conserved hypothetical protein |
| *so1144* | -0.82 | -1.48 | D | methyl-accepting chemotaxis protein |
| *so1146* | 1.63 | -3.33 | J | hypothetical protein |
| *so1155* | 0.44 | 0.75 | J | hypothetical protein |
| *so1157* | -1.02 | 0.19 | J | conserved hypothetical protein |
| *so1159* | -1.39 | -0.18 | J | hypothetical protein |
| *so1163* | -0.92 | -0.19 | J | conserved hypothetical protein |
| *so1168* | 0.65 | 2.00 | C | penicillin-binding protein 2 |
| *so1187* | -1.00 | 0.04 | J | hypothetical protein |
| *so1189* | -0.05 | -1.28 | J | conserved hypothetical protein |
| *so1199* | 1.29 | 0.52 | C | phosphoglucosamine mutase |
| *so1202* | -0.96 | 0.46 | J | conserved hypothetical protein |
| *so1212* | -0.26 | -2.39 | J | hypothetical protein |
| *so1215* | -1.64 | -0.10 | C | outer membrane protein OmpK, putative |
| *so1217* | 0.42 | 1.57 | H | deoxyribose-phosphate aldolase |
| *so1218* | -0.63 | 1.27 | N | thymidine phosphorylase |
| *so1219* | -0.10 | 1.30 | N | phosphopentomutase |
| *so1227* | -0.79 | -1.26 | J | conserved hypothetical protein |
| *so1231* | 0.13 | 3.56 | H | TorA specific chaperone |
| *so1232* | 0.07 | 4.09 | H | trimethylamine-N-oxide reductase |
| *so1233* | -0.27 | 4.82 | H | tetraheme cytochrome c |
| *so1234* | -0.80 | -1.30 | H | torE protein |
| *so1236* | -0.54 | -2.68 | R | xanthine/uracil permease family protein |
| *so1237* | -0.09 | -1.31 | H | acetoin utilization protein AcuB, putative |
| *so1245* | 1.03 | 0.90 | C | membrane protein, putative |
| *so1258* | -1.11 | -0.06 | N | adenylosuccinate synthetase, putative |
| *so1264* | 0.01 | -1.79 | J | conserved hypothetical protein |
| *so1265* | -0.41 | -1.81 | O | transcriptional regulator, putative |
| *so1267* | 0.54 | -0.30 | J | conserved hypothetical protein |
| *so1268* | 1.48 | -0.17 | A | glutamine synthetase |
| *so1270* | 0.75 | 0.00 | R | polyamine ABC transporter, periplasmic polyamine-binding protein |
| *so1274* | 0.33 | -2.45 | J | conserved hypothetical protein |
| *so1276* | 0.43 | -1.85 | E | succinate-semialdehyde dehydrogenase |
| *so1278* | 1.60 | 1.27 | D | methyl-accepting chemotaxis protein |
| *so1279* | 1.82 | 1.21 | J | hypothetical protein |
| *so1304* | -1.02 | -0.72 | T | HesB/YadR/YfhF family protein |
| *so1306* | -1.15 | -0.06 | J | conserved hypothetical protein |
| *so1317* | 1.90 | 1.87 | J | hypothetical protein |
| *so1320* | -0.19 | 0.79 | J | hypothetical protein |
| *so1324* | 1.94 | -0.10 | A | glutamate synthase, small subunit |
| *so1325* | 1.58 | -0.38 | A | glutamate synthase, large subunit |
| *so1327* | -1.12 | -0.90 | T | sensor histidine kinase-related protein |
| *so1328* | -0.60 | -1.00 | O | transcriptional regulator, LysR family |
| *so1337* | -0.14 | -1.40 | J | hypothetical protein |
| *so1343* | 2.32 | 1.71 | O | sigma-E factor negative regulatory protein |
| *so1344* | 1.67 | 1.13 | O | sigma-E factor regulatory protein RseB |
| *so1345* | 1.15 | 0.59 | O | sigma-E factor regulatory protein RseC |
| *so1357* | -1.17 | 0.41 | M | ribosomal protein S16 |
| *so1358* | -1.22 | 0.26 | Q | 16S rRNA processing protein RimM |
| *so1359* | -1.03 | -0.03 | M | tRNA (guanine-N1)-methyltransferase |
| *so1360* | -0.99 | -0.12 | M | ribosomal protein L19 |
| *so1363* | 0.12 | -1.30 | H | prismane protein |
| *so1378* | 0.89 | 0.56 | T | ThiJ/PfpI family protein |
| *so1387* | 0.65 | 1.31 | T | HD domain protein |
| *so1390* | -1.23 | -1.27 | L | peptidyl-prolyl cis-trans isomerase, FKBP-type |
| *so1402* | -0.68 | -1.39 | J | conserved hypothetical protein |
| *so1403* | -0.75 | -1.02 | J | conserved hypothetical protein |
| *so1404* | -1.16 | -1.87 | Q | endoribonuclease L-PSP, putative |
| *so1412* | -1.07 | -1.52 | J | conserved hypothetical protein |
| *so1415* | -0.44 | 1.59 | O | transcriptional regulator, TetR family |
| *so1418* | -1.09 | -1.98 | C | apbE family protein |
| *so1422* | -0.57 | -1.31 | O | transcriptional regulator, LysR family |
| *so1423* | -0.63 | -1.46 | J | hypothetical protein |
| *so1424* | -2.19 | -1.50 | J | hypothetical protein |
| *so1425* | -1.50 | -1.15 | J | hypothetical protein |
| *so1427* | -1.77 | -4.37 | H | decaheme cytochrome c |
| *so1428* | 0.65 | -2.88 | C | outer membrane protein |
| *so1429* | 0.28 | -3.29 | H | anaerobic dimethyl sulfoxide reductase, A subunit |
| *so1430* | 0.01 | -4.03 | H | anaerobic dimethyl sulfoxide reductase, B subunit |
| *so1431* | 0.51 | -2.33 | J | conserved hypothetical protein |
| *so1432* | -0.14 | -2.31 | J | hypothetical protein |
| *so1441* | -1.40 | -1.13 | J | hypothetical protein |
| *so1453* | 3.59 | -0.34 | J | conserved hypothetical protein |
| *so1471* | -0.37 | -1.18 | G | site-specific recombinase, phage integrase family |
| *so1473* | -1.22 | -0.25 | M | SsrA-binding protein |
| *so1474* | -1.18 | -0.56 | J | conserved hypothetical protein |
| *so1476* | 0.18 | 1.18 | T | small protein A |
| *so1478* | 3.16 | 5.46 | T | methylase, putative |
| *so1479* | 3.36 | 5.57 | J | hypothetical protein |
| *so1480* | 2.68 | 4.03 | T | GGDEF family protein |
| *so1483* | 3.90 | 0.15 | H | malate synthase A |
| *so1484* | 4.68 | 0.45 | H | isocitrate lyase |
| *so1486* | 1.84 | 2.14 | I | cytosolic long-chain acyl-CoA thioester hydrolase family protein |
| *so1489* | -0.42 | -1.86 | J | hypothetical protein |
| *so1506* | -1.09 | -1.19 | R | MATE efflux family protein |
| *so1507* | -0.86 | -0.53 | J | hypothetical protein |
| *so1519* | 0.77 | 0.83 | H | iron-sulfur cluster-binding protein |
| *so1520* | 0.24 | 0.89 | J | conserved hypothetical protein |
| *so1524* | -1.94 | -0.48 | L | heat shock protein GrpE |
| *so1533* | 0.66 | 0.51 | O | glycine cleavage system transcriptional activator, putative |
| *so1534* | 1.39 | 0.69 | J | conserved hypothetical protein |
| *so1548* | 1.65 | 3.56 | J | hypothetical protein |
| *so1561* | 0.71 | 1.26 | L | peptidase, M1 family |
| *so1567* | -1.37 | -1.00 | J | hypothetical protein |
| *so1568* | -5.14 | -3.93 | J | hypothetical protein |
| *so1573* | 0.41 | 0.74 | J | hypothetical protein |
| *so1578* | -0.64 | -0.80 | O | transcriptional regulator, TetR family |
| *so1587* | 1.16 | -0.14 | J | conserved hypothetical protein |
| *so1589* | 0.66 | 1.38 | J | hypothetical protein |
| *so1605* | -1.11 | 0.33 | C | lipoprotein, putative |
| *so1606* | 1.50 | -2.66 | T | metallo-beta-lactamase superfamily protein |
| *so1608* | -1.85 | -0.48 | J | conserved hypothetical protein |
| *so1623* | 2.00 | 3.26 | P | PTS system, glucose-specific IIBC component |
| *so1629* | -1.15 | -0.39 | M | ribosomal protein S2 |
| *so1630* | -1.43 | -0.47 | M | translation elongation factor Ts |
| *so1631* | -1.08 | -0.54 | N | uridylate kinase |
| *so1632* | -1.24 | -0.68 | M | ribosome recycling factor |
| *so1637* | 0.48 | 0.92 | C | bacterial surface antigen |
| *so1640* | 0.33 | 0.97 | I | (3R)-hydroxymyristoyl-(acyl-carrier-protein) dehydratase |
| *so1641* | 0.84 | 1.04 | C | acyl-(acyl-carrier-protein)--UDP-N-acetylglucosamine O-acyltransferase |
| *so1642* | 0.94 | 1.11 | C | lipid A disaccharide synthase |
| *so1646* | 1.06 | 0.50 | T | GGDEF family protein |
| *so1649* | 2.77 | 2.47 | J | conserved hypothetical protein |
| *so1650* | 1.97 | 1.45 | J | conserved hypothetical protein |
| *so1659* | 0.83 | 1.54 | H | decaheme cytochrome c |
| *so1661* | 2.47 | 3.11 | O | transcriptional regulator, LysR family |
| *so1666* | 3.33 | 3.24 | H | phenylalanine-4-hydroxylase |
| *so1667* | 2.51 | 0.27 | B | pterin-4-alpha-carbinolamine dehydratase |
| *so1671* | 0.89 | 1.18 | E | glutathione S-transferase family protein |
| *so1672* | 0.85 | 0.81 | J | conserved hypothetical protein |
| *so1673* | 0.64 | 3.69 | C | outer membrane protein OmpW, putative |
| *so1677* | 0.98 | -0.36 | I | acetyl-CoA acetyltransferase |
| *so1678* | 1.25 | 0.11 | H | methylmalonate-semialdehyde dehydrogenase |
| *so1679* | 1.30 | 0.49 | I | acyl-CoA dehydrogenase family protein |
| *so1680* | 0.95 | 0.79 | I | enoyl-CoA hydratase/isomerase family protein |
| *so1698* | 1.63 | 2.38 | J | hypothetical protein |
| *so1699* | 2.98 | 2.75 | O | transcriptional regulator |
| *so1700* | 4.95 | 4.11 | J | hypothetical protein |
| *so1701* | 4.75 | 2.55 | J | hypothetical protein |
| *so1702* | 0.57 | 1.19 | J | hypothetical protein |
| *so1705* | 1.77 | 0.41 | J | conserved hypothetical protein |
| *so1708* | 0.18 | 1.67 | H | 4-hydroxybutyrate coenzyme A transferase |
| *so1723* | 0.41 | 1.36 | R | phosphate ABC transporter, permease protein, putative |
| *so1724* | 0.76 | 1.42 | R | phosphate ABC transporter, permease protein, putative |
| *so1736* | 0.41 | -0.81 | J | conserved hypothetical protein |
| *so1748* | -0.03 | -1.61 | J | hypothetical protein |
| *so1750* | 1.43 | 0.90 | R | ABC transporter, ATP-binding protein |
| *so1751* | 1.28 | 0.72 | C | membrane protein, putative |
| *so1753* | 1.02 | 0.96 | J | hypothetical protein |
| *so1760* | 3.01 | 1.56 | R | AzlC family protein |
| *so1770* | -1.90 | -0.22 | J | glycerate kinase, putative |
| *so1771* | -1.63 | 0.65 | R | permease, GntP family |
| *so1778* | -0.35 | -1.08 | H | decaheme cytochrome c |
| *so1779* | -0.43 | -1.17 | H | decaheme cytochrome c |
| *so1786* | -1.07 | -0.31 | M | glutaminyl-tRNA synthetase |
| *so1787* | 3.83 | 2.31 | J | conserved hypothetical protein |
| *so1790* | -1.03 | -0.40 | L | peptidyl-prolyl cis-trans isomerase B |
| *so1793* | -1.46 | -0.64 | L | trigger factor |
| *so1797* | -1.11 | -1.20 | G | DNA-binding protein, HU family |
| *so1806* | 2.63 | 2.42 | O | psp operon transcriptional activator |
| *so1807* | -1.49 | -0.85 | D | phage shock protein A |
| *so1808* | -0.86 | -0.75 | D | phage shock protein B |
| *so1809* | -1.12 | -1.00 | D | phage shock protein C |
| *so1812* | 3.10 | 3.46 | H | methionine gamma-lyase |
| *so1813* | 3.04 | 1.71 | T | DNA-binding protein, putative |
| *so1821* | -2.29 | -3.22 | R | outer membrane porin, putative |
| *so1822* | 3.35 | 4.99 | R | TonB-dependent receptor, putative |
| *so1824* | -2.01 | -0.19 | J | conserved hypothetical protein |
| *so1825* | -1.42 | 0.00 | R | MotA/TolQ/ExbB proton channel family protein |
| *so1826* | -0.92 | -0.07 | R | TonB system transport protein ExbB2 |
| *so1829* | -1.47 | -0.56 | T | TPR domain protein |
| *so1831* | -0.29 | 1.27 | J | conserved hypothetical protein |
| *so1836* | -1.63 | -0.72 | J | conserved hypothetical protein |
| *so1844* | 2.63 | 0.91 | Q | extracellular nuclease, putative |
| *so1856* | -0.91 | -0.76 | I | 3-hydroxydecanoyl-(acyl-carrier-protein) dehydratase |
| *so1867* | 0.30 | -1.02 | J | conserved hypothetical protein |
| *so1873* | -1.35 | -2.20 | J | conserved hypothetical protein |
| *so1881* | 1.13 | 3.39 | T | HlyD family-related protein |
| *so1882* | 0.84 | 2.24 | R | AcrB/AcrD/AcrF family protein |
| *so1883* | -0.98 | -1.04 | T | acetyltransferase, GNAT family |
| *so1901* | 2.30 | -0.04 | L | sugE protein |
| *so1909* | 1.99 | -0.34 | J | conserved hypothetical protein |
| *so1915* | 4.79 | 3.59 | L | serine protease, subtilase family |
| *so1921* | -1.14 | -0.34 | J | hypothetical protein |
| *so1923* | 2.24 | 1.97 | R | AcrB/AcrD/AcrF family protein |
| *so1924* | 2.79 | 3.06 | R | AcrB/AcrD/AcrF family protein |
| *so1925* | 1.59 | 1.27 | R | HlyD family secretion protein |
| *so1928* | -0.41 | 0.80 | H | succinate dehydrogenase, flavoprotein subunit |
| *so1932* | -1.04 | 0.69 | H | succinyl-CoA synthase, beta subunit |
| *so1935* | -0.64 | -0.60 | O | regulator of nucleoside diphosphate kinase |
| *so1944* | 1.13 | 1.80 | J | hypothetical protein |
| *so1945* | 0.60 | 1.35 | P | sensor protein PhoQ |
| *so1946* | 0.09 | 1.78 | P | transcriptional regulatory protein PhoP |
| *so1948* | -1.23 | -0.02 | R | sodium:dicarboxylate symporter family protein |
| *so1949* | 0.38 | 2.48 | T | invasin domain protein |
| *so1954* | -1.17 | -0.63 | R | transporter, LysE family |
| *so1955* | -0.65 | 1.43 | J | hypothetical protein |
| *so1961* | 2.96 | -1.57 | D | maltose O-acetyltransferase |
| *so1962* | 2.70 | 0.82 | H | 4-hydroxyphenylpyruvate dioxygenase |
| *so1963* | 3.12 | 1.06 | J | conserved hypothetical protein |
| *so1964* | 1.40 | 1.17 | J | hypothetical protein |
| *so1967* | -1.49 | -2.58 | J | hypothetical protein |
| *so1970* | 0.85 | 3.29 | J | hypothetical protein |
| *so1971* | 1.91 | 2.57 | T | AMP-binding family protein |
| *so2001* | -1.08 | 0.35 | N | 5-nucleotidase |
| *so2002* | 0.18 | 2.04 | J | hypothetical protein |
| *so2012* | -0.30 | 1.06 | N | adenine phosphoribosyltransferase |
| *so2018* | -1.21 | -0.56 | N | adenylate kinase |
| *so2027* | -1.01 | -0.92 | J | hypothetical protein |
| *so2041* | -0.76 | -0.63 | J | conserved hypothetical protein |
| *so2042* | 1.61 | 1.91 | J | conserved hypothetical protein |
| *so2043* | 1.04 | 0.82 | J | conserved hypothetical protein |
| *so2046* | 0.77 | 1.72 | O | transcriptional regulator, MarR family |
| *so2047* | 1.82 | 2.29 | L | prolyl oligopeptidase family protein |
| *so2049* | 1.66 | 1.73 | T | GGDEF family protein |
| *so2059* | 1.23 | -0.10 | J | hypothetical protein |
| *so2061* | 0.64 | 0.68 | J | hypothetical protein |
| *so2062* | -0.81 | -0.88 | J | conserved hypothetical protein |
| *so2063* | -1.00 | -0.86 | J | hypothetical protein |
| *so2069* | 1.36 | 1.97 | A | phosphoribosylformimino-5-aminoimidazole carboxamide ribotide isomerase |
| *so2070* | 2.72 | 2.07 | A | amidotransferase HisH |
| *so2071* | 2.82 | 2.26 | A | imidazoleglycerol-phosphate dehydratase/histidinol-phosphatase |
| *so2074* | 1.41 | -0.54 | A | ATP phosphoribosyltransferase |
| *so2083* | 0.88 | 2.64 | D | methyl-accepting chemotaxis protein |
| *so2089* | 1.31 | 2.14 | L | hydrogenase expression/formation protein HypA |
| *so2090* | 2.87 | 2.17 | L | hydrogenase expression/formation protein HypE |
| *so2092* | 1.74 | 1.79 | L | hydrogenase assembly chaperone hypC/hupF |
| *so2093* | 1.24 | 2.21 | L | hydrogenase accessory protein HypB |
| *so2097* | 1.66 | 2.05 | H | quinone-reactive Ni/Fe hydrogenase, cytochrome b subunit |
| *so2098* | 3.51 | 2.24 | H | quinone-reactive Ni/Fe hydrogenase, large subunit |
| *so2099* | 4.09 | 2.40 | H | quinone-reactive Ni/Fe hydrogenase, small subunit precursor |
| *so2100* | 1.18 | 0.63 | H | thioredoxin family protein |
| *so2101* | 0.70 | 0.57 | C | lipoprotein, putative |
| *so2102* | 0.08 | -1.66 | J | hypothetical protein |
| *so2114* | -0.31 | -0.96 | J | conserved hypothetical protein |
| *so2116* | 1.46 | 0.75 | T | acetyltransferase, GNAT family |
| *so2117* | 0.66 | 1.59 | D | methyl-accepting chemotaxis protein |
| *so2121* | 1.25 | 1.55 | D | chemotaxis protein CheA |
| *so2122* | 1.36 | 2.22 | D | purine-binding chemotaxis protein CheW |
| *so2123* | 1.32 | 1.40 | D | methyl-accepting chemotaxis protein |
| *so2174* | 1.61 | 1.80 | G | GGDEF domain protein |
| *so2178* | 0.84 | 0.43 | D | cytochrome c551 peroxidase |
| *so2191* | 1.20 | 0.44 | A | cystathionine beta-lyase |
| *so2199* | 1.48 | 3.72 | J | hypothetical protein |
| *so2216* | 1.52 | 2.25 | O | sensory box protein |
| *so2221* | 0.93 | 0.19 | B | para-aminobenzoate synthase, component I |
| *so2223* | 0.22 | 1.57 | L | peptidase, putative |
| *so2224* | 0.30 | 1.78 | T | acetyltransferase, GNAT family |
| *so2244* | 0.84 | 1.35 | O | transcriptional regulator, LacI family |
| *so2260* | -0.77 | 2.18 | T | extragenic suppressor protein SuhB |
| *so2263* | -1.13 | -0.81 | T | Rrf2 family protein |
| *so2266* | -0.74 | -0.68 | T | HesB/YadR/YfhF family protein |
| *so2269* | -1.47 | -0.58 | H | ferredoxin, 2Fe-2S |
| *so2273* | 1.06 | 2.38 | J | hypothetical protein |
| *so2274* | -1.28 | -0.82 | N | nucleoside diphosphate kinase |
| *so2290* | -0.98 | 0.32 | T | rhodanese domain protein |
| *so2291* | -0.99 | -0.72 | J | hypothetical protein |
| *so2303* | -0.91 | -0.96 | H | thioredoxin reductase |
| *so2305* | -1.40 | -0.02 | O | leucine-responsive regulatory protein |
| *so2328* | -1.18 | -0.40 | M | translation elongation factor P |
| *so2341* | 0.69 | 0.56 | H | alpha keto acid dehydrogenase complex, E2 component |
| *so2347* | -0.94 | -1.77 | H | glyceraldehyde 3-phosphate dehydrogenase |
| *so2348* | -0.91 | -1.31 | J | hypothetical protein |
| *so2352* | 0.52 | 1.26 | T | bax protein, putative |
| *so2353* | 0.45 | 1.24 | J | hypothetical protein |
| *so2360* | -0.99 | 1.02 | J | conserved hypothetical protein |
| *so2361* | -0.68 | 2.26 | H | cytochrome c oxidase, cbb3-type, subunit III |
| *so2363* | -0.72 | 2.20 | H | cytochrome c oxidase, cbb3-type, subunit II |
| *so2364* | -0.58 | 2.29 | H | cytochrome c oxidase, cbb3-type, subunit I |
| *so2384* | -1.18 | -1.26 | G | site-specific recombinase, phage integrase family |
| *so2387* | -0.98 | -0.07 | J | conserved hypothetical protein |
| *so2389* | -4.44 | -4.55 | R | multidrug resistance protein D |
| *so2402* | -1.03 | -0.20 | M | ribosomal protein S1 |
| *so2407* | 1.36 | -3.25 | J | conserved hypothetical protein |
| *so2408* | 1.30 | -1.71 | L | radical activating enzyme |
| *so2419* | 0.71 | -0.95 | I | 2,4-dienoyl-CoA reductase, putative |
| *so2420* | 0.78 | 1.24 | L | signal peptide peptidase SppA, 67K type |
| *so2427* | -3.88 | -1.23 | R | TonB-dependent receptor, putative |
| *so2434* | 1.35 | 1.64 | R | extracellular solute-binding proteins, family 3/GGDEF domain protein |
| *so2439* | 1.01 | 0.53 | T | srpA-related protein |
| *so2445* | 1.26 | -0.36 | B | thiamin biosynthesis protein ThiC |
| *so2446* | 3.21 | 3.44 | J | hypothetical protein |
| *so2451* | -1.02 | -0.32 | J | conserved hypothetical protein |
| *so2455* | -0.48 | -1.29 | O | transcriptional regulator, LysR family |
| *so2460* | 5.73 | 6.83 | J | hypothetical protein |
| *so2469* | -0.30 | -1.61 | T | conserved hypothetical protein |
| *so2471* | 1.00 | 0.72 | A | succinyl-diaminopimelate desuccinylase |
| *so2480* | -0.53 | -0.57 | J | hypothetical protein |
| *so2481* | -0.99 | 0.55 | J | conserved hypothetical protein |
| *so2483* | -2.10 | -3.82 | A | aspartate aminotransferase, putative |
| *so2487* | 1.05 | 0.04 | H | 6-phosphogluconate dehydratase |
| *so2493* | 0.35 | -1.81 | O | transcriptional regulator, TetR family |
| *so2494* | 0.19 | 1.81 | L | zinc-dependent metallopeptidase |
| *so2507* | 1.22 | 1.40 | T | GGDEF domain protein |
| *so2535* | -0.93 | -0.79 | J | conserved hypothetical protein |
| *so2559* | 1.64 | 1.28 | G | DNA polymerase III, epsilon subunit |
| *so2566* | 1.30 | 1.68 | T | asmA protein |
| *so2569* | -1.09 | -1.86 | J | hypothetical protein |
| *so2570* | 1.48 | 3.04 | C | lipoprotein, putative |
| *so2572* | -0.72 | -0.27 | L | peptidyl-prolyl cis-trans isomerase B |
| *so2585* | 0.62 | 1.09 | J | hypothetical protein |
| *so2587* | 1.78 | 1.54 | B | delta-aminolevulinic acid dehydratase |
| *so2589* | -0.93 | -0.88 | T | oxidoreductase, iron/ascorbate family |
| *so2593* | -1.17 | -1.13 | J | conserved hypothetical protein |
| *so2594* | 3.16 | 2.89 | J | conserved hypothetical protein |
| *so2595* | 2.69 | 2.55 | T | BNR repeat protein |
| *so2596* | 4.34 | 4.21 | J | conserved hypothetical protein |
| *so2597* | 4.73 | 4.57 | J | conserved hypothetical protein |
| *so2603* | -1.09 | -0.49 | J | conserved hypothetical protein |
| *so2631* | 0.88 | -0.48 | G | MutT/nudix family protein |
| *so2637* | -1.09 | -1.34 | J | hypothetical protein |
| *so2643* | 0.98 | 1.10 | T | oxidoreductase, FAD-binding, putative |
| *so2648* | 0.98 | 0.61 | P | DNA-binding response regulator, LuxR family |
| *so2654* | 1.40 | -0.53 | K | transposase, putative |
| *so2658* | 0.44 | 0.43 | J | hypothetical protein |
| *so2669* | -1.25 | -1.26 | J | hypothetical protein |
| *so2670* | -1.27 | -1.46 | J | hypothetical protein |
| *so2671* | 0.13 | 1.30 | J | conserved hypothetical protein |
| *so2680* | 0.31 | 1.11 | J | conserved hypothetical protein |
| *so2684* | -0.63 | 1.03 | K | prophage MuSo2, protein Gp32, putative |
| *so2685* | -0.61 | 1.22 | K | prophage MuSo2, major head subunit, putative |
| *so2686* | 1.15 | 1.81 | J | hypothetical protein |
| *so2687* | -0.93 | 1.03 | J | hypothetical protein |
| *so2689* | 0.62 | 1.43 | J | conserved hypothetical protein |
| *so2690* | -0.41 | 0.49 | K | prophage MuSo2, virion morphogenesis protein, putative |
| *so2702* | 0.28 | 1.23 | J | conserved hypothetical protein |
| *so2706* | 0.95 | 1.95 | H | succinylarginine dihydrolase |
| *so2708* | 0.25 | 1.22 | T | nitroreductase family protein |
| *so2710* | 0.98 | 1.45 | J | conserved hypothetical protein |
| *so2711* | 2.17 | 2.63 | J | conserved hypothetical protein |
| *so2727* | -0.99 | -1.48 | H | cytochrome c3 |
| *so2736* | -1.24 | -2.11 | J | conserved hypothetical protein |
| *so2737* | -1.37 | -0.89 | B | dethiobiotin synthase |
| *so2738* | -0.81 | -0.78 | B | biotin synthesis protein BioC |
| *so2743* | 1.45 | 0.12 | H | acetyl-coenzyme A synthetase |
| *so2763* | -0.98 | -0.40 | J | conserved hypothetical protein |
| *so2768* | 1.17 | 0.59 | I | acyl-CoA dehydrogenase family protein |
| *so2769* | 1.00 | -0.12 | J | conserved hypothetical protein |
| *so2781* | -0.95 | -0.51 | J | conserved hypothetical protein |
| *so2791* | 0.06 | 0.81 | N | cytidine deaminase |
| *so2797* | -1.49 | -0.35 | J | conserved hypothetical protein |
| *so2820* | -0.22 | -1.92 | F | ISSo11, transposase |
| *so2821* | -0.22 | -2.48 | J | conserved hypothetical protein |
| *so2826* | 0.89 | -4.92 | J | hypothetical protein |
| *so2830* | -0.69 | -0.65 | J | hypothetical protein |
| *so2846* | -0.78 | 1.09 | J | conserved hypothetical protein |
| *so2848* | 0.57 | 0.67 | J | hypothetical protein |
| *so2854* | 0.65 | -0.19 | J | hypothetical protein |
| *so2855* | 1.54 | 0.23 | T | exonuclease |
| *so2856* | 1.61 | 1.06 | T | CBS domain protein |
| *so2857* | 3.50 | 0.34 | R | sodium/solute symporter family protein |
| *so2865* | -2.46 | -1.84 | R | L-lysine exporter, putative |
| *so2869* | 0.85 | 1.01 | J | conserved hypothetical protein |
| *so2876* | -0.71 | -1.43 | J | hypothetical protein |
| *so2879* | 0.29 | 1.30 | R | uracil permease |
| *so2882* | 2.30 | 2.45 | J | conserved hypothetical protein |
| *so2887* | 1.36 | 1.95 | L | disulfide bond formation protein b |
| *so2888* | 1.07 | 1.18 | J | hypothetical protein |
| *so2890* | 0.05 | 0.92 | J | hypothetical protein |
| *so2900* | 1.96 | 2.57 | J | hypothetical protein |
| *so2907* | -2.41 | 0.53 | T | TonB-dependent receptor domain protein |
| *so2911* | -0.43 | 1.36 | R | formate transporter, putative |
| *so2912* | 0.09 | 1.23 | H | formate acetyltransferase |
| *so2913* | 0.12 | 1.44 | H | pyruvate formate-lyase 1 activating enzyme |
| *so2914* | -0.28 | 0.89 | J | conserved hypothetical protein |
| *so2915* | 0.12 | 1.12 | H | acetate kinase |
| *so2916* | 0.64 | 1.46 | H | phosphate acetyltransferase |
| *so2923* | -0.89 | -3.03 | R | sodium/glutamate symporter |
| *so2924* | 3.00 | 2.34 | L | signal peptidase I family protein |
| *so2926* | 2.46 | 2.04 | R | ABC transporter, permease, putative |
| *so2927* | 2.38 | 1.98 | R | ABC transporter, ATP-binding protein |
| *so2928* | 0.86 | 0.92 | I | acyl-CoA thioesterase I, putative |
| *so2929* | 1.66 | 1.61 | J | hypothetical protein |
| *so2930* | 2.73 | 3.89 | J | hypothetical protein |
| *so2931* | 1.67 | 1.85 | J | hypothetical protein |
| *so2933* | -0.93 | -0.27 | L | sohB protein, peptidase U7 family |
| *so2934* | 3.39 | 5.28 | J | conserved hypothetical protein |
| *so2938* | -0.08 | -0.66 | J | hypothetical protein |
| *so2940* | 1.81 | 0.37 | K | prophage LambdaSo, host specificity protein J, putative |
| *so2942* | -1.00 | -0.79 | J | hypothetical protein |
| *so2945* | 1.04 | 0.12 | J | hypothetical protein |
| *so2947* | -0.20 | 2.48 | J | hypothetical protein |
| *so2962* | -0.05 | 0.10 | J | hypothetical protein |
| *so2972* | -0.62 | -0.27 | J | hypothetical protein |
| *so2973* | -0.23 | 0.13 | K | prophage LambdaSo, lysozyme, putative |
| *so2995* | 0.10 | -0.49 | J | hypothetical protein |
| *so2998* | 0.50 | -0.78 | J | hypothetical protein |
| *so3000* | 0.66 | -1.32 | J | conserved hypothetical protein |
| *so3001* | 0.61 | -1.32 | J | hypothetical protein |
| *so3002* | 0.34 | -1.19 | J | conserved hypothetical protein |
| *so3003* | 0.33 | 0.07 | J | hypothetical protein |
| *so3004* | 0.19 | -1.42 | G | prophage LambdaSo, DNA modification methyltransferase, putative |
| *so3027* | 1.24 | 1.68 | J | hypothetical protein |
| *so3048* | 1.52 | 0.30 | H | isoquinoline 1-oxidoreductase, beta subunit, putative |
| *so3056* | -0.60 | -0.58 | H | tetraheme cytochrome c |
| *so3062* | 2.13 | 1.83 | J | hypothetical protein |
| *so3063* | 2.93 | 3.07 | R | sodium:alanine symporter family protein |
| *so3080* | 3.24 | 1.85 | T | hemK family protein |
| *so3084* | 0.00 | 1.78 | O | sensory box protein |
| *so3085* | 0.38 | 3.28 | J | conserved domain protein |
| *so3090* | 2.25 | 1.57 | T | MoxR domain protein |
| *so3091* | 1.72 | 1.35 | J | conserved hypothetical protein |
| *so3092* | 1.91 | 1.75 | J | hypothetical protein |
| *so3093* | 1.91 | 1.67 | T | von Willebrand factor type A domain protein |
| *so3094* | 1.72 | 1.15 | T | TPR domain protein |
| *so3095* | 0.95 | 1.00 | J | conserved hypothetical protein |
| *so3096* | 0.73 | 1.01 | Q | RNA polymerase sigma-70 factor, ECF subfamily |
| *so3097* | 1.04 | 1.02 | J | conserved hypothetical protein |
| *so3099* | -5.82 | -4.70 | R | long-chain fatty acid transport protein, putative |
| *so3102* | 1.10 | 0.65 | T | AcrA/AcrE family protein |
| *so3106* | 5.45 | 5.94 | L | cold-active serine alkaline protease |
| *so3109* | 1.02 | 1.19 | J | conserved hypothetical protein |
| *so3110* | 0.85 | 1.29 | L | protein-export membrane protein SecF |
| *so3111* | 0.20 | 0.98 | L | protein-export membrane protein SecD |
| *so3113* | -0.52 | 1.21 | M | queuine tRNA-ribosyltransferase |
| *so3114* | 0.63 | 0.65 | M | S-adenosylmethionine:tRNA ribosyltransferase-isomerase |
| *so3115* | 0.56 | -0.13 | J | hypothetical protein |
| *so3119* | -1.81 | -2.18 | J | hypothetical protein |
| *so3120* | 1.49 | 2.10 | T | oxidoreductase, Gfo/Idh/MocA family |
| *so3122* | -1.70 | -1.51 | R | sodium/dicarboxylate symporter |
| *so3133* | 2.04 | 0.71 | J | hypothetical protein |
| *so3142* | 0.52 | 1.33 | L | peptidyl-dipeptidase Dcp |
| *so3146* | -1.18 | -0.30 | G | DNA-binding protein, H-NS family |
| *so3150* | 2.10 | 0.45 | C | lipoprotein, putative |
| *so3162* | 0.67 | 1.05 | O | sensor histidine kinase |
| *so3166* | 0.77 | 0.76 | J | conserved hypothetical protein |
| *so3172* | -2.22 | -1.22 | C | galactosyl transferase |
| *so3184* | -1.10 | -0.48 | J | conserved hypothetical protein |
| *so3185* | -0.97 | -0.18 | C | polysaccharide biosynthesis protein |
| *so3246* | -1.94 | 1.06 | J | hypothetical protein |
| *so3257* | 0.31 | 0.67 | J | conserved hypothetical protein |
| *so3275* | -2.32 | -1.20 | J | hypothetical protein |
| *so3278* | 2.39 | 2.87 | J | conserved hypothetical protein |
| *so3282* | 0.77 | 1.28 | D | methyl-accepting chemotaxis protein |
| *so3290* | -1.31 | -1.32 | J | hypothetical protein |
| *so3291* | -0.90 | -1.56 | T | cytidine/deoxycytidylate deaminase family protein |
| *so3292* | -0.04 | -1.23 | N | GMP synthase |
| *so3293* | -0.46 | -1.24 | N | inosine-5-monophosphate dehydrogenase |
| *so3297* | -1.19 | -0.57 | O | transcriptional regulator, LysR family |
| *so3298* | -1.14 | -1.88 | J | conserved hypothetical protein |
| *so3300* | -1.16 | -0.78 | H | cytochrome c |
| *so3301* | -1.38 | -0.66 | T | flavocytochrome c flavin subunit |
| *so3305* | -1.93 | -1.56 | P | DNA-binding response regulator, LuxR family |
| *so3308* | 0.68 | 0.95 | T | GTP-binding protein EngA |
| *so3312* | 0.17 | 0.66 | T | 1-hydroxy-2-methyl-2-(E)-butenyl 4-diphosphate synthase |
| *so3319* | 0.86 | 0.23 | J | conserved hypothetical protein |
| *so3325* | 0.04 | -1.47 | T | nrfJ-related protein |
| *so3331* | 1.46 | 2.87 | J | conserved hypothetical protein |
| *so3334* | 0.99 | 0.93 | T | GGDEF family protein |
| *so3342* | -1.17 | -0.88 | J | conserved hypothetical protein |
| *so3350* | 1.18 | 1.27 | D | twitching motility protein PilU |
| *so3361* | 0.51 | 1.49 | J | conserved hypothetical protein |
| *so3370* | 1.94 | -0.30 | J | conserved hypothetical protein |
| *so3371* | 1.39 | 0.67 | J | conserved hypothetical protein |
| *so3390* | 1.78 | 0.38 | J | hypothetical protein |
| *so3392* | 1.24 | -1.60 | T | oxidoreductase, FMN-binding |
| *so3393* | 0.00 | -1.54 | O | transcriptional regulator, TetR family |
| *so3395* | 2.15 | 4.06 | J | hypothetical protein |
| *so3404* | 1.15 | 0.95 | D | methyl-accepting chemotaxis protein |
| *so3409* | -0.54 | -0.78 | T | OsmC/Ohr family protein |
| *so3410* | 0.72 | 1.29 | J | hypothetical protein |
| *so3411* | 0.91 | 1.84 | L | protease, putative |
| *so3422* | 0.33 | -1.33 | M | ribosomal subunit interface protein |
| *so3423* | -1.08 | -0.22 | G | DNA polymerase III, chi subunit |
| *so3432* | 3.16 | 2.38 | Q | RNA polymerase sigma factor RpoS |
| *so3433* | 1.44 | 2.59 | C | lipoprotein NlpD |
| *so3479* | -0.18 | 1.99 | J | hypothetical protein |
| *so3480* | 1.90 | 2.92 | J | conserved hypothetical protein |
| *so3483* | 0.48 | 0.50 | R | HlyD family secretion protein |
| *so3489* | 2.42 | 2.56 | T | GGDEF domain protein |
| *so3497* | 1.87 | 1.46 | T | aminotransferase, class III |
| *so3502* | 0.90 | 0.53 | J | hypothetical protein |
| *so3503* | -0.50 | 0.97 | R | glucose/galactose transporter |
| *so3507* | -0.57 | 0.70 | J | conserved hypothetical protein |
| *so3514* | -0.31 | 2.88 | J | conserved hypothetical protein |
| *so3516* | 1.10 | 2.42 | O | transcriptional regulator, LacI family |
| *so3524* | 0.65 | 1.39 | C | type IV pilus biogenesis protein PilE |
| *so3525* | 0.94 | 1.06 | C | type IV pilin biogenesis protein, putative |
| *so3527* | 0.92 | 1.50 | J | hypothetical protein |
| *so3528* | 0.71 | 1.66 | J | hypothetical protein |
| *so3540* | -1.22 | -0.59 | J | conserved hypothetical protein |
| *so3542* | -0.03 | 0.79 | J | conserved hypothetical protein |
| *so3558* | 0.90 | 0.48 | J | hypothetical protein |
| *so3560* | 1.66 | 1.99 | L | peptidase, M16 family |
| *so3565* | -1.87 | 2.10 | N | 2,3-cyclic-nucleotide 2-phosphodiesterase |
| *so3580* | 0.58 | 0.87 | J | conserved hypothetical protein |
| *so3585* | 1.57 | -1.22 | D | azoreductase, putative |
| *so3586* | 1.75 | -0.72 | T | glyoxalase family protein |
| *so3599* | -0.87 | -1.82 | R | sulfate ABC transporter, periplasmic sulfate-binding protein |
| *so3615* | 0.93 | 0.78 | J | hypothetical protein |
| *so3626* | -0.82 | -0.63 | J | hypothetical protein |
| *so3637* | 0.97 | 0.61 | L | survival protein surA |
| *so3638* | 1.18 | 0.92 | B | pyridoxal phosphate biosynthetic protein PdxA |
| *so3639* | 1.05 | 0.89 | M | dimethyladenosine transferase |
| *so3651* | -0.92 | -0.27 | M | ribosomal protein L27 |
| *so3656* | 1.19 | 0.02 | J | hypothetical protein |
| *so3657* | 0.37 | 0.08 | R | transporter, LysE family |
| *so3659* | 2.83 | 3.53 | L | thiol:disulfide interchange protein, putative |
| *so3664* | -0.09 | -1.15 | I | long-chain-fatty-acid--CoA ligase |
| *so3667* | -1.53 | -0.06 | J | conserved hypothetical protein |
| *so3668* | -0.90 | -0.22 | J | conserved hypothetical protein |
| *so3669* | -1.40 | -0.36 | R | heme transport protein |
| *so3670* | -0.88 | -0.22 | R | TonB1 protein |
| *so3671* | -0.95 | 0.41 | R | TonB system transport protein ExbB1 |
| *so3673* | -0.75 | 0.12 | R | hemin ABC transporter, periplasmic hemin-binding protein |
| *so3676* | -1.02 | -0.95 | J | hypothetical protein |
| *so3678* | 0.86 | 0.26 | J | conserved hypothetical protein |
| *so3681* | 1.06 | 0.18 | D | universal stress protein family |
| *so3682* | 1.05 | 0.09 | J | hypothetical protein |
| *so3685* | 4.77 | 1.59 | D | curli production assembly/transport component CsgG, putative |
| *so3686* | 2.94 | 1.09 | D | curli production assembly/transport component CsgF, putative |
| *so3687* | 6.80 | 4.82 | D | curli production assembly/transport component CsgE, putative |
| *so3688* | 1.25 | 1.36 | P | nitrogen regulation protein NtrY, putative |
| *so3689* | 1.80 | 0.50 | P | sigma-54 dependent nitrogen response regulator |
| *so3692* | 1.26 | 0.52 | R | ABC transporter, ATP-binding protein |
| *so3705* | -3.27 | -5.18 | E | 5-methylthioadenosine nucleosidase/S-adenosylhomocysteine nucleosidase, putative |
| *so3706* | -2.46 | -3.20 | R | NupC family protein |
| *so3708* | -0.13 | -1.86 | C | membrane protein, putative |
| *so3715* | -1.11 | -0.63 | T | oxygen-insensitive NAD(P)H nitroreductase |
| *so3718* | -1.09 | -1.00 | L | thiol:disulfide interchange protein, DsbA family |
| *so3719* | -0.13 | -1.22 | J | hypothetical protein |
| *so3720* | -0.45 | -1.14 | J | conserved hypothetical protein |
| *so3740* | 0.26 | 0.93 | H | NAD(P) transhydrogenase, alpha subunit |
| *so3741* | 0.90 | 0.72 | H | NAD(P) transhydrogenase, beta subunit |
| *so3748* | -1.23 | -0.47 | T | LysM domain protein |
| *so3749* | -0.98 | -0.37 | J | hypothetical protein |
| *so3766* | 0.76 | -0.04 | J | hypothetical protein |
| *so3770* | -1.04 | 0.64 | J | conserved hypothetical protein TIGR00153 |
| *so3771* | -1.02 | 0.16 | R | phosphate transporter, putative |
| *so3774* | -0.07 | 1.39 | H | proline dehydrogenase/delta-1-pyrroline-5-carboxylate dehydrogenase, putative |
| *so3782* | 0.83 | 1.91 | J | hypothetical protein |
| *so3783* | -0.98 | -0.52 | Q | ATP-dependent RNA helicase, DEAD box family |
| *so3784* | -1.38 | 0.65 | J | hypothetical protein |
| *so3787* | -0.30 | -0.80 | J | hypothetical protein |
| *so3791* | -0.40 | -1.29 | L | renal dipeptidase family protein |
| *so3800* | 3.63 | 3.13 | L | serine protease, subtilase family |
| *so3803* | -1.05 | -0.96 | N | hypoxanthine phosphoribosyltransferase |
| *so3807* | 0.68 | 1.86 | T | sterol desaturase family protein |
| *so3808* | 0.64 | 2.65 | J | conserved hypothetical protein |
| *so3810* | 0.01 | 1.12 | T | OmpA-like transmembrane domain protein |
| *so3815* | -1.00 | -0.60 | J | conserved hypothetical protein |
| *so3830* | -1.09 | -0.35 | J | conserved hypothetical protein |
| *so3840* | -0.74 | -1.23 | Q | RNA polymerase sigma-70 factor, ECF subfamily |
| *so3841* | -0.66 | -1.10 | J | hypothetical protein |
| *so3842* | -1.03 | -0.50 | J | conserved hypothetical protein |
| *so3844* | 0.72 | 2.12 | L | peptidase, M13 family |
| *so3846* | 0.64 | 2.02 | J | conserved hypothetical protein |
| *so3848* | -1.20 | -0.63 | J | hypothetical protein |
| *so3849* | 0.56 | 1.21 | J | conserved domain protein |
| *so3852* | -1.25 | 0.58 | J | conserved hypothetical protein |
| *so3856* | -1.21 | -1.00 | J | hypothetical protein |
| *so3857* | -0.94 | -0.49 | T | PAP2 family protein |
| *so3859* | -0.51 | -0.95 | G | cyclic nucleotide phosphodiesterase, putative |
| *so3863* | 0.53 | 2.49 | R | molybdenum ABC transporter, periplasmic molybdenum-binding protein |
| *so3865* | -0.27 | 1.44 | R | molybdenum ABC transporter, ATP-binding protein |
| *so3874* | -1.08 | -0.07 | O | transcriptional regulator, LysR family |
| *so3883* | -0.49 | -1.19 | J | hypothetical protein |
| *so3890* | -0.74 | 0.24 | D | methyl-accepting chemotaxis protein |
| *so3896* | -0.80 | -1.61 | R | outer membrane porin, putative |
| *so3905* | -1.22 | -1.08 | J | conserved hypothetical protein |
| *so3907* | -0.95 | -0.42 | J | conserved hypothetical protein |
| *so3908* | -0.59 | -1.39 | I | enoyl-CoA hydratase/isomerase family protein |
| *so3909* | -0.36 | -1.49 | J | conserved hypothetical protein |
| *so3913* | 0.13 | 1.13 | J | conserved hypothetical protein |
| *so3923* | 0.00 | 1.26 | B | thiH protein, putative |
| *so3924* | 0.41 | 1.37 | J | hypothetical protein |
| *so3925* | 1.18 | 1.36 | T | biotin synthase family protein |
| *so3926* | 1.43 | 1.43 | T | GTP-binding protein |
| *so3927* | -1.04 | 0.11 | M | ribosomal protein L9 |
| *so3928* | -1.44 | -0.21 | M | ribosomal protein S18 |
| *so3930* | -0.83 | -0.42 | M | ribosomal protein S6 |
| *so3933* | 0.30 | 0.72 | C | membrane protein, putative |
| *so3937* | -1.03 | -0.33 | N | adenylosuccinate synthetase |
| *so3938* | -1.24 | -0.64 | J | conserved hypothetical protein |
| *so3939* | -1.41 | -0.67 | M | ribosomal protein S9 |
| *so3940* | -0.90 | -0.47 | M | ribosomal protein L13 |
| *so3942* | 2.08 | 1.34 | L | serine protease, HtrA/DegQ/DegS family |
| *so3951* | -0.22 | -0.13 | J | conserved hypothetical protein |
| *so3969* | -4.14 | -3.25 | C | OmpA family protein |
| *so3970* | -0.71 | -1.71 | J | hypothetical protein |
| *so3972* | -1.45 | -1.41 | J | conserved hypothetical protein |
| *so3980* | -0.09 | -2.90 | H | cytochrome c552 nitrite reductase |
| *so3981* | -0.20 | 0.56 | P | nitrate/nitrite sensor protein NarQ |
| *so3982* | -0.34 | -0.44 | P | DNA-binding nitrate/nitrite response regulator |
| *so3983* | -1.41 | -0.35 | J | conserved hypothetical protein |
| *so3985* | 0.10 | -0.09 | J | conserved hypothetical protein |
| *so3988* | -2.29 | -2.20 | P | aerobic respiration control protein ArcA |
| *so3993* | -0.71 | -0.79 | J | hypothetical protein |
| *so3994* | -0.96 | -0.76 | J | hypothetical protein |
| *so3997* | -0.81 | -1.01 | J | conserved hypothetical protein TIGR00645 |
| *so4012* | -0.75 | -1.06 | J | hypothetical protein |
| *so4013* | -1.19 | -1.13 | J | hypothetical protein |
| *so4014* | -4.01 | -3.57 | R | AcrB/AcrD/AcrF family protein |
| *so4015* | -2.94 | -3.60 | J | conserved hypothetical protein |
| *so4018* | 2.06 | 0.75 | J | hypothetical protein |
| *so4036* | -2.02 | -1.56 | J | hypothetical protein |
| *so4045* | 1.23 | -0.32 | J | hypothetical protein |
| *so4052* | 0.32 | -0.44 | O | transcriptional regulator, MarR family |
| *so4068* | -0.54 | -0.50 | J | hypothetical protein |
| *so4074* | 2.37 | 1.66 | J | hypothetical protein |
| *so4075* | 0.74 | 0.14 | J | hypothetical protein |
| *so4077* | 1.61 | 0.38 | R | TonB-dependent receptor, putative |
| *so4081* | -1.06 | -0.19 | R | amino acid permease |
| *so4104* | -0.68 | -1.02 | D | MSHA pilin protein MshC |
| *so4105* | -0.29 | -1.63 | D | MSHA pilin protein MshA |
| *so4106* | -0.20 | -1.36 | D | MSHA pilin protein MshB |
| *so4126* | 1.36 | -0.73 | J | hypothetical protein |
| *so4131* | -0.46 | -2.00 | J | conserved hypothetical protein |
| *so4133* | -1.04 | -1.05 | N | uridine phosphorylase |
| *so4134* | 0.48 | -1.41 | J | conserved hypothetical protein |
| *so4138* | -1.62 | -0.85 | J | conserved hypothetical protein |
| *so4139* | -1.45 | -0.69 | J | conserved domain protein |
| *so4144* | 2.01 | 0.94 | H | cytochrome c, putative |
| *so4145* | 3.05 | 2.59 | J | conserved hypothetical protein |
| *so4146* | 2.74 | 2.91 | L | toxin secretion ABC transporter protein, HlyB family |
| *so4147* | 2.94 | 2.90 | R | ABC transporter, ATP-binding/permease protein |
| *so4148* | 4.81 | 4.01 | R | HlyD family secretion protein |
| *so4149* | 3.79 | 3.30 | D | RTX toxin, putative |
| *so4159* | -2.59 | -1.48 | J | hypothetical protein |
| *so4189* | -0.99 | -0.12 | J | conserved hypothetical protein |
| *so4209* | 1.36 | 0.92 | J | conserved hypothetical protein |
| *so4229* | 1.52 | 0.37 | J | hypothetical protein |
| *so4233* | -1.73 | -1.07 | A | 3-isopropylmalate dehydratase, small subunit |
| *so4237* | 0.94 | 0.28 | J | hypothetical protein |
| *so4242* | -1.50 | -0.72 | J | conserved hypothetical protein |
| *so4246* | -1.62 | -0.81 | M | ribosomal protein L33 |
| *so4247* | -1.58 | -0.43 | M | ribosomal protein L28 |
| *so4248* | -1.63 | -0.43 | G | DNA repair protein RadC |
| *so4252* | 2.10 | 2.55 | L | prolyl oligopeptidase family protein |
| *so4255* | -0.85 | -1.38 | N | orotate phosphoribosyltransferase |
| *so4281* | -0.75 | 1.61 | R | potassium uptake protein KtrA, putative |
| *so4282* | -0.15 | 1.18 | R | potassium uptake protein KtrB |
| *so4283* | -0.11 | 1.59 | C | apbE family protein |
| *so4290* | 0.72 | 1.19 | R | phosphate ABC transporter, permease protein |
| *so4292* | 0.31 | 1.96 | R | phosphate ABC transporter, periplasmic phosphate-binding protein |
| *so4295* | 1.05 | 1.54 | H | NAD(P)H dehydrogenase (quinone) |
| *so4317* | 1.43 | 1.21 | D | RTX toxin, putative |
| *so4318* | 0.85 | 0.95 | L | toxin secretion ATP-binding protein |
| *so4320* | 1.17 | 1.64 | D | agglutination protein |
| *so4321* | 0.95 | 0.95 | C | OmpA family protein |
| *so4322* | 1.03 | 1.15 | J | conserved hypothetical protein |
| *so4329* | -0.59 | -0.75 | J | conserved hypothetical protein |
| *so4335* | -0.67 | -1.24 | I | phosphatidylglycerophosphatase B, putative |
| *so4340* | -2.15 | -1.73 | J | conserved hypothetical protein |
| *so4343* | -0.15 | -0.83 | T | aminotransferase, class V |
| *so4344* | 0.56 | 1.07 | A | threonine dehydratase |
| *so4349* | 0.89 | 1.39 | A | ketol-acid reductoisomerase |
| *so4355* | -1.14 | -1.32 | J | hypothetical protein |
| *so4356* | -0.41 | -1.03 | J | conserved domain protein |
| *so4366* | 0.69 | 1.18 | J | conserved hypothetical protein |
| *so4367* | 0.26 | 0.99 | T | acyltransferase family protein |
| *so4371* | 0.89 | 1.20 | J | conserved hypothetical protein |
| *so4374* | 0.84 | 1.24 | H | histidine ammonia-lyase, putative |
| *so4375* | 0.35 | 1.18 | J | conserved hypothetical protein |
| *so4378* | 0.87 | 1.31 | T | FAD-binding protein |
| *so4380* | 0.88 | 0.84 | I | 3-oxoacyl-(acyl-carrier-protein) synthase II, putative |
| *so4381* | 0.57 | 0.77 | T | thioester dehydrase family protein |
| *so4382* | 0.62 | 1.05 | I | 3-oxoacyl-(acyl-carrier-protein) reductase |
| *so4383* | 0.74 | 0.97 | I | 3-oxoacyl-(acyl-carrier-protein) synthase II |
| *so4384* | 0.14 | 1.28 | J | hypothetical protein |
| *so4387* | -1.44 | -0.97 | P | sensor histidine kinase |
| *so4394* | 0.78 | -0.63 | D | phage shock protein E |
| *so4396* | 1.27 | -0.04 | I | acyl carrier protein phosphodiesterase |
| *so4403* | 0.69 | 0.09 | J | hypothetical protein |
| *so4404* | 1.35 | 1.51 | H | iron-sulfur cluster-binding protein |
| *so4407* | 0.03 | -1.16 | T | GGDEF family protein |
| *so4408* | -1.01 | -0.16 | D | virulence regulator BipA |
| *so4410* | -0.01 | -1.20 | A | glutamine synthetase, type I |
| *so4413* | 2.11 | 2.20 | J | conserved hypothetical protein |
| *so4414* | 2.06 | 1.15 | J | conserved domain protein |
| *so4416* | -2.49 | -1.25 | J | hypothetical protein |
| *so4420* | -1.31 | -0.54 | L | peptidase, M23/M37 family |
| *so4435* | -1.36 | -1.91 | J | hypothetical protein |
| *so4444* | -1.24 | -0.89 | P | capsular synthesis regulator component B, putative |
| *so4454* | 1.19 | 1.51 | D | methyl-accepting chemotaxis protein |
| *so4456* | 0.83 | 1.04 | J | conserved hypothetical protein |
| *so4457* | 1.69 | 2.66 | T | GGDEF domain protein |
| *so4463* | 1.71 | 2.07 | T | prolyl 4-hydroxylase, alpha subunit domain protein |
| *so4467* | -0.72 | -0.99 | J | conserved hypothetical protein |
| *so4469* | -0.96 | -1.34 | H | alcohol dehydrogenase, iron-containing |
| *so4473* | -1.13 | 1.75 | C | outer membrane protein, putative |
| *so4480* | 0.87 | -0.47 | H | aldehyde dehydrogenase |
| *so4483* | 0.99 | 0.64 | H | cytochrome b, putative |
| *so4507* | 0.15 | -0.78 | H | TorA specific chaperone, putative |
| *so4509* | 1.30 | 0.60 | H | formate dehydrogenase, alpha subunit |
| *so4512* | -1.66 | -1.47 | J | conserved hypothetical protein |
| *so4513* | -0.22 | -1.06 | H | formate dehydrogenase, alpha subunit |
| *so4515* | 0.08 | -0.64 | H | formate dehydrogenase, C subunit, putative |
| *so4520* | -0.50 | -1.42 | B | oxygen-independent coproporphyrinogen III oxidase, putative |
| *so4524* | 1.06 | 1.50 | O | transcriptional regulator, LysR family |
| *so4527* | 0.56 | 1.20 | T | integral membrane domain protein |
| *so4542* | 1.72 | 3.51 | O | transcriptional regulator, LysR family |
| *so4543* | 1.02 | -0.18 | J | hypothetical protein |
| *so4557* | -1.54 | -0.26 | D | methyl-accepting chemotaxis protein |
| *so4558* | -0.52 | -1.52 | J | hypothetical protein |
| *so4562* | 2.33 | 1.95 | J | conserved hypothetical protein |
| *so4564* | 1.20 | 0.28 | R | TonB2 protein, putative |
| *so4565* | 0.54 | 1.22 | R | transporter, putative |
| *so4571* | -1.83 | -1.78 | O | transcriptional regulator, LysR family |
| *so4572* | -0.89 | -0.91 | H | cytochrome c, putative |
| *so4574* | 0.49 | -0.01 | T | hydrolase, alpha/beta fold family |
| *so4583* | 0.81 | 0.22 | Q | RNA polymerase sigma-32 factor |
| *so4591* | -0.40 | -1.65 | H | tetraheme cytochrome c |
| *so4592* | 2.45 | 1.40 | J | hypothetical protein |
| *so4593* | 2.00 | 1.61 | J | hypothetical protein |
| *so4594* | -0.22 | -0.86 | J | hypothetical protein |
| *so4596* | -0.76 | -1.01 | T | copper-transporting ATPase domain protein |
| *so4597* | -0.12 | -1.22 | R | heavy metal efflux system protein, putative |
| *so4598* | 0.40 | -1.13 | R | heavy metal efflux pump, CzcA family |
| *so4606* | 2.34 | -0.38 | H | cytochrome c oxidase, subunit II |
| *so4609* | 1.92 | 0.59 | H | cytochrome c oxidase subunit III |
| *so4612* | 1.23 | -0.14 | J | conserved hypothetical protein |
| *so4618* | 0.86 | 1.43 | L | prolyl oligopeptidase family protein |
| *so4619* | -0.89 | -1.03 | T | yhgI protein |
| *so4624* | -0.05 | 0.65 | O | transcriptional regulator, LuxR family |
| *so4625* | 1.58 | 1.17 | D | competence protein ComF |
| *so4627* | 1.44 | 0.60 | J | hypothetical protein |
| *so4628* | 2.26 | 3.02 | T | sulfatase |
| *so4634* | 0.65 | 1.36 | P | osmolarity sensor protein EnvZ |
| *so4635* | 0.94 | 2.90 | D | methyl-accepting chemotaxis protein |
| *so4645* | 1.40 | 2.31 | J | hypothetical protein |
| *so4649* | 1.12 | 0.83 | J | conserved hypothetical protein |
| *so4654* | -1.42 | -1.67 | R | sulfate ABC transporter, permease protein |
| *so4666* | -1.65 | -0.38 | H | cytochrome c |
| *so4673* | -1.33 | -1.29 | H | threonine 3-dehydrogenase |
| *so4674* | -1.43 | -1.29 | H | 2-amino-3-ketobutyrate coenzyme A ligase |
| *so4675* | 0.46 | 0.46 | O | transcriptional regulator, TetR family |
| *so4681* | -2.84 | -2.23 | C | glycosyl transferase, group 1 family protein |
| *so4688* | 0.04 | -1.07 | C | glycosyl transferase, group 2 family protein |
| *so4694* | -0.70 | 2.68 | J | hypothetical protein |
| *so4700* | -0.01 | -2.09 | J | hypothetical protein |
| *so4705* | 1.13 | 0.35 | O | transcriptional regulator, putative |
| *so4712* | 0.02 | 1.31 | R | ABC transporter, ATP-binding protein, putative |
| *so4731* | -0.30 | 1.06 | N | adenosine deaminase |
| *so4742* | 1.13 | -0.25 | O | transcriptional regulator, DeoR family |
| *so4743* | -1.21 | -0.76 | R | TonB-dependent receptor, putative |
| *so4745* | -0.96 | -0.20 | C | UDP-N-acetylglucosamine pyrophosphorylase |
| *so4747* | -0.99 | -0.06 | H | ATP synthase F1, beta subunit |
| *so4748* | -0.96 | 0.20 | H | ATP synthase F1, gamma subunit |
| *so4749* | -1.57 | -0.21 | H | ATP synthase F1, alpha subunit |
| *so4750* | -1.45 | -0.20 | H | ATP synthase F1, delta subunit |
| *so4751* | -1.61 | -0.01 | H | ATP synthase F0, B subunit |
| *so4752* | -1.16 | 0.06 | H | ATP synthase F0, C subunit |
| *so4753* | -1.08 | 0.05 | H | ATP synthase F0, A subunit |
| *soa0003* | -1.08 | -0.78 | G | type II restriction endonuclease, putative |
| *soa0004* | -1.04 | -0.46 | G | type II DNA modification methyltransferase |
| *soa0036* | 0.68 | 0.88 | T | HicB-related protein |
| *soa0051* | -1.07 | -1.54 | J | hypothetical protein |
| *soa0086* | -0.69 | 2.18 | G | site-specific recombinase, resolvase family |
| *soa0096* | 0.67 | 1.01 | K | partitioning protein B |
| *soa0109* | 1.13 | 1.64 | J | hypothetical protein |
| *soa0132* | -0.26 | 0.96 | J | conserved hypothetical protein |
| *soa0138* | -1.32 | -1.86 | J | hypothetical protein |
| *soa0139* | -1.27 | -1.09 | J | hypothetical protein |
| *soa0140* | -1.24 | -1.48 | J | hypothetical protein |
| *soa0141* | -1.14 | -1.47 | J | hypothetical protein |
| ^a^ FC, functional category. | | | | |
