# Supplementary material for: Probing regulon of ArcA in Shewanella oneidensis MR-1 by integrated genomic analyses
Source: BMC Genomics. 2008 Jan 25;9:42. doi: 10.1186/1471-2164-9-42 (PMC2262068; doi:10.1186/1471-2164-9-42)
Supplement: Additional file 3 — Genes that exhibit significant changes in the ΔarcA strain (for discussion). The data provided represent all genes listed in additional file 2 that were discussed in details. [file 1471-2164-9-42-S3.doc]

| **TABLE S2. Genes that exhibit significant changes in the *∆arcA* strain (for discussion)** | | | | | | |
| --- | --- | --- | --- | --- | --- | --- |
| Gene | Ratio (log2)  +O_2_ -O_2_ | | FC^a^ | BS^b^ | C^c^ | Function |
| *so0017* | 1.94 | 3.06 | J |  | I | conserved hypothetical protein |
| *so0090* | 1.52 | 3.42 | J |  | I | hypothetical protein |
| *so0185* | 1.42 | 1.53 | J |  | I | conserved hypothetical protein |
| *so0266* |  |  |  |  |  |  |
| *so0306* | 3.46 | 2.54 | J |  | I | hypothetical protein |
| *so0445* | 1.42 | 2.05 | L |  | I | hflC protein, putative |
| *so0553* | 1.68 | 3.42 | J |  | I | hypothetical protein |
| *so0618* | 1.05 | 2.06 | H |  | I | arginine N-succinyltransferase |
| *so0715* | 3.16 | 4.43 | T |  | I | oxidoreductase, molybdopterin-binding |
| *so0806* | 3.22 | 3.64 | E |  | I | alkaline phosphatase, putative |
| *so0864* | 2.88 | 2.56 | O |  | I | transcriptional regulator, LuxR family |
| *so0865* | 5.94 | 4.02 | J |  | I | conserved hypothetical protein |
| *so0866* | 7.00 | 8.08 | D | -136 | I | minor curlin subunit CsgB, putative |
| *so0867* | 5.51 | 2.30 | L | -328 | I | serine protease, subtilase family |
| *so0916* | 2.19 | 1.10 | O | -150 | I | transcriptional regulator, MarR family |
| *so0918* | 1.23 | 2.44 | D |  | I | aculeacin A acylase |
| *so0945* | 5.15 | 4.57 | R |  | I | AcrB/AcrD/AcrF family protein |
| *so0946* | 5.87 | 7.28 | J |  | I | conserved hypothetical protein |
| *so1019* | 1.00 | 1.19 | H |  | I | NADH dehydrogenase I, C/D subunits |
| *so1066* | 1.14 | 4.21 | Q |  | I | extracellular nuclease |
| *so1075* | 1.16 | 2.96 | J |  | I | conserved hypothetical protein |
| *so1102* | 4.39 | 2.87 | C |  | I | TonB-dependent receptor C-terminal region domain lipoprotein |
| *so1343* | 2.32 | 1.71 | O |  | I | sigma-E factor negative regulatory protein |
| *so1478* | 3.16 | 5.46 | T |  | I | methylase, putative |
| *so1479* | 3.36 | 5.57 | J |  | I | hypothetical protein |
| *so1480* | 2.68 | 4.03 | T |  | I | GGDEF family protein |
| *so1548* | 1.65 | 3.56 | J |  | I | hypothetical protein |
| *so1623* | 2.00 | 3.26 | P | -472 | I | PTS system, glucose-specific IIBC component |
| *so1649* | 2.77 | 2.47 | J |  | I | conserved hypothetical protein |
| *so1650* | 1.97 | 1.45 | J |  | I | conserved hypothetical protein |
| *so1661* | 2.47 | 3.11 | O | -219 | I | transcriptional regulator, LysR family |
| *so1666* | 3.33 | 3.24 | H |  | I | phenylalanine-4-hydroxylase |
| *so1698* | 1.63 | 2.38 | J |  | I | hypothetical protein |
| *so1699* | 2.98 | 2.75 | O |  | I | transcriptional regulator |
| *so1700* | 4.95 | 4.11 | J |  | I | hypothetical protein |
| *so1701* | 4.75 | 2.55 | J |  | I | hypothetical protein |
| *so1760* | 3.01 | 1.56 | R |  | I | AzlC family protein |
| *so1787* | 3.83 | 2.31 | J |  | I | conserved hypothetical protein |
| *so1806* | 2.63 | 2.42 | O | -90 | I | psp operon transcriptional activator |
| *so1812* | 3.10 | 3.46 | H | -84 | I | methionine gamma-lyase |
| *so1813* | 3.04 | 1.71 | T |  | I | DNA-binding protein, putative |
| *so1822* | 3.35 | 4.99 | R | -133 | I | TonB-dependent receptor, putative |
| *so1881* | 1.13 | 3.39 | T |  | I | HlyD family-related protein |
| *so1915* | 4.79 | 3.59 | L |  | I | serine protease, subtilase family |
| *so1923* | 2.24 | 1.97 | R |  | I | AcrB/AcrD/AcrF family protein |
| *so1924* | 2.79 | 3.06 | R |  | I | AcrB/AcrD/AcrF family protein |
| *so1925* | 1.59 | 1.27 | R |  | I | HlyD family secretion protein |
| *so1963* | 3.12 | 1.06 | J |  | I | conserved hypothetical protein |
| *so1971* | 1.91 | 2.57 | T |  | I | AMP-binding family protein |
| *so2069* | 1.36 | 1.97 | A |  | I | phosphoribosylformimino-5-aminoimidazole carboxamide ribotide isomerase |
| *so2070* | 2.72 | 2.07 | A |  | I | amidotransferase HisH |
| *so2071* | 2.82 | 2.26 | A |  | I | imidazoleglycerol-phosphate dehydratase/histidinol-phosphatase |
| *so2089* | 1.31 | 2.14 | L |  | I | hydrogenase expression/formation protein HypA |
| *so2090* | 2.87 | 2.17 | L |  | I | hydrogenase expression/formation protein HypE |
| *so2092* | 1.74 | 1.79 | L |  | I | hydrogenase assembly chaperone hypC/hupF |
| *so2093* | 1.24 | 2.21 | L |  | I | hydrogenase accessory protein HypB |
| *so2097* | 1.66 | 2.05 | H |  | I | quinone-reactive Ni/Fe hydrogenase, cytochrome b subunit |
| *so2098* | 3.51 | 2.24 | H |  | I | quinone-reactive Ni/Fe hydrogenase, large subunit |
| *so2099* | 4.09 | 2.40 | H | -147 | I | quinone-reactive Ni/Fe hydrogenase, small subunit precursor |
| *so2199* | 1.48 | 3.72 | J | -101 | I | hypothetical protein |
| *so2273* | 1.06 | 2.38 | J |  | I | hypothetical protein |
| *so2446* | 3.21 | 3.44 | J |  | I | hypothetical protein |
| *so2460* | 5.73 | 6.83 | J |  | I | hypothetical protein |
| *so2570* | 1.48 | 3.04 | C |  | I | lipoprotein, putative |
| *so2594* | 3.16 | 2.89 | J |  | I | conserved hypothetical protein |
| *so2595* | 2.69 | 2.55 | T |  | I | BNR repeat protein |
| *so2596* | 4.34 | 4.21 | J |  | I | conserved hypothetical protein |
| *so2597* | 4.73 | 4.57 | J |  | I | conserved hypothetical protein |
| *so2711* | 2.17 | 2.63 | J |  | I | conserved hypothetical protein |
| *so2856* | 1.61 | 1.06 | T |  | I | CBS domain protein |
| *so2882* | 2.30 | 2.45 | J |  | I | conserved hypothetical protein |
| *so2900* | 1.96 | 2.57 | J |  | I | hypothetical protein |
| *so2924* | 3.00 | 2.34 | L |  | I | signal peptidase I family protein |
| *so2926* | 2.46 | 2.04 | R |  | I | ABC transporter, permease, putative |
| *so2927* | 2.38 | 1.98 | R |  | I | ABC transporter, ATP-binding protein |
| *so2929* | 1.66 | 1.61 | J |  | I | hypothetical protein |
| *so2930* | 2.73 | 3.89 | J |  | I | hypothetical protein |
| *so2931* | 1.67 | 1.85 | J |  | I | hypothetical protein |
| *so2934* | 3.39 | 5.28 | J |  | I | conserved hypothetical protein |
| *so3063* | 2.93 | 3.07 | R |  | I | sodium:alanine symporter family protein |
| *so3080* | 3.24 | 1.85 | T |  | I | hemK family protein |
| *so3090* | 2.25 | 1.57 | T |  | I | MoxR domain protein |
| *so3091* | 1.72 | 1.35 | J |  | I | conserved hypothetical protein |
| *so3092* | 1.91 | 1.75 | J |  | I | hypothetical protein |
| *so3093* | 1.91 | 1.67 | T |  | I | von Willebrand factor type A domain protein |
| *so3094* | 1.72 | 1.15 | T |  | I | TPR domain protein |
| *so3106* | 5.45 | 5.94 | L | -127 | I | cold-active serine alkaline protease |
| *so3278* | 2.39 | 2.87 | J | 41 | I | conserved hypothetical protein |
| *so3331* | 1.46 | 2.87 | J |  | I | conserved hypothetical protein |
| *so3350* | 1.18 | 1.27 | D |  | I | twitching motility protein PilU |
| *so3395* | 2.15 | 4.06 | J | -14 | I | hypothetical protein |
| *so3432* | 3.16 | 2.38 | Q |  | I | RNA polymerase sigma factor RpoS |
| *so3433* | 1.44 | 2.59 | C |  | I | lipoprotein NlpD |
| *so3480* | 1.90 | 2.92 | J | -310 | I | conserved hypothetical protein |
| *so3489* | 2.42 | 2.56 | T | -41 | I | GGDEF domain protein |
| *so3516* | 1.10 | 2.42 | O |  | I | transcriptional regulator, LacI family |
| *so3659* | 2.83 | 3.53 | L |  | I | thiol:disulfide interchange protein, putative |
| *so3685* | 4.77 | 1.59 | D |  | I | curli production assembly/transport component CsgG, putative |
| *so3686* | 2.94 | 1.09 | D |  | I | curli production assembly/transport component CsgF, putative |
| *so3687* | 6.80 | 4.82 | D |  | I | curli production assembly/transport component CsgE, putative |
| *so3800* | 3.63 | 3.13 | L |  | I | serine protease, subtilase family |
| *so4074* | 2.37 | 1.66 | J |  | I | hypothetical protein |
| *so4145* | 3.05 | 2.59 | J |  | I | conserved hypothetical protein |
| *so4146* | 2.74 | 2.91 | L |  | I | toxin secretion ABC transporter protein, HlyB family |
| *so4147* | 2.94 | 2.90 | R |  | I | ABC transporter, ATP-binding/permease protein |
| *so4148* | 4.81 | 4.01 | R |  | I | HlyD family secretion protein |
| *so4149* | 3.79 | 3.30 | D |  | I | RTX toxin, putative |
| *so4252* | 2.10 | 2.55 | L |  | I | prolyl oligopeptidase family protein |
| *so4320* | 1.17 | 1.64 | D |  | I | agglutination protein |
| *so4322* | 1.03 | 1.15 | J |  | I | conserved hypothetical protein |
| *so4413* | 2.11 | 2.20 | J |  | I | conserved hypothetical protein |
| *so4414* | 2.06 | 1.15 | J |  | I | conserved domain protein |
| *so4457* | 1.69 | 2.66 | T | -63 | I | GGDEF domain protein |
| *so4542* | 1.72 | 3.51 | O |  | I | transcriptional regulator, LysR family |
| *so4562* | 2.33 | 1.95 | J |  | I | conserved hypothetical protein |
| *so4592* | 2.45 | 1.40 | J | -312 | I | hypothetical protein |
| *so4593* | 2.00 | 1.61 | J |  | I | hypothetical protein |
| *so4628* | 2.26 | 3.02 | T |  | I | sulfatase |
| *so4645* | 1.40 | 2.31 | J |  | I | hypothetical protein |
| *so0184* | 2.12 | -0.46 | J |  | II | conserved hypothetical protein |
| *so0186* | 2.57 | 0.37 | J |  | II | conserved hypothetical protein |
| *so0187* | 1.40 | 0.15 | L |  | II | serine protease, subtilase family |
| *so0188* | 1.10 | -0.22 | J |  | II | hypothetical protein |
| *so0189* | 2.33 | 0.27 | T |  | II | fibronectin type III domain protein |
| *so0342* | 1.90 | 0.20 | J |  | II | conserved hypothetical protein |
| *so0343* | 2.35 | 0.08 | H | -53 | II | aconitate hydratase 1 |
| *so0344* | 2.01 | 0.42 | H |  | II | methylcitrate synthase |
| *so0345* | 2.02 | 0.51 | H |  | II | methylisocitrate lyase |
| *so0346* | 1.10 | 0.21 | O |  | II | transcriptional regulator. GntR family |
| *so0639* | 2.34 | -0.09 | L |  | II | Collagenase family |
| *so1007* | 2.47 | 0.93 | J |  | II | conserved hypothetical protein |
| *so1324* | 1.94 | -0.10 | A |  | II | glutamate synthase, small subunit |
| *so1325* | 1.58 | -0.38 | A |  | II | glutamate synthase, large subunit |
| *so1453* | 3.59 | -0.34 | J |  | II | conserved hypothetical protein |
| *so1483* | 3.90 | 0.15 | H | -291 | II | malate synthase A |
| *so1484* | 4.68 | 0.45 | H |  | II | isocitrate lyase |
| *so1667* | 2.51 | 0.27 | B |  | II | pterin-4-alpha-carbinolamine dehydratase |
| *so1844* | 2.63 | 0.91 | Q |  | II | extracellular nuclease, putative |
| *so1962* | 2.70 | 0.82 | H |  | II | 4-hydroxyphenylpyruvate dioxygenase |
| *so2074* | 1.41 | -0.54 | A |  | II | ATP phosphoribosyltransferase |
| *so2855* | 1.54 | 0.23 | T |  | II | exonuclease |
| *so2857* | 3.50 | 0.34 | R |  | II | sodium/solute symporter family protein |
| *so3370* | 1.94 | -0.30 | J |  | II | conserved hypothetical protein |
| *so3371* | 1.39 | 0.67 | J |  | II | conserved hypothetical protein |
| *so3586* | 1.75 | -0.72 | T |  | II | glyoxalase family protein |
| *so3638* | 1.18 | 0.92 | B |  | II | pyridoxal phosphate biosynthetic protein PdxA |
| *so3639* | 1.05 | 0.89 | M |  | II | dimethyladenosine transferase |
| *so4606* | 2.34 | -0.38 | H |  | II | cytochrome c oxidase, subunit II |
| *so1146* | 1.14 | -3.33 | J |  | III | hypothetical protein |
| *so1606* | 1.50 | -2.66 | T |  | III | metallo-beta-lactamase superfamily protein |
| *so1961* | 2.96 | -1.57 | D | -114 | III | maltose O-acetyltransferase |
| *so2407* | 1.36 | -3.25 | J |  | III | conserved hypothetical protein |
| *so2408* | 1.30 | -1.71 | L |  | III | radical activating enzyme |
| *so3585* | 1.57 | -1.22 | D |  | III | azoreductase, putative |
| *so0232* | -0.10 | 1.18 | M |  | IV | ribosomal protein L4 |
| *so0617* | -0.21 | 1.08 | A |  | IV | acetylornithine aminotransferase |
| *so0619* | 0.27 | 2.10 | H |  | IV | succinylglutamic semialdehyde dehydrogenase |
| *so0753* | 0.76 | 2.97 | J |  | IV | hypothetical protein |
| *so0994* | 0.85 | 3.81 | J |  | IV | conserved hypothetical protein |
| *so1011* | 0.64 | 1.10 | H |  | IV | NADH dehydrogenase I, L subunit |
| *so1013* | 0.57 | 1.01 | H |  | IV | NADH dehydrogenase I, J subunit |
| *so1015* | 0.48 | 1.20 | H |  | IV | NADH dehydrogenase I, H subunit |
| *so1016* | 0.45 | 1.16 | H |  | IV | NADH dehydrogenase I, G subunit |
| *so1017* | 0.94 | 1.30 | H |  | IV | NADH dehydrogenase I, F subunit |
| *so1020* | 0.65 | 1.15 | H |  | IV | NADH dehydrogenase I, B subunit |
| *so1021* | -0.09 | 1.11 | H |  | IV | NADH dehydrogenase I, A subunit |
| *so1033* | -0.05 | 1.18 | R |  | IV | iron-compound ABC transporter, ATP-binding protein, putative |
| *so1034* | 0.35 | 1.10 | R |  | IV | iron-compound ABC transporter, permease protein |
| *so1035* | 0.36 | 1.50 | B | -83 | IV | nicotinate-nucleotide--dimethylbenzimidazole phosphoribosyltransferase |
| *so1217* | 0.42 | 1.57 | H |  | IV | deoxyribose-phosphate aldolase |
| *so1218* | -0.63 | 1.27 | N |  | IV | thymidine phosphorylase |
| *so1219* | -0.10 | 1.30 | N |  | IV | phosphopentomutase |
| *so1231* | 0.13 | 3.56 | H |  | IV | TorA specific chaperone |
| *so1232* | 0.07 | 4.09 | H |  | IV | trimethylamine-N-oxide reductase |
| *so1233* | -0.27 | 4.82 | H |  | IV | tetraheme cytochrome c |
| *so1641* | 0.84 | 1.04 | C |  | IV | acyl-(acyl-carrier-protein)--UDP-N-acetylglucosamine O-acyltransferase |
| *so1642* | 0.94 | 1.11 | C |  | IV | lipid A disaccharide synthase |
| *so1659* | 0.83 | 1.54 | H | -260 | IV | decaheme cytochrome c |
| *so1673* | 0.64 | 3.69 | C | 288 | IV | outer membrane protein OmpW, putative |
| *so1882* | 0.84 | 2.24 | R |  | IV | AcrB/AcrD/AcrF family protein |
| *so1945* | 0.60 | 1.35 | P |  | IV | sensor protein PhoQ |
| *so1946* | 0.09 | 1.78 | P |  | IV | transcriptional regulatory protein PhoP |
| *so1949* | 0.38 | 2.48 | T |  | IV | invasin domain protein |
| *so1970* | 0.85 | 3.29 | J |  | IV | hypothetical protein |
| *so2083* | 0.88 | 2.64 | D |  | IV | methyl-accepting chemotaxis protein |
| *so2360* | -0.99 | 1.02 | J |  | IV | conserved hypothetical protein |
| *so2361* | -0.68 | 2.26 | H |  | IV | cytochrome c oxidase, cbb3-type, subunit III |
| *so2362* | -0.68 | 2.26 | H |  | IV | cytochrome c oxidase, cbb3-type, CcoQ subunit |
| *so2363* | -0.72 | 2.20 | H |  | IV | cytochrome c oxidase, cbb3-type, subunit II |
| *so2364* | -0.58 | 2.29 | H |  | IV | cytochrome c oxidase, cbb3-type, subunit I |
| *so2706* | 0.95 | 1.95 | H |  | IV | succinylarginine dihydrolase |
| *so2710* | 0.98 | 1.45 | J |  | IV | conserved hypothetical protein |
| *so2912* | 0.09 | 1.23 | H |  | IV | formate acetyltransferase |
| *so2913* | 0.12 | 1.44 | H |  | IV | pyruvate formate-lyase 1 activating enzyme |
| *so2915* | 0.12 | 1.12 | H |  | IV | acetate kinase |
| *so2916* | 0.64 | 1.46 | H |  | IV | phosphate acetyltransferase |
| *so2947* | -0.20 | 2.48 | J |  | IV | hypothetical protein |
| *so3085* | 0.38 | 3.28 | J |  | IV | conserved domain protein |
| *so3514* | -0.31 | 2.88 | J |  | IV | conserved hypothetical protein |
| *so3807* | 0.68 | 1.86 | T |  | IV | sterol desaturase family protein |
| *so3808* | 0.64 | 2.65 | J |  | IV | conserved hypothetical protein |
| *so3863* | 0.53 | 2.49 | R | -206 | IV | molybdenum ABC transporter, periplasmic molybdenum-binding protein |
| *so3865* | -0.27 | 1.44 | R |  | IV | molybdenum ABC transporter, ATP-binding protein |
| *so4281* | -0.75 | 1.61 | R |  | IV | potassium uptake protein KtrA, putative |
| *so4282* | -0.15 | 1.18 | R |  | IV | potassium uptake protein KtrB |
| *so4283* | -0.11 | 1.59 | C |  | IV | apbE family protein |
| *so4635* | 0.94 | 2.90 | D |  | IV | methyl-accepting chemotaxis protein |
| *so4694* | -0.70 | 2.68 | J |  | IV | hypothetical protein |
| *so0020* | 0.89 | -2.33 | I |  | V | fatty oxidation complex, beta subunit |
| *so0021* | 0.67 | -1.81 | I | -400 | V | fatty oxidation complex, alpha subunit |
| *so0092* | 0.02 | -2.43 | N |  | V | purine nucleoside phosphorylase |
| *so0142* | 0.06 | -2.58 | B |  | V | 3,4-dihydroxy-2-butanone 4-phosphate synthase |
| *so0441* | 0.48 | -1.29 | N |  | V | phosphoribosylamine--glycine ligase |
| *so0442* | 0.12 | -1.14 | N |  | V | phosphoribosylaminoimidazolecarboxamide formyltransferase/IMP cyclohydrolase |
| *so0756* | -0.84 | -2.18 | A |  | V | phospho-2-dehydro-3-deoxyheptonate aldolase, phe-sensitive |
| *so0845* | 0.84 | -1.61 | H |  | V | cytochrome c-type protein NapB |
| *so0848* | 0.99 | -1.83 | H |  | V | periplasmic nitrate reductase |
| *so0849* | -0.07 | -1.61 | H |  | V | napD protein |
| *so0923* | -0.73 | -2.42 | J |  | V | conserved hypothetical protein |
| *so1212* | -0.26 | -2.39 | J |  | V | hypothetical protein |
| *so1234* | -0.80 | -1.30 | H |  | V | torE protein |
| *so1236* | -0.54 | -2.68 | R |  | V | xanthine/uracil permease family protein |
| *so1274* | 0.33 | -2.45 | J |  | V | conserved hypothetical protein |
| *so1428* | 0.65 | -2.88 | C |  | V | outer membrane protein |
| *so1429* | 0.28 | -3.29 | H |  | V | anaerobic dimethyl sulfoxide reductase, A subunit |
| *so1430* | 0.01 | -4.03 | H |  | V | anaerobic dimethyl sulfoxide reductase, B subunit |
| *so1431* | 0.51 | -2.33 | J |  | V | conserved hypothetical protein |
| *so1432* | -0.14 | -2.31 | J |  | V | hypothetical protein |
| *so1778* | -0.35 | -1.08 | H |  | V | decaheme cytochrome c |
| *so1779* | -0.43 | -1.17 | H |  | V | decaheme cytochrome c |
| *so2469* | -0.30 | -1.61 | T |  | V | conserved hypothetical protein |
| *so2825* | 0.89 | -4.92 | J |  | V | hypothetical protein |
| *so2923* | -0.89 | -3.03 | R |  | V | sodium/glutamate symporter |
| *so3292* | -0.04 | -1.23 | N |  | V | GMP synthase |
| *so3293* | -0.46 | -1.24 | N |  | V | inosine-5-monophosphate dehydrogenase |
| *so3980* | -0.09 | -2.90 | H |  | V | cytochrome c552 nitrite reductase |
| *so4591* | -0.40 | -1.65 | H | -112 | V | tetraheme cytochrome c |
| *so4597* | -0.12 | -1.22 | R |  | V | heavy metal efflux system protein, putative |
| *so4598* | 0.40 | -1.13 | R |  | V | heavy metal efflux pump, CzcA family |
| *so4688* | 0.04 | -1.07 | C |  | V | glycosyl transferase, group 2 family protein |
| *so4700* | -0.01 | -2.09 | J |  | V | hypothetical protein |
| *so0220* | -1.36 | 0.03 | M |  | VI | ribosomal protein L11 |
| *so0221* | -1.42 | -0.09 | M |  | VI | ribosomal protein L1 |
| *so0222* | -1.21 | -0.26 | M |  | VI | ribosomal protein L10 |
| *so0223* | -1.24 | -0.60 | M |  | VI | ribosomal protein L7/L12 |
| *so0226* | -1.46 | -0.34 | M |  | VI | ribosomal protein S12 |
| *so0227* | -1.22 | -0.31 | M |  | VI | ribosomal protein S7 |
| *so0228* | -1.22 | -0.11 | M |  | VI | translation elongation factor G |
| *so0230* | -1.26 | 0.53 | M |  | VI | ribosomal protein S10 |
| *so0231* | -1.03 | 0.46 | M |  | VI | ribosomal protein L3 |
| *so0241* | -1.19 | -0.03 | M |  | VI | ribosomal protein L14 |
| *so0242* | -1.30 | -0.14 | M |  | VI | ribosomal protein L24 |
| *so0243* | -1.43 | -0.33 | M |  | VI | ribosomal protein L5 |
| *so0251* | -1.14 | 0.15 | L |  | VI | preprotein translocase, SecY subunit |
| *so0252* | -1.18 | -0.31 | M |  | VI | ribosomal protein L36 |
| *so0608* | -1.13 | 0.09 | H |  | VI | ubiquinol-cytochrome c reductase, iron-sulfur subunit |
| *so0609* | -1.10 | -0.25 | H |  | VI | ubiquinol-cytochrome c reductase, cytochrome b |
| *so1357* | -1.17 | 0.41 | M |  | VI | ribosomal protein S16 |
| *so1358* | -1.22 | 0.26 | Q |  | VI | 16S rRNA processing protein RimM |
| *so1629* | -1.15 | -0.39 | M |  | VI | ribosomal protein S2 |
| *so1630* | -1.43 | -0.47 | M |  | VI | translation elongation factor Ts |
| *so1631* | -1.08 | -0.54 | N |  | VI | uridylate kinase |
| *so1632* | -1.24 | -0.68 | M |  | VI | ribosome recycling factor |
| *so1770* | -1.90 | -0.22 | J |  | VI | glycerate kinase, putative |
| *so1771* | -1.63 | 0.65 | R |  | VI | permease, GntP family |
| *so2305* | -1.40 | -0.02 | O | -507 | VI | leucine-responsive regulatory protein |
| *so2402* | -1.03 | -0.20 | M | 21 | VI | ribosomal protein S1 |
| *so2907* | -2.41 | 0.53 | T | -179 | VI | TonB-dependent receptor domain protein |
| *so3300* | -1.16 | -0.78 | H |  | VI | cytochrome c |
| *so3301* | -1.38 | -0.66 | T |  | VI | flavocytochrome c flavin subunit |
| *so3667* | -1.53 | -0.06 | J |  | VI | conserved hypothetical protein |
| *so3669* | -1.40 | -0.36 | R |  | VI | heme transport protein |
| *so3927* | -1.04 | 0.11 | M |  | VI | ribosomal protein L9 |
| *so3928* | -1.44 | -0.21 | M |  | VI | ribosomal protein S18 |
| *so3937* | -1.03 | -0.33 | N |  | VI | adenylosuccinate synthetase |
| *so3939* | -1.41 | -0.67 | M |  | VI | ribosomal protein S9 |
| *so3983* | -1.41 | -0.35 | J |  | VI | conserved hypothetical protein |
| *so4246* | -1.62 | -0.81 | M |  | VI | ribosomal protein L33 |
| *so4247* | -1.58 | -0.43 | M |  | VI | ribosomal protein L28 |
| *so4248* | -1.63 | -0.43 | G |  | VI | DNA repair protein RadC |
| *so4416* | -3.47 | -0.51 | J |  | VI | hypothetical protein |
| *so4749* | -1.57 | -0.21 | H |  | VI | ATP synthase F1, alpha subunit |
| *so4750* | -1.45 | -0.20 | H |  | VI | ATP synthase F1, delta subunit |
| *so4751* | -1.61 | -0.01 | H |  | VI | ATP synthase F0, B subunit |
| *so4752* | -1.16 | 0.06 | H |  | VI | ATP synthase F0, C subunit |
| *so4753* | -1.08 | 0.05 | H |  | VI | ATP synthase F0, A subunit |
| *soa0003* | -1.08 | -0.78 | G |  | VI | type II restriction endonuclease, putative |
| *so0076* | -1.98 | -1.89 | J | -169 | VII | hypothetical protein |
| *so0314* | -3.03 | -2.79 | E | -522 | VII | ornithine decarboxylase, inducible |
| *so0370* | -1.94 | -2.48 | J |  | VII | conserved hypothetical protein |
| *so0403* | -3.01 | -4.09 | J |  | VII | hypothetical protein |
| *so0404* | -4.68 | -4.89 | J |  | VII | hypothetical protein |
| *so0919* | -2.96 | -2.52 | R |  | VII | serine transporter, putative |
| *so1003* | -1.36 | -2.41 | J |  | VII | hypothetical protein |
| *so1004* | -1.31 | -2.39 | J | -199 | VII | hypothetical protein |
| *so1418* | -1.09 | -1.98 | C |  | VII | apbE family protein |
| *so1424* | -2.19 | -1.50 | J |  | VII | hypothetical protein |
| *so1425* | -1.50 | -1.15 | J |  | VII | hypothetical protein |
| *so1427* | -1.77 | -4.37 | H | -246 | VII | decaheme cytochrome c |
| *so1568* | -5.14 | -3.93 | J |  | VII | hypothetical protein |
| *so1821* | -2.29 | -3.22 | R | -230 | VII | outer membrane porin, putative |
| *so1967* | -1.49 | -2.58 | J |  | VII | hypothetical protein |
| *so2389* | -4.44 | -3.20 | R |  | VII | multidrug resistance protein D |
| *so2427* | -3.88 | -1.23 | R |  | VII | TonB-dependent receptor, putative |
| *so2483* | -2.10 | -3.82 | A | -143 | VII | aspartate aminotransferase, putative |
| *so2736* | -1.24 | -2.11 | J |  | VII | conserved hypothetical protein |
| *so2865* | -2.46 | -1.84 | R |  | VII | L-lysine exporter, putative |
| *so3099* | -5.82 | -4.70 | R | -281 | VII | long-chain fatty acid transport protein, putative |
| *so3119* | -1.81 | -2.18 | J |  | VII | hypothetical protein |
| *so3275* | -2.32 | -1.20 | J |  | VII | hypothetical protein |
| *so3298* | -1.14 | -1.88 | J |  | VII | conserved hypothetical protein |
| *so3705* | -3.27 | -5.18 | E |  | VII | 5-methylthioadenosine nucleosidase/S-adenosylhomocysteine nucleosidase, putative |
| *so3706* | -2.46 | -3.20 | R |  | VII | NupC family protein |
| *so3969* | -4.14 | -3.25 | C |  | VII | OmpA family protein |
| *so4014* | -4.01 | -3.57 | R |  | VII | AcrB/AcrD/AcrF family protein |
| *so4015* | -2.94 | -3.60 | J |  | VII | conserved hypothetical protein |
| *so4159* | -2.59 | -1.48 | J |  | VII | hypothetical protein |
| *so4681* | -2.84 | -2.23 | C |  | VII | glycosyl transferase, group 1 family protein |
| ^a^ FC, functional category.  ^b^ BS, ArcA binding site.  ^c^ C, cluster. | | | | | | |
