# Supplementary material for: Probing regulon of ArcA in Shewanella oneidensis MR-1 by integrated genomic analyses
Source: BMC Genomics. 2008 Jan 25;9:42. doi: 10.1186/1471-2164-9-42 (PMC2262068; doi:10.1186/1471-2164-9-42)
Supplement: Additional file 4 — Genes in operons whose upstream regions contain an ArcA binding site. The data provided represent all operons whose upstream region contains a putative ArcA binding site. [file 1471-2164-9-42-S4.doc]

| **TABLE S3. Genes in operons whose upstream regions contain an ArcA binding site** | | | | | | |
| --- | --- | --- | --- | --- | --- | --- |
| Gene^a^ | Operon^b^ | Site | S^c^ | Z score | Motif | Function |
| *so0020* | *so0020-1* |  |  |  |  | Fatty oxidation complex, beta subunit |
| *so0021* |  | 400 | + | 2.45 | GTTAATAATAAATAT | Fatty oxidation complex, alpha subunit |
| *so0062* |  | 188 | - | 2.53 | GTGAAGTAAATGTGA | Hypothetical protein |
| *so0063* | *so0062-3* |  |  |  |  | Conserved hypothetical protein |
| *so0182* |  | 622 | + | 2.38 | GTTGATTAAATGGAA | Hypothetical protein |
| *so0208* |  | 44 | - | 2.44 | GATAAATAACTGTTA | RNA-binding protein |
| *so0266* | *so0266-9* | 71 | + | 2.63 | GTGAACAGAATGTTA | Cytochrome c-type biogenesis protein CcmF |
| *so0273* | *so0273-4* |  |  |  |  | Conserved hypothetical protein |
| *so0274* |  | 332 | - | 2.58 | GTGACTTAGATGTTA | Phosphoenolpyruvate carboxylase |
| *so0275* |  | 381 | + | 2.58 | GTGACTTAGATGTTA | N-acetyl-gamma-glutamyl-phosphate reductase |
| *so0276* | *so0275-9* |  |  |  |  | Acetylglutamate kinase |
| *so0277* | *so0275-9* |  |  |  |  | Ornithine carbamoyltransferase |
| *so0278* | *so0275-9* |  |  |  |  | Argininosuccinate synthase |
| *so0279* | *so0275-9* |  |  |  |  | Argininosuccinate lyase |
| ***so0308*** |  | 149 | - | 2.81 | GTTAATTAAAAGGGA | Conserved hypothetical protein |
| ***so0309*** |  | 21 | + | 2.81 | GTTAATTAAAAGGGA | Conserved hypothetical protein |
| ***so0315*** |  |  |  |  |  | Hypothetical protein |
| *so0316* |  | 452 | + | 2.43 | TTTAATTAACTGTAA | Conserved hypothetical protein TIGR00481 |
| *so0317* | *so0316-8* |  |  |  |  | Transcriptional regulator, AraC/XylS family |
| *so0318* | *so0316-8* |  |  |  |  | Conserved hypothetical protein |
| *so0343* |  | 53 | + | 2.31 | CTTAACTCAATGTGC | Aconitate hydratase 1 |
| *so0382* | *so0382-3* |  |  |  |  | Type I restriction-modification system, S subunit |
| *so0383* |  | 276 | + | 3.04 | GTTAATAAAATGTTT | Type I restriction-modification system, M subunit |
| ***so0387*** |  | 221 | - | 2.48 | GTTTATTTAATGTTT | Hypothetical protein |
| ***so0388*** |  | 84 | + | 2.48 | GTTTATTTAATGTTT | Site-specific recombinase, phage integrase family |
| *so0389* | *so0388-90* |  |  |  |  | Hypothetical protein |
| *so0390* | *so0388-90* |  |  |  |  | Hypothetical protein |
| *so0392* |  | 34 | - | 2.47 | GCTCATTAAATGATA | Lipoprotein, putative |
| *so0418* |  | 20 | - | 2.43 | ATTAATTAAATGATT | Hypothetical protein |
| *so0438* |  | 38 | - | 2.44 | GTTAATTACATTTAA | Oxidoreductase, short chain dehydrogenase/reductase family |
| ***so0450*** |  | 164 | - | 2.32 | GAAATTTAAATGTTA | Major facilitator family protein |
| ***so0451*** |  | 162 | + | 2.32 | GAAATTTAAATGTTA | Hypothetical protein |
| ***so0496*** |  | 223 | + | 2.45 | GTTAATTGAAGGTTT | Conserved hypothetical protein |
| ***so0497*** |  | 619 | - | 2.45 | GTTAATTGAAGGTTT | Hypothetical protein |
| *so0547* |  | 674 | + | 2.55 | GTTACTTAGATGATA | Conserved hypothetical protein |
| *so0548* |  | 108 | + | 2.55 | GTTACTTAGATGATA | DNA-binding protein, HU family |
| ***so0558*** |  | 354 | - | 2.91 | GTTAATTAAAAGGTA | SmtA protein |
| ***so0559*** |  | 55 | + | 2.91 | GTTAATTAAAAGGTA | MaoC domain protein |
| ***so0624*** |  | 228 | - | 2.62 | GTTAATCACATCTTA | Catabolite gene activator |
| ***so0625*** |  | 206 | + | 2.62 | GTTAATCACATCTTA | Conserved domain protein |
| *so0626* | *so0625-6* |  |  |  |  | Hypothetical protein |
| *so0650* |  | 360 | - | 2.55 | GTTGAGTAAATGTTG | Hypothetical protein |
| *so0651* | *so0650-2* |  |  |  |  | Conserved hypothetical protein |
| *so0652* | *so0650-2* |  |  |  |  | Prophage musO1, positive regulator of late transcription, putative |
| *so0756* |  | 164 | + | 2.74 | TTTAAAAATATGTTA | Phospho-2-dehydro-3-deoxyheptonate aldolase, phe-sensitive |
| *so0786* |  | 31 | - | 2.86 | TTTAAATAAATGTTA | Hypothetical protein |
| *so0787* |  | 674 | + | 2.71 | GTTATTTAAATGATG | Hypothetical protein |
| ***so0801*** |  | 139 | - | 2.58 | GTTAATTAGATTTTT | Conserved hypothetical protein |
| ***so0802*** |  | 225 | + | 2.58 | GTTAATTAGATTTTT | MATE efflux family protein |
| *so0866* |  | 136 | + | 2.94 | GTAATTTAAATGTTA | Serine protease, subtilase family |
| *so0810* | *so0810-1* |  |  |  |  | Ribokinase |
| *so0811* |  | 652 | + | 2.56 | GTTAATTTAAGGTCA | Inosine-uridine preferring nucleoside hydrolase family protein |
| *so0865* |  | 570 | - | 2.94 | GTAATTTAAATGTTA | Conserved hypothetical protein |
| *so0866* | *so0865-6* |  |  |  |  | Minor curlin subunit CsgB, putative |
| *so0867* |  | 328 | + | 2.94 | GTAATTTAAATGTTA | Serine protease, subtilase family |
| *so0877* |  | 204 | + | 2.99 | GTTAATTAAAGGCTA | Acetyltransferase, GnaT family |
| *so0916* |  | 150 | + | 2.35 | GTTAATAAAATATTG | Transcriptional regulator, MarR family |
| *so0922* |  | 119 | + | 2.54 | GCTAATTGAAGGTTA | Proton/glutamate symporter |
| *so0926* |  | 185 | - | 2.83 | ATTAATTAAAAGTTA | Acetyltransferase, GnaT family |
| *so0932* |  | 376 | + | 2.36 | GTCAATCAAGTGTTA | Phosphoglycerate kinase |
| *so0933* | *so0932-3* |  |  |  |  | Fructose-bisphosphate aldolase, class II, Calvin cycle subtype |
| ***so0948*** |  | 283 | + | 2.57 | GCTAATTAACTGCTA | Conserved hypothetical protein |
| ***so0949*** |  | 343 | - | 2.57 | GCTAATTAACTGCTA | Branched-chain amino acid transport system II carrier protein BrnQ |
| ***so0970*** |  | 429 | - | 2.52 | AATAATTAAATGTCA | Fumarate reductase flavoprotein subunit precursor |
| ***so0971*** |  | 86 | + | 2.52 | AATAATTAAATGTCA | Hypothetical protein |
| *so0972* | *so0971-2* |  |  |  |  | Conserved hypothetical protein |
| *so0990* |  | 348 | + | 2.72 | GTTTATTAAATGGAA | Urease domain protein |
| *so1003* |  |  |  |  |  | Hypothetical protein |
| ***so1004*** | *so1003-4* | 199 | - | 2.65 | GTTATTGAAATGTAA | Hypothetical protein |
| ***so1006*** |  | 243 | + | 2.65 | GTTATTGAAATGTAA | Dienelactone hydrolase family protein |
| *so1035* |  | 83 | + | 2.56 | GTTAATTTAATGCTT | Nicotinate-nucleotide--dimethylbenzimidazole phosphoribosyltransferase |
| *so1036* | *so1035-9* |  |  |  |  | Cobalamin 5-phosphate synthase |
| *so1037* | *so1035-9* |  |  |  |  | Cobinamide kinase/cobinamide phosphate guanylyltransferase |
| *so1038* | *so1035-9* |  |  |  |  | Cobyric acid synthase CobQ |
| *so1039* | *so1035-9* |  |  |  |  | Cob(I)alamin adenosyltransferase |
| *so1099* |  | 470 | + | 2.64 | GTTAGTTAACCGTTA | Bola protein |
| *so1215* |  | 165 | + | 2.36 | GTTAATTAGACGTAA | Outer membrane protein OmpK, putative |
| *so1282* |  | 476 | - | 2.49 | GTTAATCAACTGTTG | Isso11, transposase |
| *so1307* |  | 197 | - | 3.01 | GTTAACAAAGCGATA | Aquaporin Z |
| ***so1308*** |  | 226 | - | 3.17 | GTTACTTAAATGTTA | Hypothetical protein |
| ***so1309*** |  | 161 | + | 3.17 | GTTACTTAAATGTTA | Conserved hypothetical protein |
| *so1328* |  | 45 | + | 2.56 | GTTGATTAAATTTTT | Transcriptional regulator, LysR family |
| ***so1330*** |  | 672 | - | 2.67 | GTTTATTAAAATTTA | DNA mismatch repair protein MutH |
| ***so1331*** |  | 33 | + | 2.67 | GTTTATTAAAATTTA | Mutt/nudix family protein |
| *so1332* | *so1331-2* |  |  |  |  | Phosphoenolpyruvate-protein phosphotransferase PtsP |
| *so1398* |  | 512 | + | 2.65 | GTTATCTCAATGTTA | Hypothetical protein |
| ***so1399*** |  | 113 | + | 2.65 | GTTATCTCAATGTTA | Conserved hypothetical protein |
| ***so1400*** |  | 173 | - | 2.65 | GTTATCTCAATGTTA | Conserved hypothetical protein |
| *so1427* |  | 246 | - | 3.13 | GTTAATAAAATGTTT | Decaheme cytochrome c |
| *so1428* | *so1427-8* |  |  |  |  | Outer membrane protein |
| *so1429* | *so1427-8* |  |  |  |  | Anaerobic dimethyl sulfoxide reductase, A subunit |
| *so1430* | *so1427-8* |  |  |  |  | Anaerobic dimethyl sulfoxide reductase, B subunit |
| *so1436* |  | 655 | + | 2.44 | GTTCATTTAATGTTG | Isso11, transposase |
| *so1483* |  | 291 | + | 2.34 | TTTCACTAGATGTTA | Malate synthase A |
| *so1484* | *so1483-4* |  |  |  |  | Isocitrate lyase |
| *so1552* |  | 306 | - | 2.52 | GATAATTGAGTGTTA | TPR domain protein |
| *so1556* |  | 400 | - | 2.55 | GTAAGTTGAATGTTA | Conserved hypothetical protein |
| *so1558* |  | 146 | - | 2.62 | CTTAATTCAATCTTA | Phosphate regulon response regulator PhoB |
| *so1559* | *so1558-9* |  |  |  |  | Phosphate regulon sensor protein PhoR |
| *so1560* |  | 219 | + | 2.37 | TTTAATGTAATGTTA | Phosphate-binding protein |
| *so1563* |  | 609 | - | 2.37 | GATCATCAAATGTTA | Glutathione peroxidase, putative |
| ***so1621*** |  | 82 | + | 2.76 | GTTACTTTATTGTTA | Hypothetical protein |
| ***so1622*** |  | 56 | - | 2.76 | GTTACTTTATTGTTA | Conserved hypothetical protein |
| *so1623* | *so1622-3* |  |  |  |  | PTS system, glucose-specific IIBC component |
| *so1659* |  | 260 | - | 2.49 | ATTAATTTAATGATA | Decaheme cytochrome c |
| *so1661* |  | 219 | + | 3.12 | GTTAAATAATTGTTA | Transcriptional regulator, LySr family |
| *so1673* |  | 288 | + | 2.66 | GTAAATGAAATGTAA | Outer membrane protein OmpW, putative |
| ***so1713*** |  | 128 | - | 2.55 | GTGAATTAAATAATA | Hypothetical protein |
| ***so1714*** |  | 159 | + | 2.55 | GTGAATTAAATAATA | Hypothetical protein |
| ***so1806*** |  | 90 | - | 3.13 | GTTAATAAAATGTTT | Psp operon transcriptional activator |
| ***so1807*** |  | 102 | + | 3.13 | GTTAATAAAATGTTT | Phage shock protein A |
| *so1808* | *so1807-9* |  |  |  |  | Phage shock protein B |
| *so1809* | *so1807-9* |  |  |  |  | Phage shock protein C |
| *so1812* |  | 84 | + | 2.45 | GTTATTTAAAAGATA | Methionine gamma-lyase |
| *so1821* |  | 230 | + | 3.05 | GTTAATTTGATGTTA | Outer membrane porin, putative |
| *so1822* |  | 133 | + | 2.41 | GTTGATTAAAGGTTT | TonB-dependent receptor, putative |
| *so1824* |  | 151 | - | 2.51 | GTGAATAAAAAGTTA | Conserved hypothetical protein |
| *so1825* | *so1824-5* |  |  |  |  | MotA/TolQ/ExbB proton channel family protein |
| *so1868* |  | 281 | - | 2.61 | GTTTATTATATTTTA | Conserved hypothetical protein |
| *so1869* |  | 210 | + | 2.61 | GTTTATTATATTTTA | Hypothetical protein |
| *so1872* |  | 85 | - | 2.64 | ATTACTTAAATGCTA | Lipoprotein, putative |
| *so1879* |  | 593 | + | 2.74 | GTTAATGAAATGGTT | Dihydrodipicolinate synthase |
| *so1880* | *so1879-8* |  |  |  |  | Lipoprotein-34 NlpB |
| *so1944* |  | 65 | + | 2.96 | GTTAAGTAATTGTAA | Hypothetical protein |
| *so1961* |  | 114 | + | 2.63 | GTTAGCTAAATGGTA | Maltose O-acetyltransferase |
| *so2002* |  | 374 | + | 2.39 | GTTAATAAAATGCCA | Hypothetical protein |
| *so2037* |  | 555 | - | 2.32 | GTTACTTAAATCTTG | Site-specific recombinase, phage integrase family |
| *so2038* | *so2037-8* |  |  |  |  | Hypothetical protein |
| *so2095* | *so2095-9* |  |  |  |  | Hypothetical protein |
| *so2096* | *so2095-9* |  |  |  |  | Hydrogenase expression/formation protein |
| *so2097* | *so2095-9* |  |  |  |  | Quinone-reactive Ni/Fe hydrogenase, cytochrome b subunit |
| *so2098* | *so2095-9* |  |  |  |  | Quinone-reactive Ni/Fe hydrogenase, large subunit |
| ***so2099*** |  | 147 | + | 3.29 | GTTAATTAAATGTCA | Quinone-reactive Ni/Fe hydrogenase, small subunit precursor |
| ***so2100*** |  | 216 | - | 3.29 | GTTAATTAAATGTCA | Thioredoxin family protein |
| *so2101* | *so2100-1* |  |  |  |  | Lipoprotein, putative |
| ***so2116*** |  | 157 | - | 2.62 | GTTAATAAAAAGTAA | Acetyltransferase, GnaT family |
| ***so2117*** |  | 101 | + | 2.62 | GTTAATAAAAAGTAA | Methyl-accepting chemotaxis protein |
| *so2118* | *so2117-26* |  |  |  |  | Spoiiaa family protein |
| *so2119* | *so2117-26* |  |  |  |  | Response regulator |
| *so2120* | *so2117-26* |  |  |  |  | Chemotaxis protein CheY |
| *so2121* | *so2117-26* |  |  |  |  | Chemotaxis protein CheA |
| *so2122* | *so2117-26* |  |  |  |  | Purine-binding chemotaxis protein CheW |
| *so2123* | *so2117-26* |  |  |  |  | Methyl-accepting chemotaxis protein |
| *so2124* | *so2117-26* |  |  |  |  | Chemotaxis protein methyltransferase CheR |
| *so2125* | *so2117-26* |  |  |  |  | Chemotaxis protein CheD |
| *so2126* | *so2117-26* |  |  |  |  | Protein-glutamate methylesterase CheB |
| *so2163* |  | 292 | - | 2.72 | GTTACTTAAATCTTG | Hypothetical protein |
| *so2168* |  | 133 | + | 2.6 | ATTTATTAAATATTA | Isso1, transposase OrfA |
| *so2189* |  | 654 | + | 2.65 | ATTAACAAAATGTTA | Conserved hypothetical protein |
| *so2190* | *so2189-90* |  |  |  |  | CreA protein |
| *so2199* |  | 101 | - | 2.76 | GTGAATAAAATGTTT | Hypothetical protein |
| *so2210* |  | 571 | - | 2.61 | GTTACTTAAATCTTG | Isso11, transposase |
| ***so2240*** |  | 329 | - | 2.54 | ATTAATCAACTGTTA | Methyl-accepting chemotaxis protein |
| ***so2241*** |  | 31 | + | 2.54 | ATTAATCAACTGTTA | Conserved hypothetical protein |
| *so2256* |  | 572 | - | 2.59 | GTCAATTAAAGGTCA | Conserved hypothetical protein |
| *so2277* |  | 224 | - | 2.38 | ATTAATTAACTGTTC | 16 kda heat shock protein A |
| *so2305* |  | 507 | + | 2.91 | TTTAATTAAATCTTC | Leucine-responsive regulatory protein |
| *so2336* |  | 40 | - | 2.45 | GTTAATAAAATGGAA | Phosphoglucomutase, alpha-D-glucose phosphate-specific |
| *so2402* |  | 21 | - | 2.56 | GTTACTTTAATGTAA | Ribosomal protein S1 |
| *so2467* |  | 453 | - | 2.65 | GTTATTTAGCTGTTA | Adhesion-related protein |
| ***so2475*** |  | 80 | + | 2.37 | GTGAATGAAATGATA | Hypothetical protein |
| ***so2476*** |  | 168 | - | 2.37 | GTGAATGAAATGATA | Polysaccharide biosynthesis protein |
| *so2477* | *so2476-7* |  |  |  |  | Alcohol dehydrogenase, iron-containing |
| ***so2482*** |  | 44 | + | 2.34 | TTTAACTAAGTGTTA | Hypothetical protein |
| ***so2483*** |  | 143 | - | 2.34 | TTTAACTAAGTGTTA | Aspartate aminotransferase, putative |
| *so2610* |  | 673 | - | 2.95 | GTTACTTATTTGTTA | Hydrolase, TatD family |
| *so2651* |  | 474 | + | 2.56 | GTTGATTCGATGTTA | Hypothetical protein |
| *so2706* |  | 209 | + | 2.87 | GTTGAAAAAATGTAA | Succinylarginine dihydrolase |
| *so2718* |  | 531 | + | 2.41 | GTTAATTAATTTTTT | Conserved hypothetical protein |
| *so2727* |  | 220 | + | 2.42 | GTTATTCAAATGTAA | Cytochrome c3 |
| *so2746* | *so2746-7* |  |  |  |  | Conserved hypothetical protein |
| *so2747* |  | 28 | - | 2.32 | TAAAATTAAATGTTA | Peptidoglycan-associated lipoprotein |
| *so2774* | *so2774-81* |  |  |  |  | 3-oxoacyl-(acyl-carrier-protein) synthase II |
| *so2775* | *so2774-81* |  |  |  |  | Acyl carrier protein |
| *so2776* | *so2774-81* |  |  |  |  | 3-oxoacyl-(acyl-carrier-protein) reductase |
| *so2777* | *so2774-81* |  |  |  |  | Malonyl coa-acyl carrier protein transacylase |
| *so2778* | *so2774-81* |  |  |  |  | 3-oxoacyl-(acyl-carrier-protein) synthase III |
| *so2779* | *so2774-81* |  |  |  |  | Fatty acid/phospholipid synthesis protein PlsX |
| *so2780* | *so2774-81* |  |  |  |  | Ribosomal protein L32 |
| ***so2781*** |  | 106 | + | 2.89 | GTTAATTCAATGTTG | Conserved hypothetical protein |
| ***so2782*** |  | 166 | - | 2.89 | GTTAATTCAATGTTG | Maf protein, putative |
| *so2857* | *so2857-8* |  |  |  |  | Sodium/solute symporter family protein |
| *so2858* |  | 153 | + | 3.07 | GTAAATTCAATGTTA | Conserved hypothetical protein |
| *so2868* |  | 630 | + | 2.77 | GTTAATAAAATGGCA | Conserved hypothetical protein |
| *so2876* |  | 72 | - | 2.77 | ATTAATTAACTGTTC | Hypothetical protein |
| *so2907* |  | 179 | - | 3.13 | GTTAATAAAATGTTT | Tonb-dependent receptor domain protein |
| *so2938* |  | 78 | + | 2.75 | GATAATTTAATGTTT | Hypothetical protein |
| *so3045* |  | 133 | + | 2.65 | TGTAATTTAATGTTA | Hypothetical protein |
| *so3048* | *so3048-51* |  |  |  |  | Isoquinoline 1-oxidoreductase, beta subunit, putative |
| *so3049* | *so3048-51* |  |  |  |  | Isoquinoline 1-oxidoreductase, alpha subunit, putative |
| *so3050* | *so3048-51* |  |  |  |  | Conserved hypothetical protein |
| *so3051* | *so3048-51* | 219 | - | 2.34 | ATGAATTAAATATTA | Conserved hypothetical protein |
| *so3099* |  | 281 | - | 2.96 | GTTAATTAAATTATA | Long-chain fatty acid transport protein, putative |
| *so3106* |  | 127 | - | 3.12 | GTAAATTAATTGTTA | Cold-active serine alkaline protease |
| ***so3197*** |  | 187 | + | 2.52 | GTTATTTAAAAATTA | *Vacj* lipoprotein, putative |
| ***so3198*** |  | 46 | - | 2.52 | GTTATTTAAAAATTA | Hypothetical protein |
| *so3199* | *so3198-9* |  |  |  |  | Flhb domain protein |
| *so3259* |  | 188 | - | 2.45 | GTTGCTTAAATCTTA | Conserved domain protein |
| *so3260* | *so3259-60* |  |  |  |  | Hypothetical protein |
| ***so3277*** |  | 164 | + | 2.59 | GTTAATTTTATGTAA | Transcriptional regulator, TetR family |
| ***so3278*** |  | 41 | - | 2.59 | GTTAATTTTATGTAA | Conserved hypothetical protein |
| *so3279* | *so3278-9* |  |  |  |  | Acrb/acrd/acrf family protein |
| *so3285* | *so3285-6* |  |  |  |  | Cytochrome d ubiquinol oxidase, subunit II |
| *so3286* |  | 481 | + | 2.65 | GTTAATTTGCAGTTA | Phosphoribosylformylglycinamidine synthase |
| *so3299* |  | 66 | + | 2.33 | GTTAATTAATTTTGA | Pal/histidase family protein |
| *so3307* |  | 415 | + | 2.59 | GTTGATCTAATGTTA | Hypothetical protein |
| *so3342* |  | 181 | + | 2.57 | GTTAATTTGTTGTTA | Conserved hypothetical protein |
| *so3394* | *so3394-5* |  |  |  |  | Hypothetical protein |
| *so3395* |  | 14 | + | 2.41 | GTGAGTTAAAGGTTA | Hypothetical protein |
| *so3448* | *so3448-9* |  |  |  |  | Hypothetical protein |
| *so3449* |  | 42 | - | 2.66 | GTTAATTATTTGGTA | Conserved domain protein |
| ***so3479*** |  | 199 | - | 2.4 | TTTAATTAAAATTTA | Hypothetical protein |
| ***so3480*** |  | 310 | + | 2.4 | TTTAATTAAAATTTA | Conserved hypothetical protein |
| ***so3489*** |  | 41 | + | 2.92 | ATCAATTAAATGTTA | GGDEF domain protein |
| ***so3490*** |  | 310 | - | 2.92 | ATCAATTAAATGTTA | Conserved hypothetical protein |
| ***so3507*** |  | 90 | - | 3.02 | GTTAACTCAATGTTA | Conserved hypothetical protein |
| ***so3508*** |  | 46 | + | 3.02 | GTTAACTCAATGTTA | Hypothetical protein |
| ***so3541*** |  | 290 | + | 2.72 | GTTAATTTAATATTG | Sodium:alanine symporter family protein |
| ***so3542*** |  | 159 | - | 2.72 | GTTAATTTAATATTG | Conserved hypothetical protein |
| *so3548* |  | 249 | + | 2.86 | GTTAATTAACCTTTA | Conserved hypothetical protein |
| ***so3552*** |  | 90 | + | 2.63 | GTCAATTAAATGTAG | Von Willebrand factor type A domain protein |
| ***so3553*** |  | 125 | - | 2.63 | GTCAATTAAATGTAG | Sulfate permease family protein |
| *so3560* |  | 325 | + | 2.56 | GTTAATTGAGCGTTA | Peptidase, M16 family |
| *so3562* |  | 544 | - | 2.56 | GTTAATTGAGCGTTA | Proton/glutamate symporter, putative |
| ***so3564*** |  | 441 | + | 3.04 | GTTAATTAATTGTTG | Peptidyl-dipeptidase Dcp |
| ***so3565*** |  | 257 | - | 3.04 | GTTAATTAATTGTTG | 2,3-cyclic-nucleotide 2-phosphodiesterase |
| *so3600* |  | 43 | - | 2.62 | GTTAATCCAATATTA | Sulfate ABC transporter, permease protein |
| *so3601* | *so3600-2* |  |  |  |  | Sulfate ABC transporter, permease protein |
| *so3602* | *so3600-2* |  |  |  |  | Sulfate ABC transporter, ATP-binding protein |
| *so3619* |  | 139 | + | 2.6 | GTTATTCAAACGTTA | Hypothetical protein |
| *so3645* |  | 212 | + | 2.59 | GGTAAAAAAATGTTA | Hypothetical protein |
| ***so3654*** |  | 134 | - | 2.53 | GCTAATTAAGTGCTA | Uracil-DNA glycosylase |
| ***so3655*** |  | 50 | + | 2.53 | GCTAATTAAGTGCTA | Pilin, putative |
| *so3689* |  | 44 | + | 2.35 | GTTACTTTAAGGTTA | Sigma-54 dependent nitrogen response regulator |
| *so3728* |  | 582 | - | 2.48 | GTCAATTAGATTTTA | Uroporphyrin-III C-methyltransferase |
| *so3803* | *so3803-5* |  |  |  |  | Hypoxanthine phosphoribosyltransferase |
| *so3804* | *so3803-5* |  |  |  |  | Phenylacrylic acid decarboxylase, 3-octaprenyl-4-hydroxybenzoate carboxy-lyase, putative |
| ***so3805*** | *so3803-5* | 146 | + | 2.74 | GTTAATTAAATTTCG | UDP-N-acetylmuramate:L-alanyl-gamma-D-glutamyl-meso-diaminopimelate ligase |
| ***so3806*** |  | 19 | - | 2.74 | GTTAATTAAATTTCG | Hypothetical protein |
| *so3855* |  | 177 | - | 2.74 | GTTAATTGATTGTAA | Malate oxidoreductase |
| ***so3862*** |  | 32 | - | 2.75 | CTTGAGTAAATGTTA | Molybdenum transport regulatory protein mode |
| ***so3863*** |  | 206 | + | 2.75 | CTTGAGTAAATGTTA | Molybdenum ABC transporter, periplasmic molybdenum-binding protein |
| *so3864* | *so3863-5* |  |  |  |  | Molybdenum ABC transporter, permease protein |
| *so3865* | *so3863-5* |  |  |  |  | Molybdenum ABC transporter, ATP-binding protein |
| *so3867* |  | 527 | - | 2.58 | GTTAAGTAAAGGTTG | Transcriptional regulator, Cro/CI family |
| *so3889* | *so3889-90* |  |  |  |  | Hypothetical protein |
| ***so3890*** |  | 170 | + | 2.37 | CTTAATAAAATATTA | Methyl-accepting chemotaxis protein |
| ***so3891*** |  | 159 | - | 2.37 | CTTAATAAAATATTA | Conserved hypothetical protein |
| *so3910* |  | 152 | - | 2.38 | TTTTAATAAATGTTA | Hypothetical protein |
| *so3933* |  | 299 | - | 2.45 | GTGTATTTAATGTTA | Membrane protein, putative |
| *so4024* |  | 607 | - | 2.32 | GTTAAATTAATTTTA | Isso4, transposase |
| *so4025* | *so4024-5* |  |  |  |  | Ompa-like transmembrane domain protein |
| *so4230* |  | 219 | + | 2.41 | GTTAATTAGATTTTT | Glycerol kinase |
| *so4233* | *so4233-6* |  |  |  |  | 3-isopropylmalate dehydratase, small subunit |
| *so4234* | *so4233-6* |  |  |  |  | 3-isopropylmalate dehydratase, large subunit |
| *so4235* | *so4233-6* |  |  |  |  | 3-isopropylmalate dehydrogenase |
| *so4236* | *so4233-6* | 382 | - | 2.68 | ATAAATTAAATGTCA | 2-isopropylmalate synthase |
| *so4245* |  | 178 | + | 2.56 | GTTAAAAAAATGTGA | Amino-acid acetyltransferase |
| *so4273* |  | 9 | + | 3.08 | GGTAATTAATTGTTA | Hypothetical protein |
| ***so4274*** |  | 97 | - | 2.72 | GTTAATTATATTTAA | Undecaprenol kinase, putative |
| ***so4275*** |  | 81 | + | 2.72 | GTTAATTATATTTAA | Hypothetical protein |
| *so4304* |  | 47 | - | 2.35 | GTTAAATAATTGCTA | Conserved hypothetical protein |
| ***so4340*** |  | 210 | - | 2.69 | GTTAATTAATTATTT | Conserved hypothetical protein |
| ***so4341*** |  | 479 | + | 2.69 | GTTAATTAATTATTT | Hypothetical protein |
| *so4356* |  | 170 | + | 2.77 | GTTAAATAAATTTAA | Conserved domain protein |
| *so4361* | *so4361-2* |  |  |  |  | Hypothetical protein |
| *so4362* |  | 261 | + | 2.81 | GGTGATTAAATATTA | Conserved hypothetical protein |
| *so4364* |  | 321 | - | 2.34 | GGTGATTAAATATTA | ATP-dependent DNA helicase RecG |
| *so4366* |  | 195 | - | 2.62 | GTTAATAAAATGCAA | Conserved hypothetical protein |
| *so4367* | *so4366-79* |  |  |  |  | Acyltransferase family protein |
| *so4368* | *so4366-79* |  |  |  |  | Acyl carrier protein, putative |
| *so4369* | *so4366-79* |  |  |  |  | Acyl carrier protein, putative |
| *so4370* | *so4366-79* |  |  |  |  | Membrane protein, putative |
| *so4371* | *so4366-79* |  |  |  |  | Conserved hypothetical protein |
| *so4372* | *so4366-79* |  |  |  |  | Thioester dehydrase family protein |
| *so4373* | *so4366-79* |  |  |  |  | Glycosyl transferase, group 2 family protein |
| *so4374* | *so4366-79* |  |  |  |  | Histidine ammonia-lyase, putative |
| *so4375* | *so4366-79* |  |  |  |  | Conserved hypothetical protein |
| *so4376* | *so4366-79* |  |  |  |  | Hypothetical protein |
| *so4377* | *so4366-79* |  |  |  |  | Membrane protein, putative |
| *so4378* | *so4366-79* |  |  |  |  | FAD-binding protein |
| *so4379* | *so4366-79* |  |  |  |  | Hypothetical protein |
| ***so4401*** |  | 62 | + | 2.61 | GTAAATTAAGTGTTT | Ribonuclease BN |
| ***so4402*** |  | 31 | - | 2.61 | GTAAATTAAGTGTTT | Hypothetical protein |
| *so4403* | *so4402-3* |  |  |  |  | Hypothetical protein |
| *so4445* |  | 631 | - | 2.65 | GTTATTTAAATCGTA | Response regulator/sensor histidine kinase |
| *so4454* |  | 61 | + | 2.49 | GTTCAATACATGTTA | Methyl-accepting chemotaxis protein |
| *so4457* |  | 63 | - | 2.76 | GTTGCCTAAATGTTA | GGDEF domain protein |
| *so4463* |  | 77 | + | 2.56 | GTAAATAAAATGTTT | Prolyl 4-hydroxylase, alpha subunit domain protein |
| *so4465* |  | 231 | - | 2.56 | GTAAATAAAATGTTT | Conserved domain protein |
| *so4480* |  | 562 | + | 2.73 | GTTAAATAAAGGTAA | Aldehyde dehydrogenase |
| *so4497* |  | 634 | - | 2.57 | CTTAATTAAAGGCTA | Isso5, transposase |
| *so4501* |  | 84 | + | 2.71 | CTTAATTAAAGGCTA | Hypothetical protein |
| *so4556* |  | 521 | - | 2.72 | GTTACTTAAATGGTG | Transcriptional regulator, LysR family |
| *so4570* |  | 147 | - | 3.13 | GTTAATAAAATGTTT | Conserved domain protein |
| *so4589* |  | 208 | - | 2.59 | GTCAATGAGATGTTA | Transcriptional regulator, AraA family |
| ***so4591*** |  | 112 | - | 2.64 | ATTAATTAAAACTTA | Tetraheme cytochrome c |
| ***so4592*** |  | 312 | + | 2.64 | ATTAATTAAAACTTA | Hypothetical protein |
| *so4603* |  | 631 | - | 2.32 | GTTACGTAAATGTTT | LexA repressor |
| *so4604* | *so4603-4* |  |  |  |  | Conserved hypothetical protein |
| *so4621* |  | 70 | - | 2.72 | ATTATTTAACTGTTA | Hypothetical protein |
| *so4622* | *so4622-4* |  |  |  |  | Sensor histidine kinase |
| *so4623* | *so4622-4* |  |  |  |  | DNA-binding response regulator |
| *so4624* | *so4622-4* | 347 | + | 2.63 | TTGAATTAAATGTTG | Transcriptional regulator, LuxR family |
| ***so4707*** |  | 101 | + | 2.41 | GGTAATTAAAAGTTG | Isso4, transposase |
| ***so4708*** |  | 221 | - | 2.41 | GGTAATTAAAAGTTG | Conserved hypothetical protein |
| *so4730* |  | 420 | + | 2.58 | GTTAATTAAAGATCA | Oxygen-independent coproporphyrinogen III oxidase |
| *so4734* |  | 284 | - | 2.43 | GGTAATTAATTGTAA | Sensory box protein |
| *soa0002* |  | 38 | - | 2.95 | GATAATTACATGTGA | Type II restriction-modification system activator, putative |
| *soa0003* | *soa0002-3* |  |  |  |  | Type II restriction endonuclease, putative |
| *soa0065* |  | 169 | - | 2.88 | CTTAATTAATTGTTA | Isso1, transposase OrfA |
| *soa0140* |  | 56 | + | 2.51 | GTCAATTAAATATCA | Hypothetical protein |
| *soa0141* | *soa0140-1* |  |  |  |  | Hypothetical protein |
| *soa0146* |  | 140 | + | 2.65 | GTAATTTTAATGTTA | Isso1, transposase OrfA |
| *soa0147* |  | 683 | - | 2.65 | GTAATTTTAATGTTA | Hypothetical protein |
| ^a^ In bold, genes next to each other share the same binding motif.  ^b^ Binding motif for the first gene of each operon was shown.  ^c^ Strand. | | | | | | |
