# Supplementary material for: Probing regulon of ArcA in Shewanella oneidensis MR-1 by integrated genomic analyses
Source: BMC Genomics. 2008 Jan 25;9:42. doi: 10.1186/1471-2164-9-42 (PMC2262068; doi:10.1186/1471-2164-9-42)
Supplement: Additional file 5 — Primers used in this study. The data provided represent all primers used in this study. [file 1471-2164-9-42-S5.doc]

| **TABLE S4. Primers used in this study** | |
| --- | --- |
| Mutagenesis |  |
| SO3988-5-F | GCTTCTGTCGATAAACACGGC |
| SO3988-5-R | TGTTTAAACTTAGTGGATGGGCCTCAGTTACCACATACCC |
| SO3988-3-F | CCCATCCACTAAGTTTAAACACCAGATACGCCAGAAATCATCG |
| SO3988-3-R | TTACCCAATACTTAGTTCAGCAAGG |
|  |  |
| Complementation |  |
| SO3988-COM-F/R | CTGCGAGCTCACCTTATGCTG/CTCGATAGAGAGCTCAAAAACG |
|  |  |
| Real-time qRT-PCR |  |
| SO0186-RT-F/R | GCGGCGCCGATTGGTACCG/GAATGGTGCCATAGGTAAG |
| SO0314-RT-F/R | AACCACAAATCCAACCACC/CGATACCGCCAATAAAGC |
| SO0343-RT-F/R | CCAACAAACGCTAGACTACC/ATCATCGCTCCACAAACC |
| SO0848-RT-F/R | CTGCGGATGAAAGACGGC/TGCTGTGTTTCCACTTAGCG |
| SO1232-RT-F/R | GGCTTACACGCAGGACAAAC/TCACAAAATTACCGTGCATCC |
| SO2074-RT-F/R | GAACAACGTCTTATCGC/AATCCACTACGCCATCCATC |
| SO2099-RT-F/R | ACTCGATATGTTCAACCGTCC/TTCTTCAACGAACTCACCTGC |
| SO2913-RT-F/R | CTCGCCGAGTTTATCAAACC/AGCTTTCTCCCATTGCTTCC |
|  |  |
| EMSA |  |
| ArcA-up | GGGGACAAGTTTGTACAAAAAAGCAGGCTTCATGCAAAATCCGCACATTCTGATCG |
| ArcA-down | GGGGACCACTTTGTACAAGAAAGCTGGGTNGTCTTCTAAGTTACCGCAGAAACG |
| SO0011-EMSA-F/R SO0266-EMSA-F/R | GGTATAATCGGGGAGTTTTTA/TTCTCTGACATATTATTCTCTC  GGGCACTGGTTCACAAATTGA/TGGTCTTTACGGGCAACACCT |
| SO0314-EMSA-F/R | GGATCATATTTTTATTGATA/CTAACAGATAAGCTTGCGGC |
| SO0396-EMSA-F/R | CAGTTTAGGTTCAAAGATCG/CGTTACGCTTATCATCCTGC |
| SO0397-EMSA-F/R | TAAATCCTGAGATTGGCCCA/CTTAGTTTGATTGCCATTGC |
| SO0432-EMSA-F/R | GTTTCGAGAGTGACAGGCAC/TTTACGATATGCTTCTAGCA |
| SO0756-EMSA-F/R | CGACGTCCTTGCGACTATCAT/AGGATCGCAATAGGGGGAAGT |
| SO0806-EMSA-F/R | GGCTGACAAAGACGTCACAAA/CATCCCATTAGGCCGTAGGAG |
| SO0866-EMSA-F/R | GCCAACGGTGAGAGTTTTATCG/AGACAATTGGCCGAAAGGGTAT |
| SO1307-EMSA-F/R | GGAACAACCATGGGTTAGCAA/GTTTTGCATGCCCAGATTGTTA |
| SO1427-EMSA-F/R | attggcatgattgaattgtgagc/ccccaaaaacatcagaaacatgc |
| SO1623-EMSA-F/R | TACTCCGTCCCACATGCGTA/CACTTGGCGGTTTAACAAGGAG |
| SO1661-EMSA-F/R | CCACACCATACCGATAAAGAAGC/GCAACAATTATGCACGGTATGC |
| SO1806-EMSA-F/R | TTGATGTTGGTCTCGCTTATGG/TACTTCAAGCAGGGCGTTTGAT |
| SO1821-EMSA-F/R | CTGATCTTTAGTAAGCGAAT/TAGCGCAGTGGCGACTATCG |
| SO1926-EMSA-F/R | TCTATCGAGTCGTTCCCTGGT/CACGTTCTGCTCACTCAGCTC |
| SO1930-EMSA-F/R | GCGAGCTGTGTAATGCAAGAA/TGATGCCTTGGTGCATTTCTA |
| SO1944-EMSA-F/R | AGCGGGAGTTTAACGCACTTT/TCAAACGCAAGAATGAGACGAT |
| SO2099-EMSA-F/R | catgggtcaaccttttgttatgg/gtcccattagcaaaaccgaaatc |
| SO2389-EMSA-F/R | GACGTTGTGTTTGGCAGCAT/GCAGTTCAAGTGTTAATCAT |
| SO2460-EMSA-F/R | CGCGAACTACGCTGAATCGG/GTCCTTTCTTGCTAATTGG |
| SO2629-EMSA-F/R | CACACGTTCTTCAACGTGCTCT/AGGGCAAATAAACCCGAATCC |
| SO2706-EMSA-F/R | GAAGGTGGAAATTGGCTCCATA/CGCTTCAAAGTGCTTCATTGC |
| SO2727-EMSA-F/R | TTGTGCCGAATTACGCTAGGA/TCTTGTCAGGGGTGACTTCCA |
| SO3099-EMSA-F/R | ACAGCCCAAATACCCCTTCCT/GCGATGGCAACAGTAAGCAG |
| SO3106-EMSA-F/R | GCCCTCACCTTACGAGTCACC/TTGCAATCGCTAAGCTGGATAAA |
| SO3507-EMSA-F/R | CGGTACCGTCTGCGGTATAGAT/GCCATTTCTTCATCATGACGTT |
| SO3565-EMSA-F/R | GATCGCCGAATACAAACAAAGC/AATTCATGCGCTGAACACGAT |
| SO3659-EMSA-F/R | CCGCGATGACACTAACGACTC/GCCGACGAGTAAAGCGAGAAT |
| SO3855-EMSA-F/R | GCTCAGGTGGAAGAAACCAA/GGTCCTGCAAACGGAAGATA |
| SO4245-EMSA-F/R | GGTCTTTTTCCGCCTTTAGTCA/CCTCATATTGAAGCTGGCAACC |
